# Machine learning-based on cytotoxic T lymphocyte evasion gene develops a novel signature to predict prognosis and immunotherapy responses for kidney renal clear cell carcinoma patients

**DOI:** 10.3389/fimmu.2023.1192428

**Published:** 2023-07-31

**Authors:** Mei Chen, Zhenyu Nie, Denggao Huang, Yuanhui Gao, Hui Cao, Linlin Zheng, Shufang Zhang

**Affiliations:** Central Laboratory, Affiliated Haikou Hospital of Xiangya Medical College, Central South University, Haikou, China

**Keywords:** machine learning, immune evasion, STAT2, drug resistance, immunotherapy

## Abstract

**Background:**

Immunotherapy resistance has become a difficult point in treating kidney renal clear cell carcinoma (KIRC) patients, mainly because of immune evasion. Currently, there is no effective signature to predict immunotherapy. Therefore, we use machine learning algorithms to construct a signature based on cytotoxic T lymphocyte evasion genes (CTLEGs) to predict the immunotherapy responses of patients, so as to screen patients effective for immunotherapy.

**Methods:**

In public data sets and our in-house cohort, we used 10 machine learning algorithms to screen the optimal model with 89 combinations under the cross-validation framework, and 101 published signatures were collected. The relationship between the CTLEG signature (CTLEGS) and clinical variables was analyzed. We analyzed the role of CTLES in other types of cancer by pan-cancer analysis. The immune cell infiltration and biological characteristics were evaluated. Moreover, the response to immunotherapy and drug sensitivity of different risk groups were investigated. The key gene closely related to the signature was identified by WGCNA. We also conducted cell functional experiments and clinical tissue validation of key gene.

**Results:**

In public data sets and our in-house cohort, the CTLEGS shows good prediction performance. The CTLEGS can be regard as an independent risk factor for KIRC. Compared with 101 published models, our signature shows considerable superiority. The high-risk group has abundant infiltration of immunosuppressive cells and high expression of T cell depletion markers, which are characterized by immunosuppressive phenotype, minimal benefit from immunotherapy, and resistance to sunitinib and sorafenib. The CTLEGS was also strongly correlated with immunity in pan-cancer. Immunohistochemistry verified that T cell depletion marker LAG3 is highly expressed in high-risk groups in the clinical in-house cohort. The key CTLEG STAT2 can promote the proliferation, migration and invasion of KIRC cell.

**Conclusions:**

CTLEGS can accurately predict the prognosis of patients and their response to immunotherapy. It can provide guidance for the precise treatment of KIRC and help clinicians identify patients who may benefit from immunotherapy.

## Introduction

Kidney cancer accounts for 4% of newly diagnosed tumors and 2% of deaths; it is more common in men than women (1). Kidney renal clear cell carcinoma (KIRC) is the main histological subtype of kidney cancer. KIRC has intratumoral and immune heterogeneity, thereby posing a major challenge in anticancer therapy and contributing to clinical differences in patient response to therapy (2, 3). Targeted drugs are the main treatment for patients with kidney cancer; however, many patients are resistant to these drugs (4). Immunocheckpoint inhibitor (ICI) combination therapy has become the first-line treatment for advanced kidney cancer (5, 6). Immunotherapy is one of the therapies with the greatest potential for providing radical treatment opportunities for tumor patients. Programmed cell death protein 1 (PDCD-1)/programmed death-ligand 1 (PD-L1) inhibitors, commonly used for KIRC treatment, improve the clinical outcome of patients; however, not all patients respond to immunotherapy (7–12). The heterogeneity of the tumor microenvironment makes patients benefit differently from ICI, and many patients develop drug resistance (13). Therefore, constructing a reliable signature for evaluating the immune microenvironment and predicting the efficacy of immunotherapy in patients is vital.

T cells are the main immune cells of KIRC, and T cells with high expression of PDCD1 are the depletion phenotype of KIRC (14). Cytotoxic T lymphocytes (CTLs), also known as CD8^+^T cells, are the main cells of anticancer immunity and the main focus of cancer immunotherapy (15). ICI resistance occurs when the immune system overactivates CD8^+^T cells and differentiates them into terminal depletion phenotype; moreover, the expression of coinhibitory receptors PDCD1, T cell immunoglobulin and mucin domain 3 (TIM-3), and lymphocyte-activation gene 3 (LAG3) increases (16). Many immunotherapies require CD8^+^T cells to recognize and kill tumor cells, and immune invasion is the main reason for ICI resistance (17). CEP55 is an important diagnostic marker for tumors, such as renal clear cell carcinoma (18) and intrahepatic cholangiocarcinoma (19). PTPN2 loss can increase antigen presentation and sensitivity to CD8^+^T cells, making tumor cells sensitive to immunotherapy (20). Tumors can evade immune clearance by regulating the expression and function of TRAF2. Cells overexpressing TRAF2 have stronger resistance to CD8^+^T cell killing, and the combined targeting of TRAF2/cIAP1 increases the therapeutic effect of immune checkpoint inhibitors (21). JAK1, JAK2, and B2M are frequently mutated in patients with immune checkpoint resistance (22). These genes are cytotoxic T lymphocyte-evasion genes (CTLEGs) identified by Lawson et al. (23). At present, no effective biomarker or model for predicting the response of KIRC patients to immunotherapy exists. Previous studies only used Lasso or Cox to construct signatures, and the prediction efficiency of the signatures was limited (24–26). In the era of big data, machine learning has become increasingly important in mining and analyzing high-throughput sequencing data. Therefore, it is worthwhile to explore how to use machine learning to develop signatures to predict the prognosis of patients and the response of immunotherapy in combination with CTLEGs.

In the present study, we used 10 machine learning algorithms, namely, Lasso, survival support vector machine (survival-SVM), Ridge, CoxBoost, elastic network (Enet), random survival forest (RSF), stepwise Cox (StepCox), supervised principal components (SuperPC), partial least squares regression for Cox (plsRcox), and generalized boosted regression modeling (GBM), to screen key CTLEGs in a cross-validation framework. A robust CTLEG signature (CTLEGS) was developed and validated in three public databases and a clinical in-house cohort to reveal tumor microenvironment characteristics, evaluate the CTLEGS’s response to drugs and immunotherapy, correlate with clinical features, and perform pan-cancer analyses. Finally, signal transducer and activator of transcription 2 (STAT2) was choosed and verified *in vitro*.

## Materials and methods

### Patient sources and data processing

The transcriptome data and clinical information of the KIRC cohort were downloaded from The Cancer Genome Atlas (TCGA) database (https://portal.gdc.cancer.gov/). The sequencing data and clinical information of the GSE22541 cohort were downloaded from the Gene Expression Omnibus (GEO) database (https://www.ncbi.nlm.nih.gov/geo/). The probes were converted into gene symbols through gene annotation. The sequencing data and clinical information of the E-MTAB-1980 cohort were downloaded from the ArrayExpress database (https://www.ebi.ac.uk/arrayexpress/). The samples with a survival time greater than 0 were included in the study. Finally, this study included 528 patients from the TCGA cohort, 101 patients from the E-MTAB-1980 cohort, and 40 samples from the GSE22541 cohort. Basic clinical characteristics of those cohorts are summarized in **Supplementary Table 1**. The single-cell sequencing data set GSE207493 was downloaded from the GEO database, including 19 samples. The “harmony” package was used to eliminate the differences between samples when the data were integrated. The “Seurat” package was used for analysis. The cells were annotated as nonhematopoietic cells, T cells, natural killer (NK) cells, macrophage cells, monocyte cells, dendritic cells, B cells, and mast cells. The expression and clinical data of anti-PD-1 therapy patients (CheckMate 010 + 025 + 090) were obtained from published literature (27). The transcriptomic data and clinical information of pan-cancer were downloaded from the University of California Santa Cruz database (https://xena.ucsc.edu/). Clinical Proteomic Tumor Analysis Consortium (CPTAC, https://proteomics.cancer.gov/programs/cptac) contains a large amount of proteomic data, PDC000127 cohort was downloaded from the CPTAC.

### Sample collection of the Affiliated Haikou Hospital of Xiangya Medical College (AHHXMC) cohort

From September 2016 to September 2022, we collected the cancer tissues of 80 KIRC patients from AHHXMC. The diagnosis was confirmed by pathologists. All patients obtained informed consent and approval from the hospital ethics committee. Overall survival (OS) time was obtained by follow-up. The clinical information of patients and the International Metastatic Renal Cell Carcinoma Database Consortium score of the metastatic patient are shown in **Supplementary Table 1**.

### Identification of gene clusters

We obtained 182 CTLEGs from published literature (23) (**Supplementary Table 2**). The “limma” package was used to screen the differentially expressed CTLEGs. Univariate Cox regression analysis was used to screen prognostic genes. Prognostic genes were subjected to unsupervised clustering analysis using the “ConsensusClusterPlus” package. The “survival” and “survminer” packages were used to perform survival analyses.

### Assessment of immune cell infiltration

Based on TCGA RNA sequencing data, the infiltration of immune cells in each group was analyzed by single cell gene set enrichment analysis (ssGSEA) (28), Microenvironment Cell Populations-counter (MCPcounter) (29), Estimating the Proportion of Immune and Cancer cells (EPIC) (30), XCELL (31), CIBERSORT (32), Estimation of STromal and Immune cells in MAlignant Tumours (ESTIMATE) (33), Tumor Immune Estimation Resource (TIMER) (34) and QUANTISEQ (35) algorithms. Drug sensitivity was predicted with the “pRRophic” package.

### Construction and validation of CTLEGS

TCGA-KIRC was used as the training set, whereas E-MTAB-1980, GSE22541, and AHHXMC cohorts were used as the testing set. Under the cross-validation framework, one algorithm was used to select the prognostic differentially expressed genes (DEGs), and the other algorithm was used to construct the prognostic signature. A total of 89 combinations of Lasso, Ridge, Enet, StepCox, survivalSVM, CoxBoost, SuperPC, plsRcox, RSF, and GBM filtered the model combination with the highest C-index. Finally, the “ComplexHeatmap” package was used to draw the heat map to visualize the evaluation results of the signature. The “timeROC” package was used to draw the receiver operator curves (ROC) to evaluate the prediction performance of the signature. The “rms” package was used to draw a nomogram.

### Enrichment analysis

The “clusterProfiler” package was used to conduct GO and KEGG enrichment analysis on the DEGs between high- and low-risk groups. “c2. cp. kegg. v7.5.1. symbols. Gmt” was chosed in GSEA.

### Identification of key CTLEGs related to signature

Based on the TCGA expression data, the “weighted correlation network analysis (WGCNA)” package was used to construct the coexpression network, identify the modules related to CTLEGS, screen the related modules, intersect with the modeling genes, and obtain the key CTLEG.

### Transcriptome sequencing

The RNA of the sample was isolated and purified according to the instructions of TRIzol (Thermofisher, 15596018). Oligo magnetic beads (Dynabeads Oligo [dT], Thermo Fisher, USA) were used to capture the mRNA with PolyA through two rounds of purification. mRNA fragmentation was conducted under high-temperature conditions using Magnesium RNA Fragmentation Module (NEB, USA). The fragmented RNA was synthesized into cDNA. A dUTP solution (Thermo Fisher, CA, USA) was added to the double-stranded DNA. The end of the double-stranded DNA was completed to the flat end. PCR library enrichment was performed after the connector connection. Finally, Illumina NovaseqTM 6000 (LC Bio-Technology Co., Ltd. Hangzhou, China) was used to carry out two-terminal sequencing according to the standard operation. The sequencing mode was PE150.

### Quantitative real-time PCR

We extracted RNA from 64 cancer tissues with the corresponding RNA sequencing data and 16 paired paracancer tissues. The reagents and procedures used were described in our previous study (36). The primer sequences used in the present study are shown in **Supplementary Table 3**.

### Immunohistochemistry (IHC)

The paraffin-embedded tumor tissues of 55 of 80 samples used for RNA sequencing were collected for IHC. After the paraffin sections were dewaxed, antigen repair was performed. The sections were placed in a 3% hydrogen peroxide solution and incubated at room temperature away from light. The rabbit serum was used to close at room temperature for 30 min. The sections were diluted with anti-LAG3 primary antibody (ab209236, Abcam, England, 1:1000) and incubated overnight in a wet box at 4°C. After decolorization with PBS, the secondary antibody (GB23303, Servicebio, Wuhan, China, 1:200) was added and incubated at room temperature for 50 min. DAB was added for color development. Hematoxylin was re-dyed, and the film was sealed and photographed under a microscope.

### Cell function experiment

769-P and 786-0 were selected for the experiment. Small interfering RNAs (siRNA) targeted STAT2 was used to transfect cells. Knockdown efficiency was detected 48 h later. For the clonal formation experiment, 769-P (1500 cells) and 786-0 (1000 cells) were planted in six-well plates and stained with crystal violet 14 days later. For the Cell Counting Kit-8 (CCK-8) assay, 769-P (3000 cells) and 786-0 (1500 cells) were planted in 96-well plates. Then they were detected at 0, 24, 48, and 72 h. For the transwell migration assay, 200 ul serum-free cells were planted in the upper chamber, and 600 ul complete culture medium was added outside the lower chamber. After 24 h of culture, crystal violet staining was conducted, and microscopically photographed. Transwell invasion assay requires the placement of Matrigel in the upper chamber. For the wound healing experiment, a 200 ul gun tip was used to make scratches when the cells were overgrown. The pictures were taken under the microscope at 0 and 24 h. Detailed steps were described in our previous study (37).

### Statistical analyses

Data processing and statistical analysis were performed in R 4.1.2 and GraphPad Prism 8.0.2. Paired or unpaired t-test and Wilcoxon test were used to compare the difference between the two groups. Kaplan-Meier and log-rank test were used to perform survival analyses. Prognostic CTLEGs were screened by univariate cox regression analysis. Spearman was used for correlation analysis. *P*<0.05 was considered a significant difference.

## Results

### Identification of key CTLEGs in the KIRC

**Figure 1:**
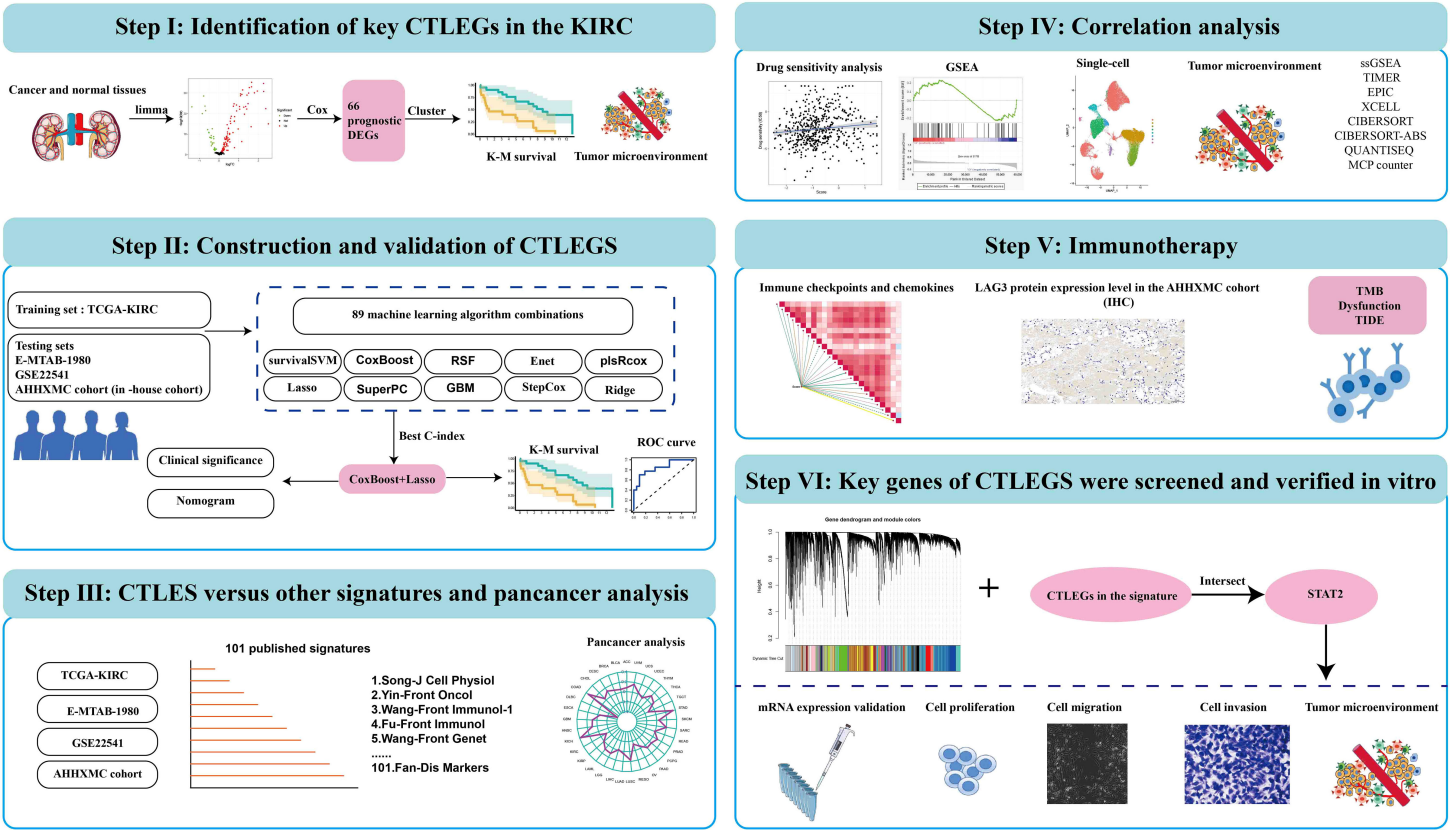
Flowchart of cytotoxic T lymphocyte-evasion genes signature for predicting prognosis and immunotherapy response in KIRC patients. CTLEGs, cytotoxic T lymphocyte evasion genes; KIRC, kidney renal clear cell carcinoma; DEGs, differentially expressed genes; TCGA, The Cancer Genome Atlas; survival-SVM, survival support vector machine; Enet, elastic network; plsRcox, partial least squares regression for Cox; RSF, random survival forest; SuperPC, supervised principal components; GBM, generalized boosted regression modeling; StepCox, stepwise Cox; K-M, Kaplan-Meier; ROC, receiver operator curve; CTLEGS, cytotoxic T lymphocyte evasion gene signature; AHHXMC, Affiliated Haikou Hospital of Xiangya Medical College; ssGSEA, single cell gene set enrichment analysis; TIMER, Tumor Immune Estimation Resource; EPIC, Estimating the Proportion of Immune and Cancer cells; MCPcounter, Microenvironment Cell Populations-counter; TMB, tumor mutation burden; TIDE, Tumor Immune Dysfunction and Exclusion.

**Figure 2:**
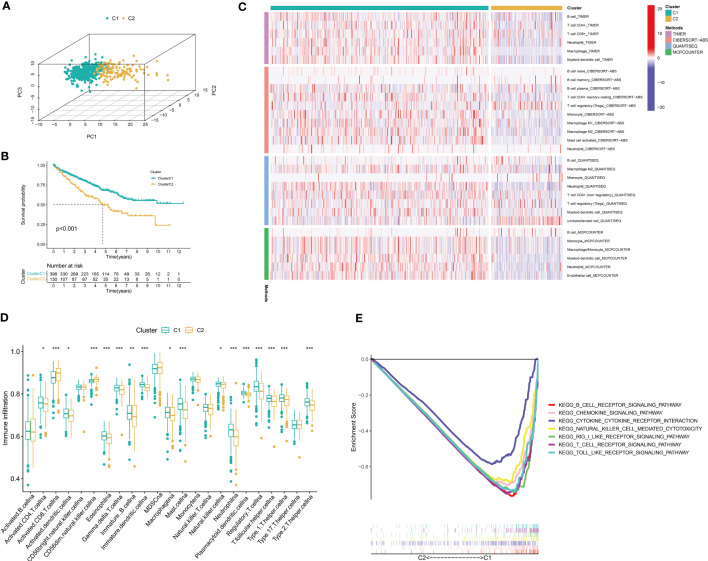
Identification of key CTLEGs. **(A)** Three-dimensional PCA analysis of gene cluster. **(B)** K-M survival analysis between cluster 1 and cluster 2. **(C)** Immune cell infiltration between different subtypes based on TIMER, CIBERSORT-ABS, QUANTISEQ, and MCPcounter algorithms. **(D)** The immune cell infiltration among different clusters was analyzed by the ssGSEA algorithm. **(E)** GSEA between different subtypes. CTLEGs, cytotoxic T lymphocyte evasion genes; PCA, principal component analysis; MCPcounter, Microenvironment Cell Populations-counter; ssGSEA, single cell gene set enrichment analysis; GSEA, gene set enrichment analysis. * Means *P* < 0.05; ** Means *P* < 0.01; *** Means *P* < 0.001.

**Figure 1** is the flow chart of the entire study. In the TCGA-KIRC cohort, 123 DEGs were identified. Compared with normal tissues, 28 genes were lower expressed in cancer tissues and 95 genes were higher expressed in cancer tissues (**Supplementary Table 4**). Then, 66 prognostic DEGs were screened through univariate Cox regression analysis (**Supplementary Table 5**; **Supplementary Figure 1A**). According to the expression value of key CTLEGs, the patients were divided into two gene clusters (**Supplementary Figure 1B**). PCA analysis found that cluster 1 and cluster 2 patients can be well separated (**Figure 2A**). Cluster 1 has a better prognosis than cluster 2 (**Figure 2B**). We found that cluster 1 has more immune cell infiltration than cluster 2 through the TIMER, CIBERSORT-ABS, QUANTISEQ, and MCPcounter algorithms (**Figure 2C**). ssGSEA analysis further confirmed that cluster 1 has more immune cell infiltration than cluster 2 (**Figure 2D**). GSEA indicated that immune-related pathways are mainly enriched in C1 (**Figure 2E**).

### Construction and validation of CTLEGS

**Figure 3:**
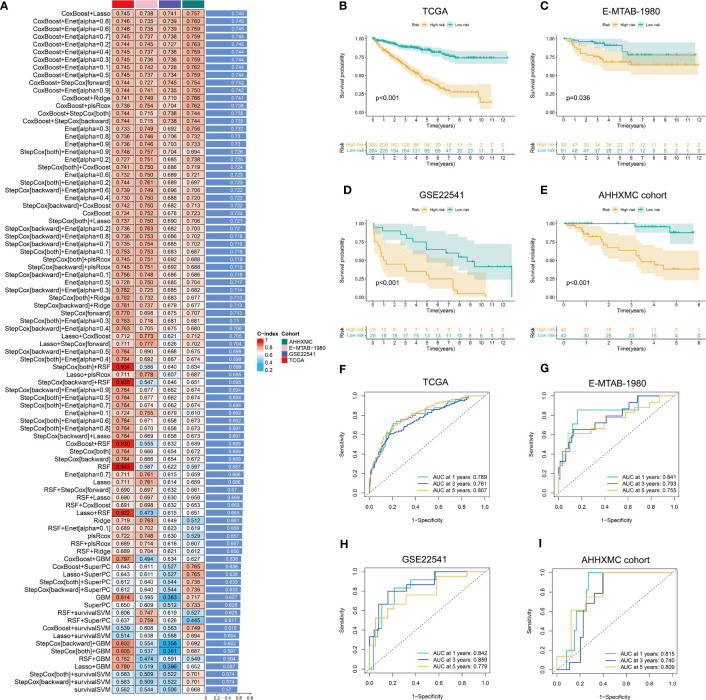
Construction and validation of CTLEGS. **(A)** C-indexs in different cohorts based on 10 machine learning algorithms. **(B–E)** K-M survival curves in different cohorts. **(F–I)** The ROC curves predict the performance of the signature in different cohorts. CTLEGS, cytotoxic T lymphocyte evasion gene signature; K-M, Kaplan-Meier; ROC, receiver operator curve; TCGA, The Cancer Genome Atlas; AHHXMC, Affiliated Haikou Hospital of Xiangya Medical College.

TCGA-KIRC cohort was used as the training set, whereas E-MTAB-1980, GSE22541, and AHHXMC cohorts were used as the testing sets. Under the cross-validation framework, 89 combinations of the 10 algorithms were used to screen the signature with the highest C-index. We found that the combination of CoxBoost+Lasso has the highest C-index (0.745) (**Figure 3A**; **Supplementary Table 6**). Moreover, CTLEGS included 18 CTLEGs (ATG10, FITM2, JAK1, RBCK1, STAT2, ATG5, RBM15, RNF31, VPS29, ATP13A1, BCL2L1, CEP55, CREBBP, DICER1, EMC2, IFNAR1, PCED1B, and SETDB1). A risk score was calculated for each patient based on the combination of CoxBoost+Lasso algorithms. On the basis of the median value of the risk score, we divided the patients into high- and low-risk groups. The prognosis of patients in the high-risk group is worse than that of patients in the low-risk group (**Figures 3B–E**). In TCGA, E-MTAB-1980, GSE22541, and AHHXMC cohorts, the areas under the curve (AUCs) are all larger than 0.7 (**Figures 3F–I**), indicating that our signature has good predictive efficiency.

### Comparison of CTLES and other signatures in KIRC

**Figure 4:**
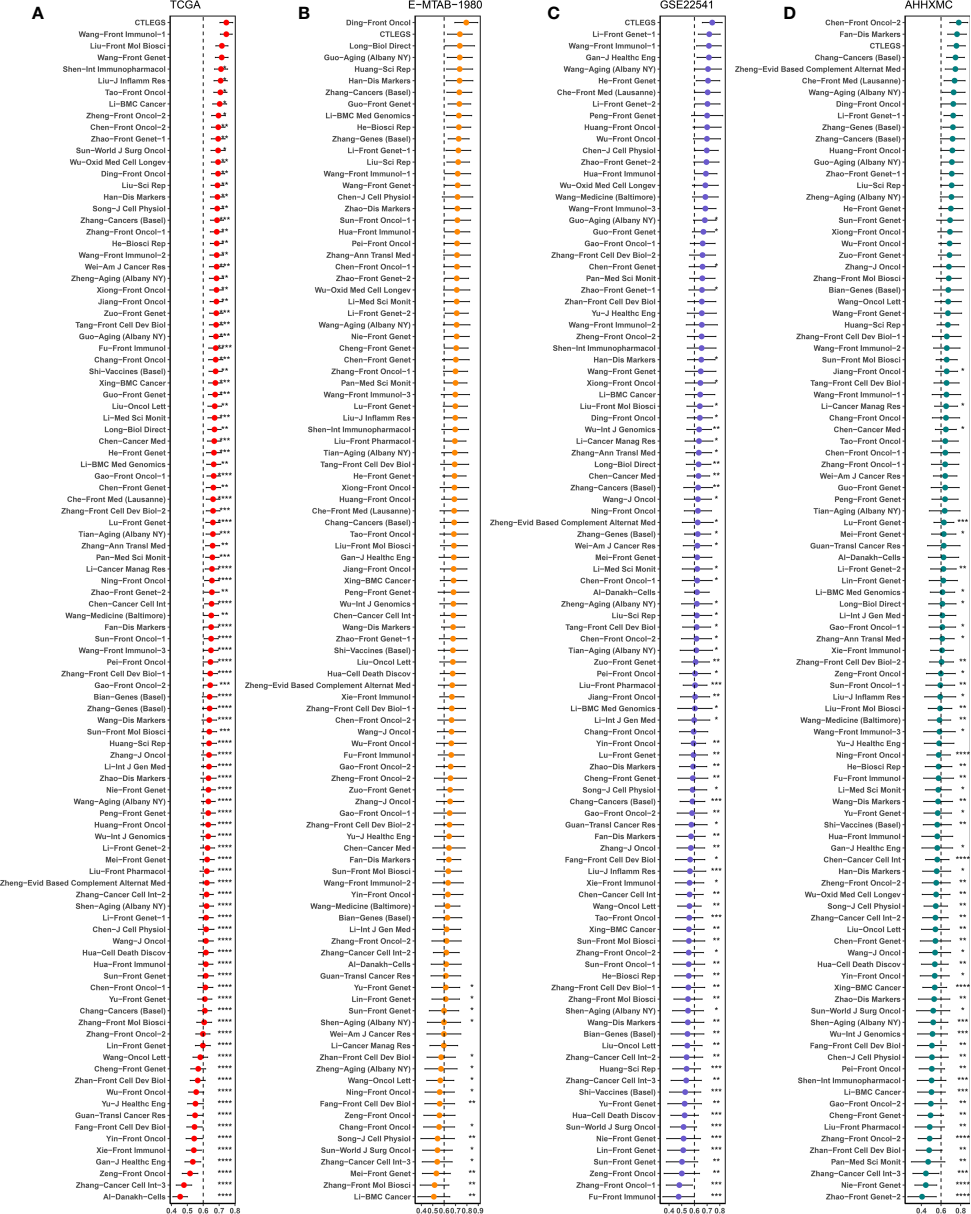
Comparisons of prognostic signatures in KIRC. **(A–D)** Comparison of C-index of signatures in TCGA, E-MTAB-1980, GSE22541, and AHHXMC cohorts. KIRC, kidney renal clear cell carcinoma; TCGA, The Cancer Genome Atlas; AHHXMC, Affiliated Haikou Hospital of Xiangya Medical College. * Means *P* < 0.05; ** Means *P* < 0.01; *** Means *P* < 0.001; **** Means *P* < 0.0001.

In recent years, many signatures have been developed in KIRC. However, the prediction efficiency is not significant. We collected 101 published signatures (**Supplementary Table 7**) involving various biological characteristics, including cuproptosis, ferroptosis, autophagy, pyroptosis, necroptosis, fatty acid metabolism, glutamine metabolism, immune checkpoint, Tumor-infiltrated CD8^+^ T Cell, IFN-γ response, oxidative stress, ERBB signaling pathway, basement membrane, and alternative splicing. In TCGA, E-MTAB-1980, GSE22541, and AHHXMC cohorts, we developed CTLEGS with good prediction performance in almost all models (**Figure 4**).

### Correlation analysis between signature and clinical variables

**Figure 5:**
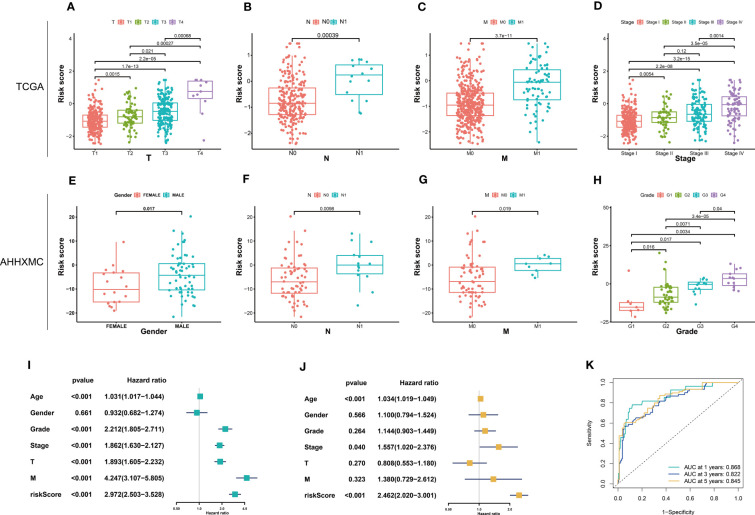
Correlation analysis between signature and clinical variables. **(A–D)** Risk scores in different TNM stages in the TCGA cohort. **(E–H)** Risk score in gender, N, M, and grade in the AHHXMC cohort. **(I–J)** Univariate and multivariate analyses of the predictive value of risk score and clinical variables in KIRC. **(K)** The AUCs of the nomogram. TNM, tumor, lymph node, metastases; TCGA, The Cancer Genome Atlas; AHHXMC, Affiliated Haikou Hospital of Xiangya Medical College; KIRC, kidney renal clear cell carcinoma; AUCs, areas under the curves.

In the TCGA cohort, the higher the TNM stage of the patients is, the higher the risk score is (**Figures 5A–D**). In the AHHXMC cohort, the risk score of males is higher than that of females (**Figure 5E**). The higher the N, M, and grade are, the higher the risk score is (**Figures 5F–H**). These results indicated that CTLEGS was positively correlated with the malignant degree of patients. Univariate and multivariate Cox regression analyses indicated that age, stage, and CTLES were independent risk predictors of patients (**Figures 5I, J**). In order to improve clinical application, we combined risk with common clinical features to construct a nomogram (**Supplementary Figure 2**). The AUCs of 1, 3, and 5 years reached 0.868, 0.822, and 0.845, respectively (**Figure 5K**). The nomogram can predict the prognosis of patients and further improve the predictive performance of CTLEGS.

### Pan-cancer analysis of the signature

**Figure 6:**
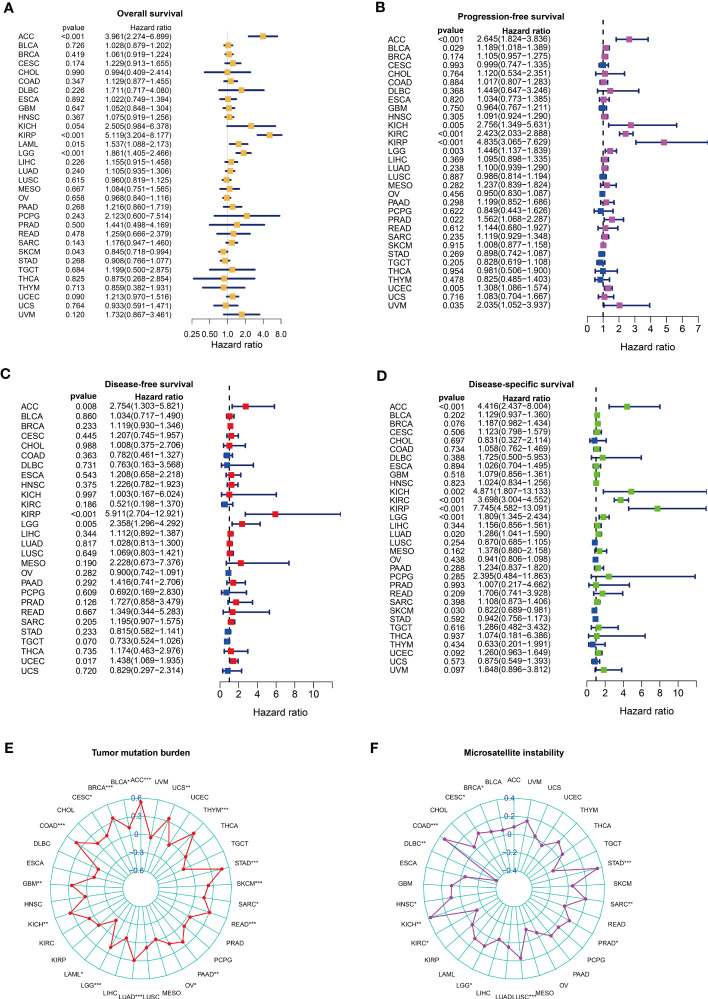
The predictive value of CTLEGS in pan-cancer prognosis. **(A-D)** The predictive performance of CTLEGS on the overall survival, PFS, DFS and DSS of pan-cancer. **(E, F)** Correlation analysis between CTLEGS and TMB and MSI in pan-cancer. CTLEGS, cytotoxic T lymphocyte evasion gene signature; DFS: disease-free survival; DSS: disease-specific survival; PFS: progression-free survival; TMB, tumor mutation burden; MSI, microsatellite instability. * Means *P* < 0.05; ** Means *P* < 0.01; *** Means *P* < 0.001.

We analyzed the predictive performance of the CTLES through pan-cancer analysis to test the universality and validity of the signature. Univariate Cox analysis indicated that the CTLES was still predictive of PFS and DSS of KIRC, which were risk factors for patients (**Figures 6A–D**). The CTLES still has good predictive power for other types of renal cell carcinoma, such as kidney chromophobe (KICH) and kidney renal papillary cell carcinoma (KIRP). In addition, we found that the CTLES has a good predictive performance on other types of tumors. It is also an unfavorable prognostic factor in most tumors, including OS, disease-free survival (DFS), disease-specific survival (DSS), and progression-free survival (PFS), such as adrenocortical carcinoma (ACC), acute myeloid leukemia, etc. K-M analysis indicated that the prognosis of patients in the high-risk group was worse than that of patients in the low-risk group (**Supplementary Figure 3**). With R>0.3 and *P*<0.05 as screening standards, the CTLES was strongly correlated with immunity in many cancers based on the estimate and CIBERSORT algorithms (**Supplementary Figures 4**; **5**). CTLEGS was correlated with the tumor mutation burden (TMB) of 18 types of tumors (**Figure 6E**). All were positively correlated except for acute myeloid leukemia. A strong correlation with microsatellite instability (MSI) existed in 12 types of tumors (**Figure 6F**). CTLEGS was negatively correlated with MSI in KIRC.

### Characteristics of immune cell infiltration and biological characteristics of the CTLEGS

**Figure 7:**
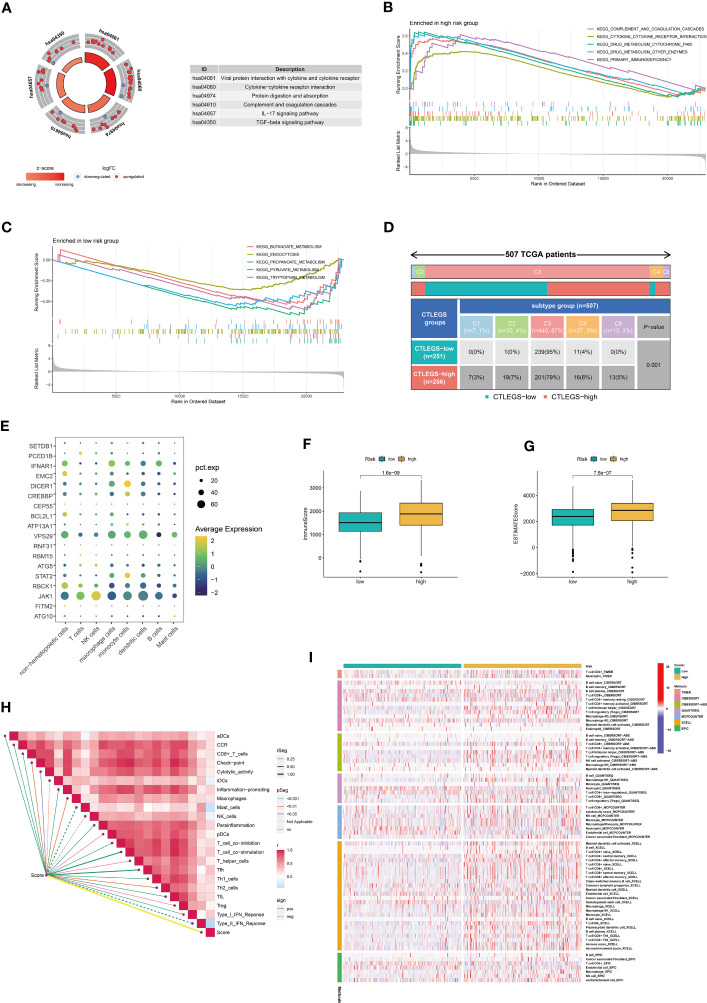
Characteristics of immune cell infiltration in high- and low-risk groups. **(A)** KEGG analyses of DEGs in different risk groups. **(B, C)** GSEA in different risk groups. **(D)** The relationship between risk score and immune subtypes. **(E)** The expression level of the key CTLEGs in different types of cells. **(F, G)** The difference between immune and estimate scores in the high-risk group based on the estimate algorithm. **(H)** The characteristics of immune cell infiltration in high- and low-risk groups were analyzed by the ssGSEA algorithm. **(I)** The characteristics of immune cell infiltration in high- and low-risk groups were analyzed with TIMER, CIBERSORT, CIBERSORT-ABS, QUANTISEQ, MCP counter, XCELL, and EPIC algorithms. KEGG, Kyoto Encyclopedia of Genes and Genomes; DEGs, differentially expressed genes; GSEA, gene set enrichment analysis; CTLEGs, cytotoxic T lymphocyte evasion genes; ssGSEA, single cell gene set enrichment analysis; TIMER, Tumor Immune Estimation Resource; MCPcounter, Microenvironment Cell Populations-counter. EPIC, Estimating the Proportion of Immune and Cancer cells; TCGA, The Cancer Genome Atlas; aDCs, activated dendritic cells; CCR, chemokine receptor; iDCs, immature dendritic cells; NK, natural killer; pDCs, plasmacytoid dendritic cells; Tfh, T follicular helper; Th1, T helper type 1; Th2, T helper type 2; TIL, tumor-infiltrating lymphocyte; IFN, interferon.

A total of 608 DEGs (**Supplementary Table 8**) between high- and low-risk groups were obtained with the “limma” package and performed GO and KEGG enrichment analyses. DEGs were mainly enriched in immune-related pathways, such as complex coagulation cascades, cytokine–cytokine receptor interaction, and interleukin (IL)-17 signaling pathway (**Figure 7A**; **Supplementary Figure 6A**). This finding suggests that CTLEGS was closely related to immunity. GSEA was further carried out. The results indicated that the immune- and drug-related pathways were mainly enriched in the high-risk group, such as additive and coagulation cascades, cytokine receptor interaction, drug metabolism, and cytochrome P450. Metabolism-related pathways were mainly enriched in the low-risk group, such as butanoate metabolism, propanoate metabolism, and pyruvate metabolism, etc. (**Figures 7B, C**). Interestingly, there were significant differences in immune type between the different groups (*P*=0.001, **Figure 7D**). We further analyzed the single-cell sequencing data set GSE207493 to study the expression level of CTLEGs in different cells. We integrated 19 KIRC samples and obtained 161327 cells. The cells were annotated as nonhematopoietic cells, T cells, NK cells, macrophage cells, monocyte cells, dendritic cells, B cells, and mast cells (**Supplementary Figure 6B**). Among them, JAK1 was highly expressed in all types of cells, and most of the CTLEGs were highly expressed in macrophages (**Figure 7E**; **Supplementary Figure 7**). The immune and estimate scores of the high-risk groups were higher than those of the low-risk groups according to the estimate algorithm (**Figures 7F, G**). ssGSEA indicated that risk score was positively correlated with most immune cells, such as aDCs, CD8^+^T cells, macrophages, T follicular helper (Tfh) cells, Th1 cells, Tfh2 cells, TIL, and Treg (**Figure 7H**). The risk score was positively correlated with immune checkpoint, cytolytic activity, and type 1 interferon response; but there was a negative correlation with type 2 interferon response (**Figure 7H**). On the basis of the seven algorithms of TIMER, CIBERSORT, CIBERSORT-ABS, QUANTISEQ, MCP counter, XCELL, and EPIC, we found that the high-risk group had more immune cell infiltration than the low-risk group (**Figure 7I**).

### Predictive value of the CTLEGS in immunotherapy response

**Figure 8:**
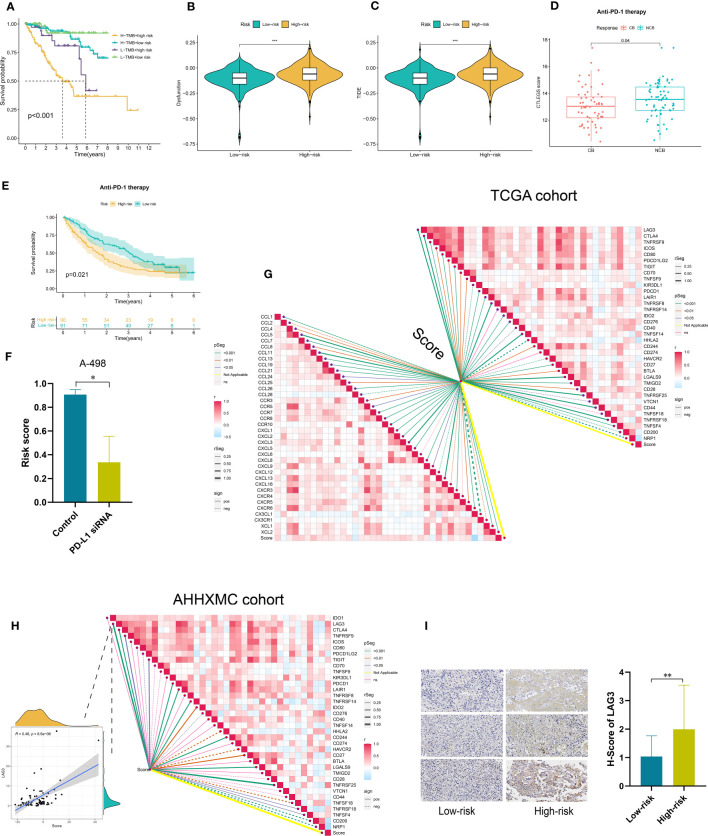
Predictive value of CTLEGS in immunotherapy response. **(A)** Survival differences among patients stratified by risk and TMB. **(B)** Dysfunction of high- and low-risk groups. **(C)** TIDE of high- and low-risk groups. **(D)** Risk scores between clinical benefit and no clinical benefit. **(E)** K-M survival curves of high- and low-risk groups in the CheckMate cohort. **(F)** Change in risk score after knocking down PD-L1. **(G)** Correlation analysis of the risk score with immune checkpoints and chemokines in the TCGA cohort. **(H)** Correlation analysis of the risk score with immune checkpoints in the AHHXMC cohort. **(I)** The difference in the protein expression levels of the LAG3 of different risk groups in the AHHXMC cohort. CTLEGS, cytotoxic T lymphocyte evasion gene signature; TMB, tumor mutation burden; TIDE, Tumor Immune Dysfunction and Exclusion; K-M, Kaplan-Meier; PD-L1, programmed death-ligand 1; TCGA, The Cancer Genome Atlas; AHHXMC, Affiliated Haikou Hospital of Xiangya Medical College; LAG3, lymphocyte-activation gene 3; TMB, tumor mutation burden; TIDE, Tumor Immune Dysfunction and Exclusion; PD-1, programmed cell death protein 1; CB, clinical benefit; NCB, no clinical benefit. ** Means *P* < 0.01; *** Means *P* < 0.001.

Immunotherapy has become increasingly crucial in KIRC. We further explored the predictive value of CTLEGS in immunotherapy response. The patients were stratified according to risk and TMB. Patients with a high risk and TMB had the worst prognosis (**Figure 8A**). The dysfunction in the high-risk group was higher than that in the low-risk group (**Figure 8B**). CTLEGs belong to immune evasion genes. We further analyzed the immune evasion of different risk groups. The Tumor Immune Dysfunction and Exclusion (TIDE) of the high-risk group was higher than that of the low-risk group (**Figure 8C**). In the CheckMate immunotherapy cohort, high-risk patients benefited slightly from immunotherapy, and their prognosis was worse than the prognosis of low-risk patients (**Figures 8D, E**). In the GSE199107 data set, the risk score was high in the IFN-γ treatment group (**Supplementary Figure 8**) and low in the PD-L1 knockdown group (**Figure 8F**). We found from the TCGA cohort that the risk score was positively correlated with most chemokines and immune checkpoints (**Figure 8G**). In the AHHXMC cohort, the risk score was also positively correlated with most immune checkpoints (**Figure 8H**). PDCD1, CTLA-4, TIGIT, LAG3, TNFRSF9, and CD27 are T cell depletion markers. The risk score was most correlated with the T cell degradation marker LAG3. As a new-generation immune checkpoint, LAG3 is expected to become a promising target in tumor immune therapy (38). Therefore, we further verified the correlation between the risk score and LAG3 at the protein level with IHC. We also found that the LAG3 protein level was higher in high-risk patients than in the low-risk patients (**Figure 8I**). These results indicated that the patients in the high-risk group presented immunosuppressive phenotype and slightly benefited from immunotherapy. CTLEGS possessed strong predictive ability in immunotherapy.

### Efficacy of CTLEGS in predicting drug sensitivity

**Figure 9:**
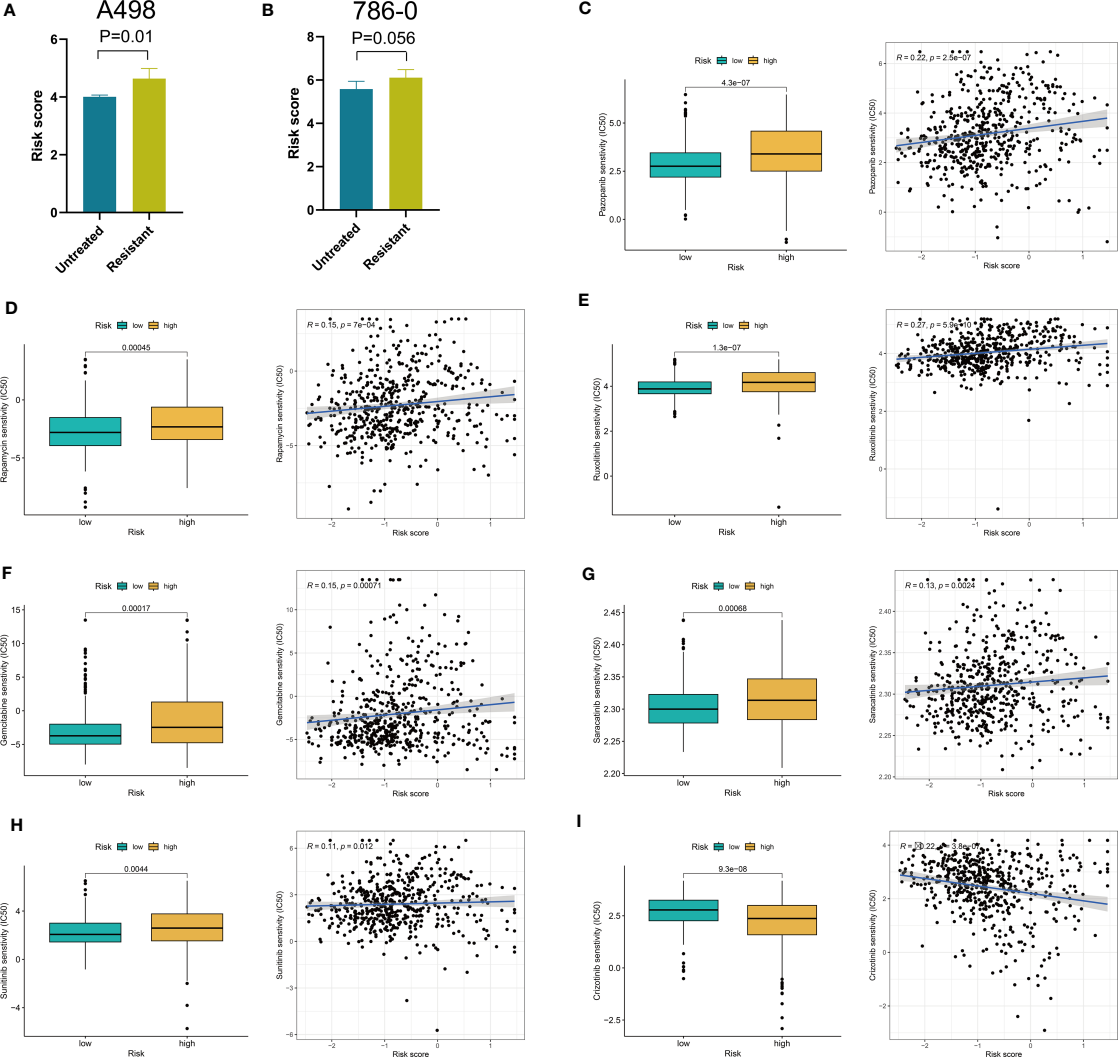
The analysis between risk score and drug sensitivity. **(A, B)** Risk score in sorafenib-resistant cell lines A498 and 786-0. **(C–I)** IC50 of pazopanib, rapamycin, ruxolitinib, gemcitabine, saracatinib, sunitinib, and crizotinib in different risk groups. IC50, half-maximal inhibitory concentration.

The risk score of sorafenib-resistant cell lines A498 and 786-0 was high, indicating that patients in the high-risk group were resistant to sorafenib (**Figures 9A, B**). The data from the Genomics of drug sensitivity in cancer (GDSC) database was analyzed with the “pRRophetic” package. The results indicated that the half maximal inhibitory concentration (IC50) values of pazopanib, rapamycin, ruxolitinib, gemcitabine, saracatinib, and sunitinib were high in the high-risk group, and the risk score was positively correlated with IC50 (**Figures 9C–H**). This finding indicated that the patients in the high-risk group were resistant to pazopanib, rapamycin, ruxolitinib, gemcitabine, saracatinib, and sunitinib. However, the IC50 of crizotinib in the high-risk group was low, indicating that the patients in the high-risk group were sensitive to crizotinib (**Figure 9I**). These results showed that CTLEGS could provide guidance for the personalized treatment of KIRC patients.

### Key genes related to CTLEGS were screened and verified *in vitro*

**Figure 10:**
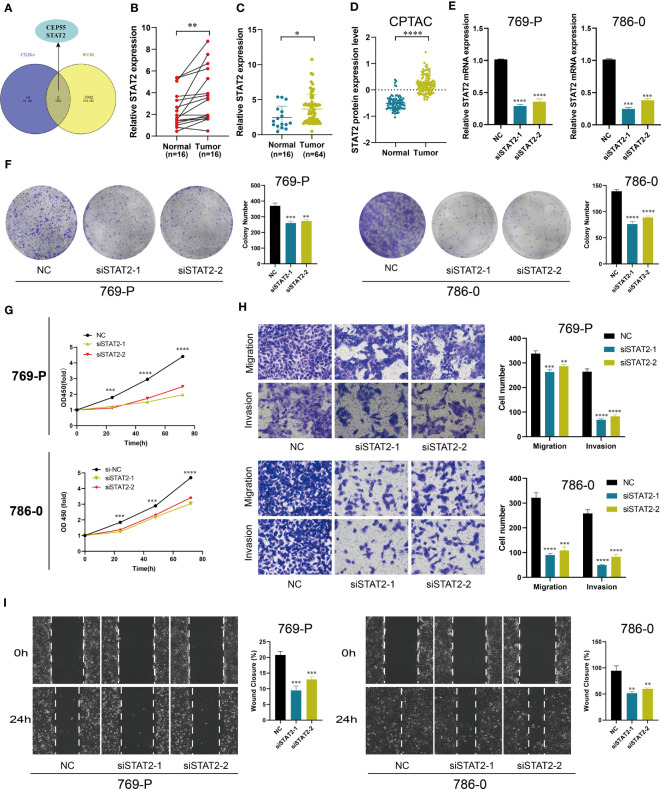
Cell function experiment of STAT2 in KIRC. **(A)** The Venn diagram of the key gene from WGCNA and CTLEGs from the signature. **(B, C)** The mRNA expression level of STAT2 in KIRC tissues. **(D)** The protein expression level of STAT2 in KIRC tissues. **(E)** The knockdown efficiency of STAT2 in KIRC cells. **(F, G)** The effect of STAT2 knockdown on cell proliferation was detected by colony formation and CCK8 assays. **(H)** Transwell was used to detect the knockdown effect of STAT2 on cell migration and invasion. **(I)** A wound-healing experiment was used to detect the knockdown effect of STAT2 on cell migration. STAT2, signal transducer and activator of transcription 2; KIRC, kidney renal clear cell carcinoma; WGCNA, weighted correlation network analysis; CTLEGs, cytotoxic T lymphocyte evasion genes; CCK8, Cell Counting Kit-8. * Means *P* < 0.05; ** Means *P* < 0.01; *** Means *P* < 0.001; **** Means *P* < 0.0001.

**Figure 11:**
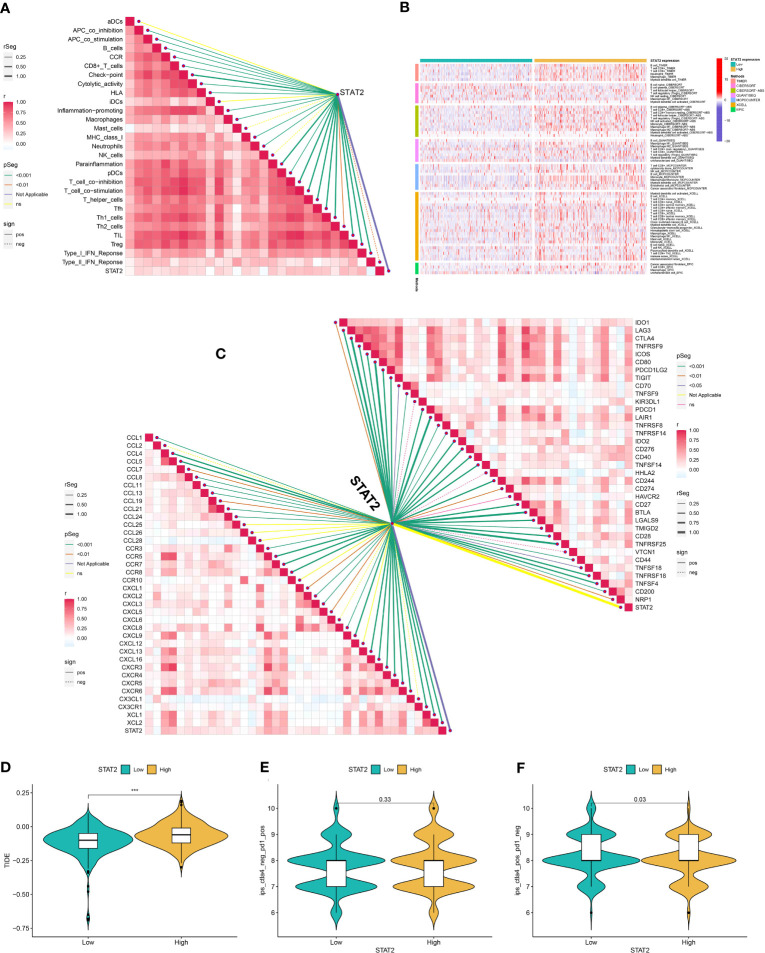
The role of STAT2 in immunity and immunotherapy. **(A)** Correlation analysis between STAT2 and immune cells was analyzed by the ssGSEA algorithm. **(B)** The characteristics of immune cell infiltration in STAT2 high- and low-expression groups were analyzed with TIMER, CIBERSORT, CIBERSORT-ABS, QUANTISEQ, MCP counter, XCELL, and EPIC algorithms. **(C)** Correlation analysis of STAT2 with immune checkpoints and chemokines in the TCGA cohort. **(D)** TIDE of STST2 high- and low-expression groups. **(E, F)** Differences in PD-1 and CTLA4 inhibitor responses between high and low STAT2 expression groups. STAT2, signal transducer and activator of transcription 2; ssGSEA, single cell gene set enrichment analysis; TIMER, Tumor Immune Estimation Resource; MCPcounter, Microenvironment Cell Populations-counter. EPIC, Estimating the Proportion of Immune and Cancer cells; TCGA, The Cancer Genome Atlas; TIDE, Tumor Immune Dysfunction and Exclusion; PD-1, programmed cell death protein 1; CTLA4, cytotoxic T-lymphocyte antigen-4; aDCs, activated dendritic cells; CCR, chemokine receptor; iDCs, immature dendritic cells; NK, natural killer; pDCs, plasmacytoid dendritic cells; Tfh, T follicular helper; Th1, T helper type 1; Th2, T helper type 2; TIL, tumor-infiltrating lymphocyte; IFN, interferon. *** Means *P* < 0.001.

WGCNA was used to identify the key genes closely related to CTLEGS. Power value 8 was selected for subsequent analysis, and a total of 25 modules were obtained (**Supplementary Figures 9A, B**). Magenta, dark orange, and cyan modules with correlation coefficients higher than 0.25 were chosen (**Supplementary Figure 9C**). The genes in the modules intersected with 18 CTLEGs in the signature (**Figure 10A**). CEP55 and STAT2 were identified. Chen et al. (39) proved that CEP55 can promote the malignant biological behavior of renal cancer cells. Thus, we selected STAT2 for *in vitro* verification. STAT2 expression was upregulated in the KIRC in both paired and unpaired tissues (**Figures 10B, C**). In the PDC000127 cohort, the protein expression level of STAT2 was higher in cancer tissue compared to normal tissue (**Figure 10D**). After transfection of 769-P and 786-0 cells with siSTAT2, the STAT2 mRNA expression level was significantly decreased (*P*<0.0001, **Figure 10E**). Colony formation and CCK8 assays showed that the cell proliferation ability was significantly reduced after the knockdown of STAT2 (**Figures 10F, G**). Transwell and wound-healing experiments showed that the migration ability of cells decreased after STAT2 knockdown, and the invasion ability weakened (**Figures 10H, I**). These results indicated that as a critical CTLEG for model construction, STAT2 plays a carcinogenic role in KIRC. In addition, we found that the immune cell infiltration characteristics of STAT2 were consistent with those of CTLEGS. According to ssGSEA, TIMER, CIBERSORT, CIBERSORT-ABS, QUANTISEQ, MCP counter, XCELL, and EPIC algorithms, the STAT2 high expression group showed more immune cell infiltration than low expression group (**Figures 11A, B**). STAT2 expression is positively correlated with most immune checkpoints and chemokines (**Figure 11C**). Compared with the STAT2 high expression group, the low expression group had lower TIDE (**Figure 11D**), which means that the possibility of immune evasion is less. PD-1 inhibitors showed no difference in STAT2 high and low expression groups (**Figure 11E**), but STAT2 low expression groups were sensitive to CTLA4 inhibitors (**Figure 11F**).

## Discussion

Renal cancer is a highly immunogenic tumor that mediates immune dysfunction through the infiltration of immunosuppressive cells into the microenvironment. ICI may benefit patients, but it may not be effective for all (40). Many patients develop drug resistance after receiving immunotherapy, mainly because of the heterogeneity of the tumor microenvironment (41, 42). Therefore, reliable biomarkers or signature for stratifying patients is urgently needed to help clinicians identify patients who may benefit from immunotherapy and guide patients to immunotherapy accurately.

In the present study, the patients were divided into clusters 1 and 2 according to 66 prognostic DEGs. Cluster 1 has a better prognosis than cluster 2. We used TCGA-KIRC as the training set and the E-MTAB-1980, GSE22541, and AHHXMC cohorts as the testing set. In the cross-validation framework, 89 combinations of Lasso, Ridge, Enet, StepCox, survival-SVM, CoxBoost, SuperPC, plsRcox, RSF, and GBM were used to select the signature with the highest C-index. CoxBoost+Lasso is the optimal model, and the C-index of each cohort is greater than 0.7. Combining different algorithms can reduce variable dimensions and make the signature simple and reliable. Compared to models constructed solely using a single algorithm (43, 44), our signature is more robust. The signature we constructed has high prediction accuracy in both public data sets and clinical in-house cohort. We collected 101 signatures in KIRC involving various biological pathways. These models are rarely applied in clinical practice and rarely verified by external data sets; alternatively, the validation performance of the external data sets is poor (45–48). The generalization and applicability of these signatures are poor. Our signature in each cohort has the best prediction performance in almost all models. We used one algorithm to select variables and another algorithm to construct the prognostic signature and obtain the optimal signature, which has good robustness and adaptability. The nomogram can provide personalized prognostic information to the patient and assist the clinician to develop an effective treatment plan for the patient (49, 50). In our study, the nomogram combined with clinical features could further improve the predictive ability of the signature. High risk is associated with high TNM and grade, thereby showing aggressive biological behavior. In addition, the signature was a poor prognostic factor for most tumors and closely related to the immunity of pan-cancer.

The immune microenvironment has become an important part of immunotherapy (51). Understanding the heterogeneity of the immune microenvironment plays a vital role in the precise immunotherapy of patients. Single-cell sequencing, based on single cell level analysis, can solve the problem of cell heterogeneity. As an important technical means of tumor microenvironment research, it is increasingly widely used (52, 53). In our study, single-cell sequencing indicated that most of the CTLEGs were highly expressed in macrophages, and JAK1 was highly expressed in all types of cells. Elevated levels of JAK1 protein are beneficial for the formation of immunosuppressive microenvironment in renal cell carcinoma (54). SETDB1 amplification in tumors is associated with immune rejection and immune checkpoint inhibitor resistance (55). Significant differences in the immune typing between the high- and low-risk groups existed. Immune-related pathways were mainly enriched in the high-risk group. The infiltration of immunosuppressive cells, such as CD8^+^T cells, Treg cells, macrophages, monocyte, plasmacytoid dendritic cells, and Tfh cells, in high-risk groups, was high according to various algorithms. Unlike other tumors, CD8^+^ T cells exhibited high infiltration in KIRC, which was associated with poor prognosis (56). The high infiltration of macrophages was related to the poor prognosis of KIRC (57). Research showed that targeted macrophages may become a promising treatment for patients (58). Treg cells can escape immune surveillance by expressing coinhibitory molecules CTLA4, PD1, LAG3, TIM3, and TIGIT (59). These results suggested that high-risk groups exhibited an immunosuppressive microenvironment. An immunosuppressive microenvironment is an important mechanism of tumor immune invasion. We also found that high-risk patients have higher TIDE. One mechanism of immune evasion is that the tumor is in a state of dysfunction despite the high CTLs infiltration; another mechanism is that immunosuppressive factors can remove T cells infiltrating the tumors. Therefore, Peng et al. (60) integrated two immune escape mechanisms to develop the TIDE score, which is more effective in predicting the response of patients to immunotherapy than PD-L1. The risk score is negatively correlated with type 2 interferon response, which is involved in antitumor immunity. These findings indicated poor antitumor immunity in high-risk patients.

Correlation analysis indicated that the risk score was positively correlated with most immune checkpoints and chemokines, among which PDCD1, CTLA-4, TIGIT, LAG3, TNFRSF9, and CD27 were T cell depletion markers. In the AHHXMC cohort, the correlation between LAG3 and risk score was the strongest in T cell depletion markers. LAG3, a coinhibitory receptor expressed in the activated CD4^+^ and CD8^+^T cells and in depleted CD8^+^T cells, has become one of the most promising and potential targets in cancer treatment (61–65). IHC further confirmed that the level of LAG3 protein was higher in high-risk patients than in low-risk patients. CXCL13 is a B-lymphocyte chemokine, and CXCL13^+^CD8^+^T cells are closely related to immune evasion and poor prognosis (66). The risk score increased after treatment with IFN-γ. IFN-mediated upregulation of immune checkpoints can promote immune evasion, which is related to the adverse reactions of renal cancer patients to ICB (67). In the CheckMate immunotherapy cohort, high-risk patients benefited slightly from immunotherapy. They also have a poor prognosis. Therefore, we suspected that the high-risk group benefitted slightly from immunotherapy because of immune evasion. In addition, the high-risk group was closely related to drug-related pathways. Drug sensitivity analysis indicated that the high-risk group was resistant to many drugs, such as pazopanib, rapamycin, ruxolitinib, gemcitabine, saracatinib, and sunitinib. These results showed that CTLEGS can provide precise treatment for patients.

WGCNA was used to identify the genes closely related to CTLEGS. These genes intersected with the 18 genes of the CTLEGS. STAT2 was selected for further research. STAT2 plays an important role in the immune response of the body, participating in the activation of immune cells and the production of inflammatory factors (68). STAT2 promotes tumor immune escape by upregulating PD-L1 expression (69). The JAK/STAT pathway involved by STAT2 can affect the tumor immune microenvironment (70). JAK/STAT pathway is also involved in the malignant progression of the tumor. GINS2 inhibits lung cancer progression by inhibiting the STAT signal pathway (71). TRIM66 promotes the malignant biological behavior of prostate cancer through JAK/STAT pathway (72). Consistent with these studies, we found that the high expression group of STAT2 is more likely to immune evasion. STAT2 may play a carcinogenic role in KIRC, and can be a potential therapeutic target for KIRC.

Our study still has some limitations. First, the predictive performance of CTLEGS should be validated in other immunotherapy cohorts. Second, the sample was investigated in a single-center retrospective study, which needs to be further verified in a prospective multicenter cohort in the future. Third, the role of STAT2 in the immune microenvironment of KIRC and the mechanism of immune evasion still need to be further studied.

## Conclusions

We used machine learning to develop a robust CTLEGS that reveals tumor microenvironment characteristics and effectively predicts immunotherapy response. Thus, this signature can provide guidance for precise therapy of KIRC. The key gene STAT2 played a carcinogenic role in KIRC.

## Data availability statement

The data presented in the study are deposited in the NCBI Sequence Read Archive, accession number PRJNA997383. Other datasets presented in this study can be found in online repositories. The names of the repository/repositories and accession number(s) can be found in the article/**Supplementary Material**.

## Ethics statement

The studies involving human participants were reviewed and approved by the ethics committee of Affiliated Haikou Hospital of Xiangya Medical College, Central South University. The patients/participants provided their written informed consent to participate in this study.

## Author contributions

MC designed the research. ZN and DH carried out the analyses. LZ, YG, and HC performed the experiments. SZ wrote the manuscript. All authors contributed to the article and approved the submitted version.

## Supplementary material

SUPPLEMENTARY FIGURE 1 CTLEGs clusters analyses in KIRC. **(A)** DEGs were screened through univariate Cox regression analysis. **(B)** The patients were divided into two gene clusters.

SUPPLEMENTARY FIGURE 2 Nomogram combined risk with common clinical features.

SUPPLEMENTARY FIGURE 3 M analyses in pan-cancer.

SUPPLEMENTARY FIGURE 4 Correlation analyses of CTLES and immunity in pan-cancer based on estimate algorithm.

SUPPLEMENTARY FIGURE 5 Correlation analyses of CTLES and immunity in pan-cancer based on CIBERSORT algorithm.

SUPPLEMENTARY FIGURE 6 Enrichment analyses of DEGs and UMAP plot of single cells. **(A)** Enrichment analyses of DEGs. **(B)** UMAP plot of single cells.

SUPPLEMENTARY FIGURE 7 Distribution and expression patterns of CTLEGs in single cells.

SUPPLEMENTARY FIGURE 8 Changes in risk score after interferon treatment.

SUPPLEMENTARY FIGURE 9 WGCNA was used to identify the key genes closely related to CTLEGS. **(A)** Screening of soft threshold. **(B)** Hierarchical clustering dendrogram of 25 modules. **(C)** Heatmap of correlations between modules and risk core.

SUPPLEMENTARY TABLE 2 182 CTLEGs from published literature.

SUPPLEMENTARY TABLE 8 608 DEGs between high- and low-risk groups.
