# Supplementary figures and images for: Machine learning-based on cytotoxic T lymphocyte evasion gene develops a novel signature to predict prognosis and immunotherapy responses for kidney renal clear cell carcinoma patients
Source: Front Immunol. 2023 Jul 31;14:1192428. doi: 10.3389/fimmu.2023.1192428 (PMC10436106; doi:10.3389/fimmu.2023.1192428)

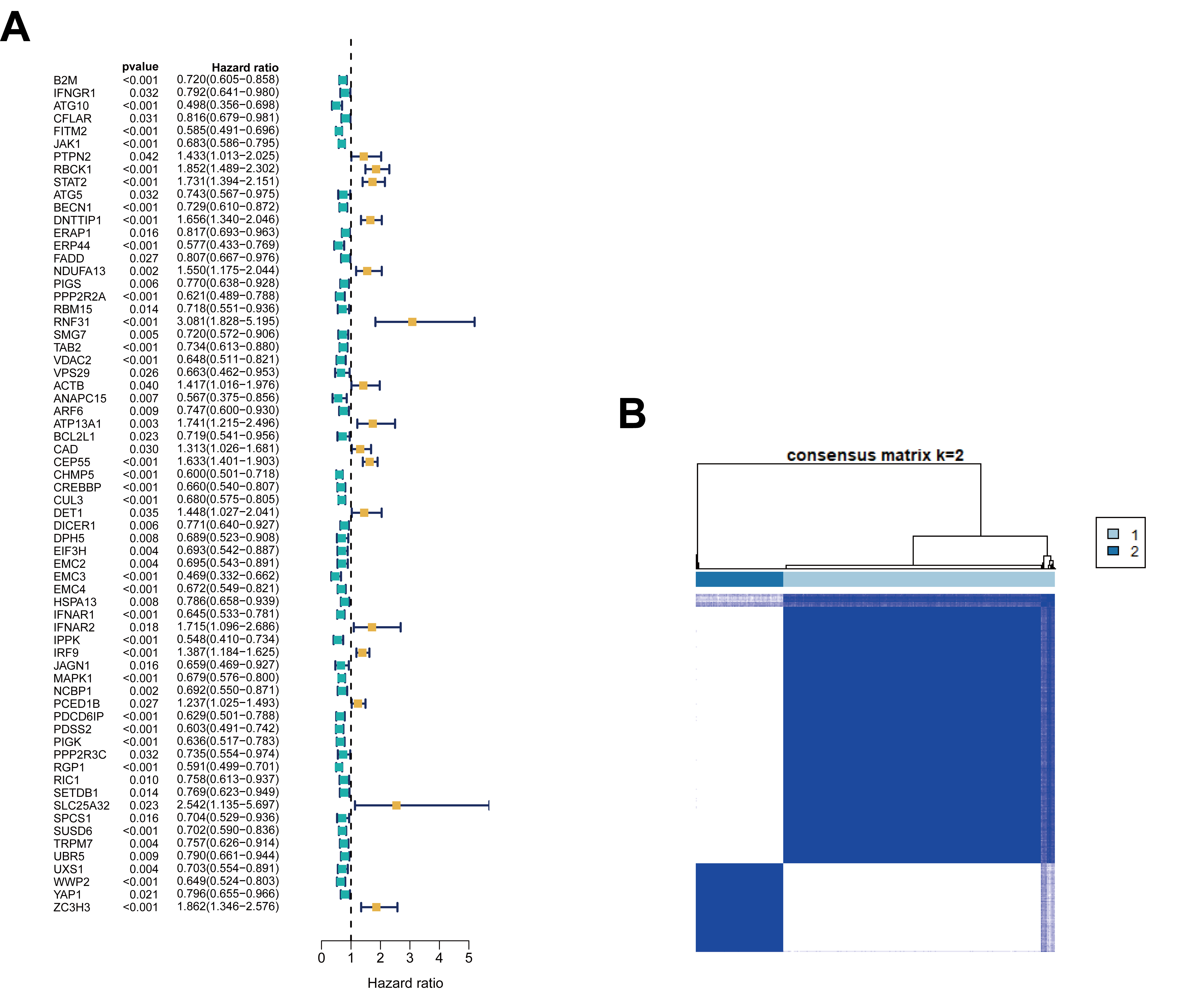

Supplement: SUPPLEMENTARY FIGURE 1 — CTLEGs clusters analyses in KIRC. (A) DEGs were screened through univariate Cox regression analysis. (B) The patients were divided into two gene clusters. [file Image_1.jpeg]

Points

N

Gender

T

M

Age\*\*

risk\*\*\*

Stage\*

**Total points**

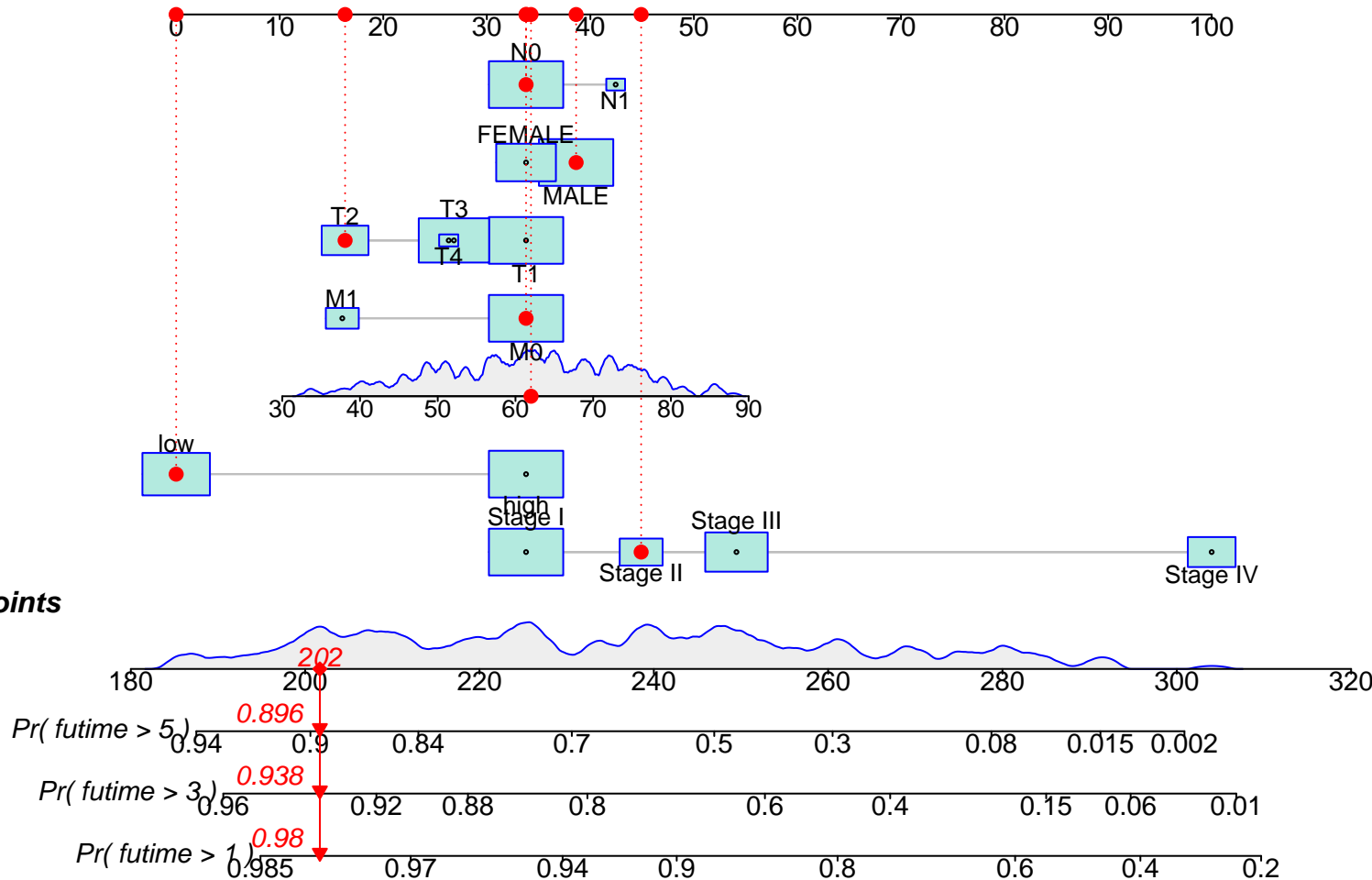

Supplement: SUPPLEMENTARY FIGURE 2 — Nomogram combined risk with common clinical features. [file Image_2.pdf]

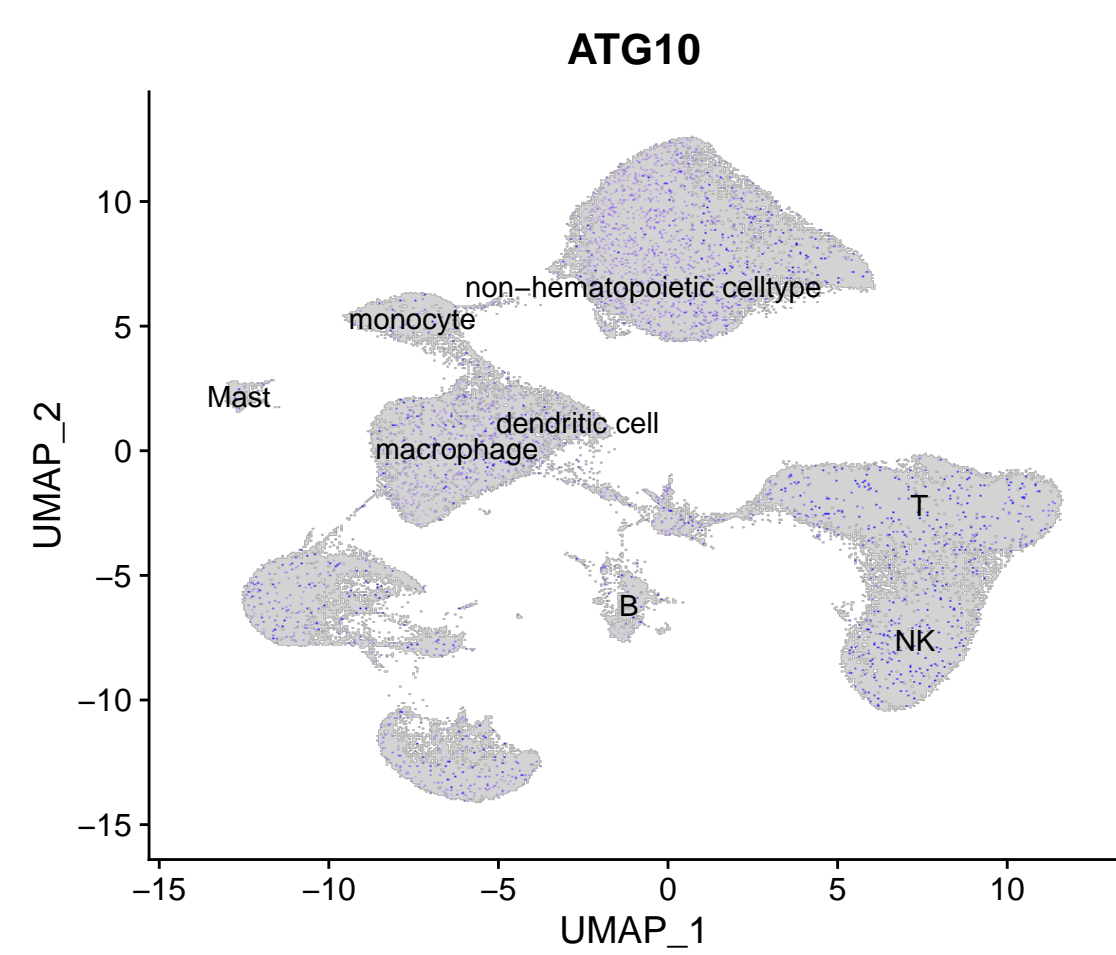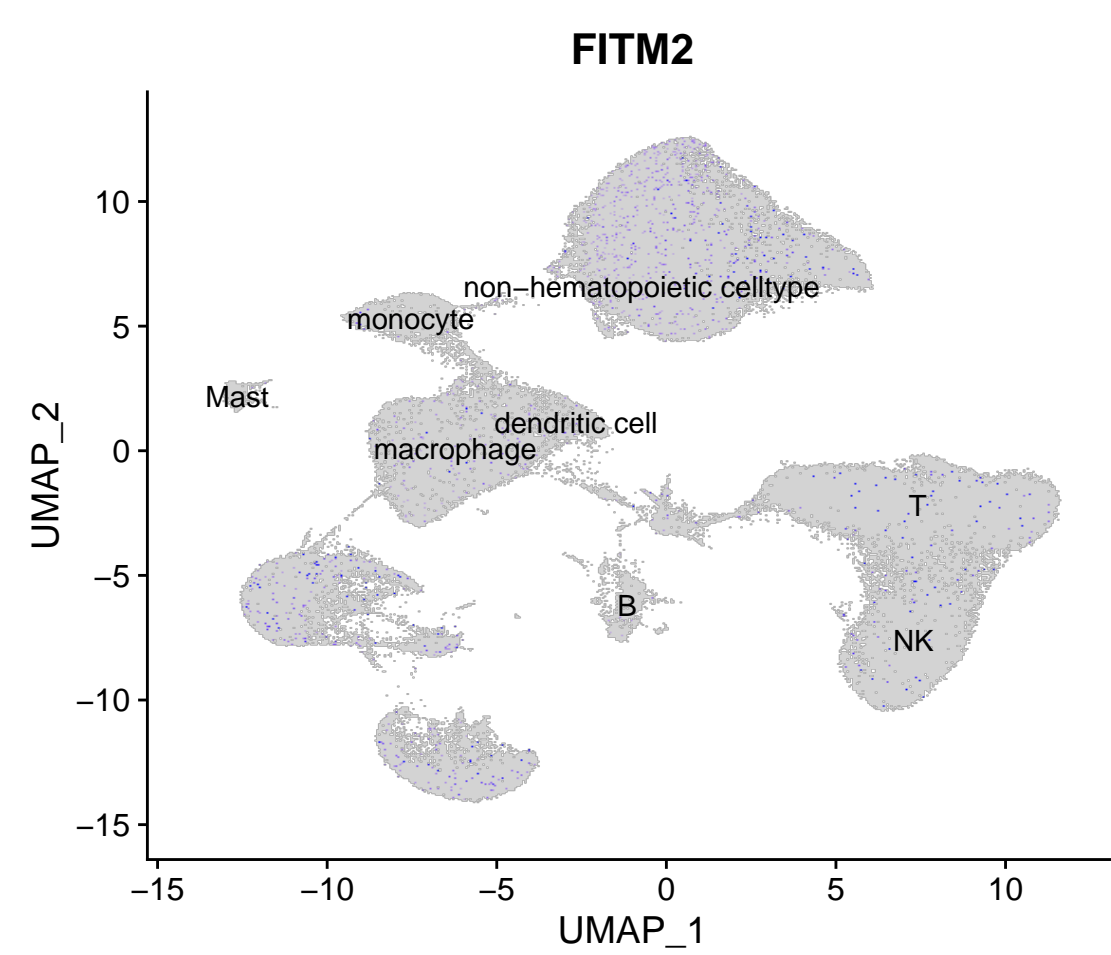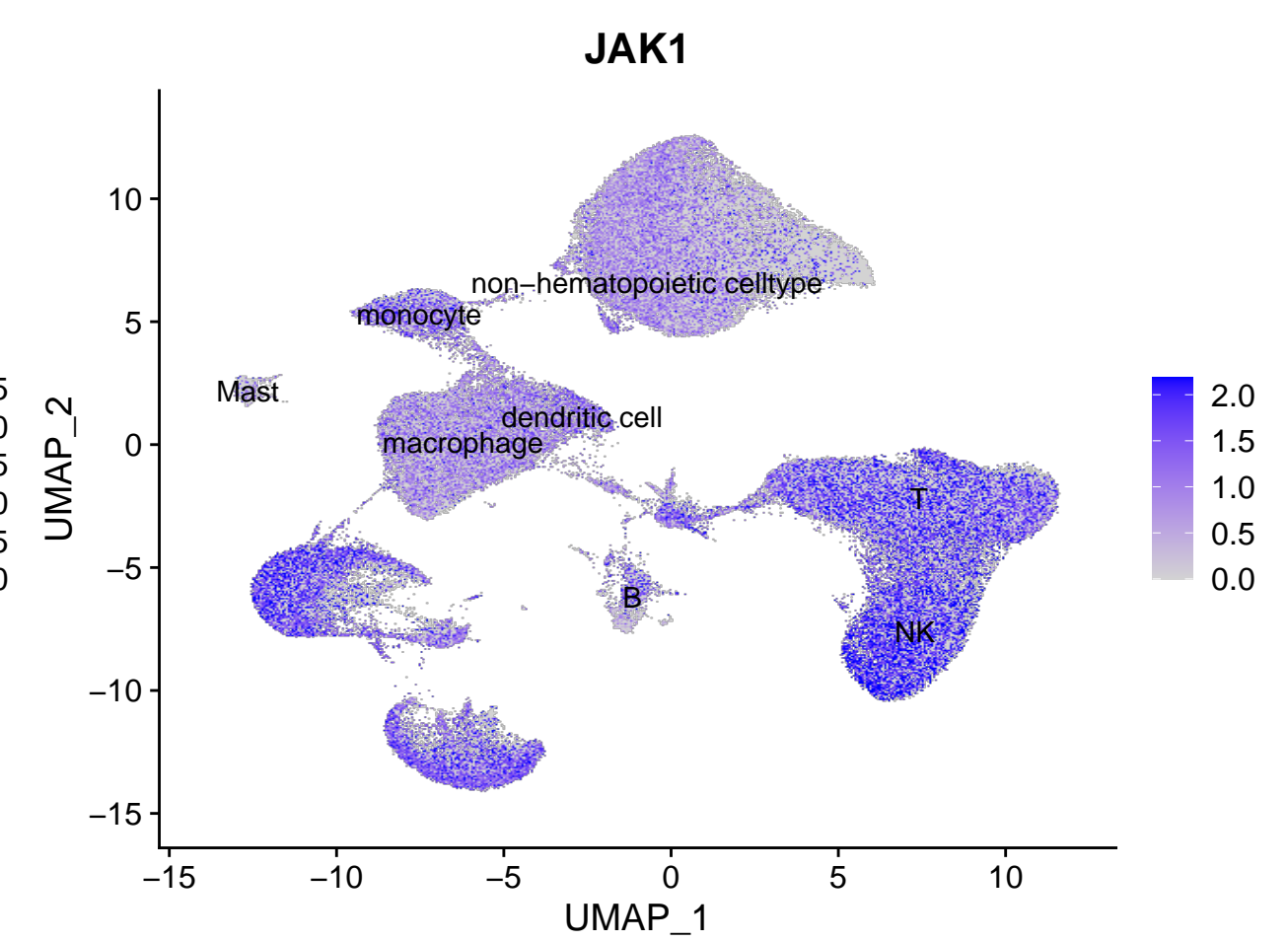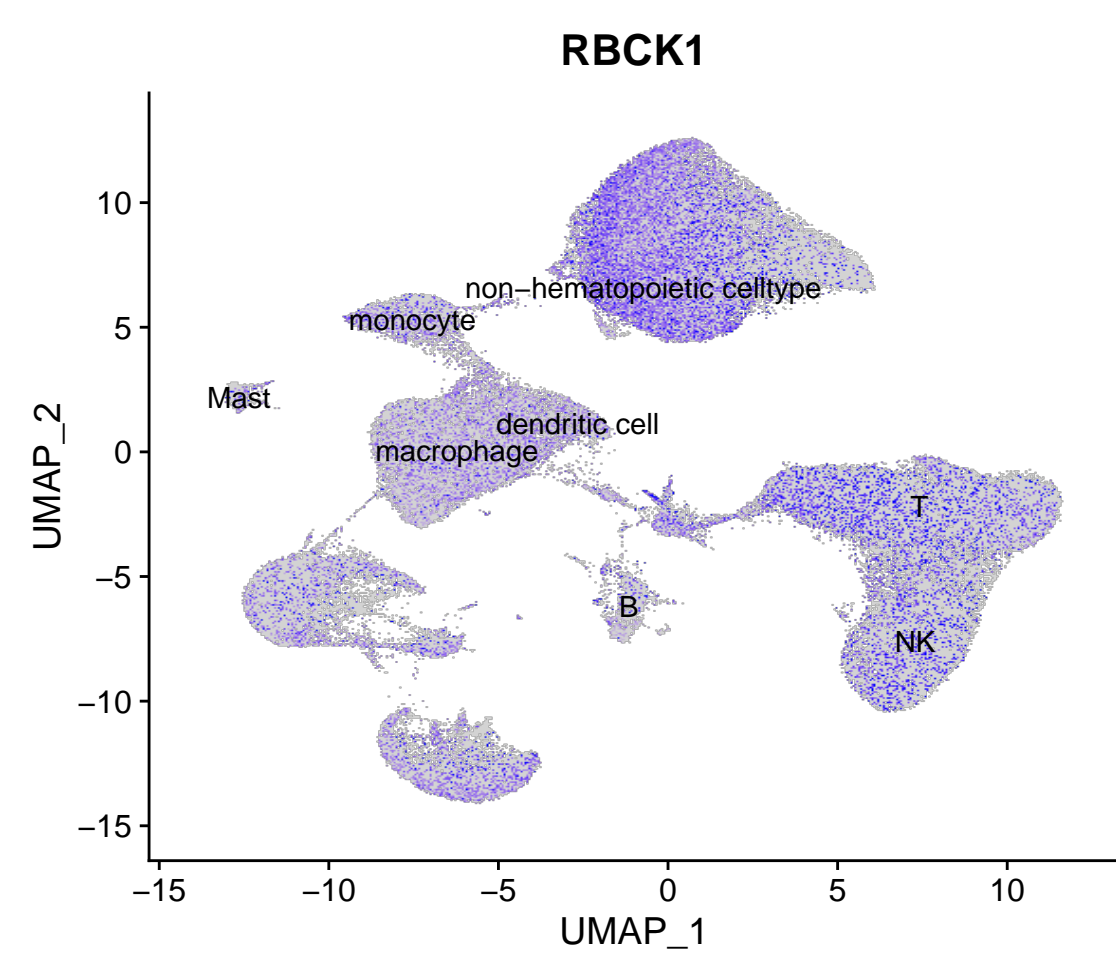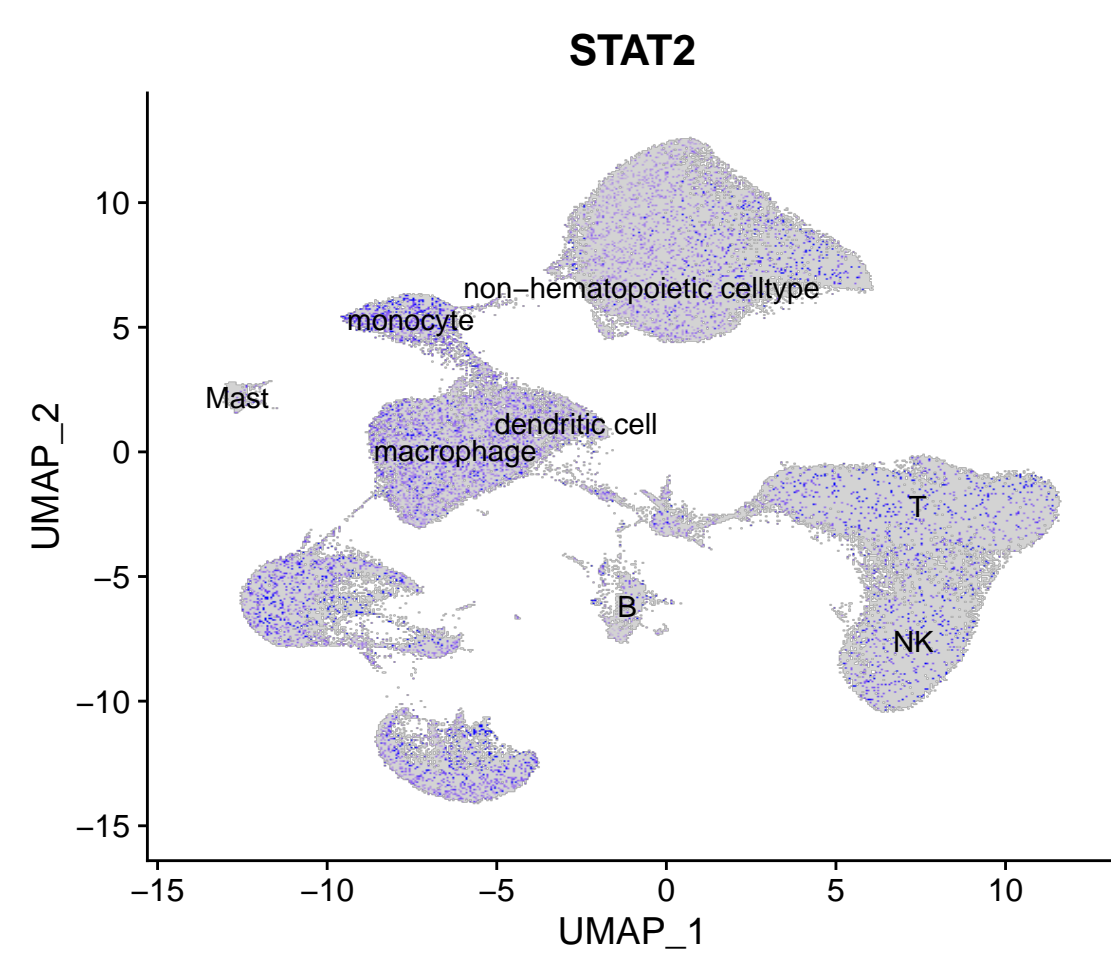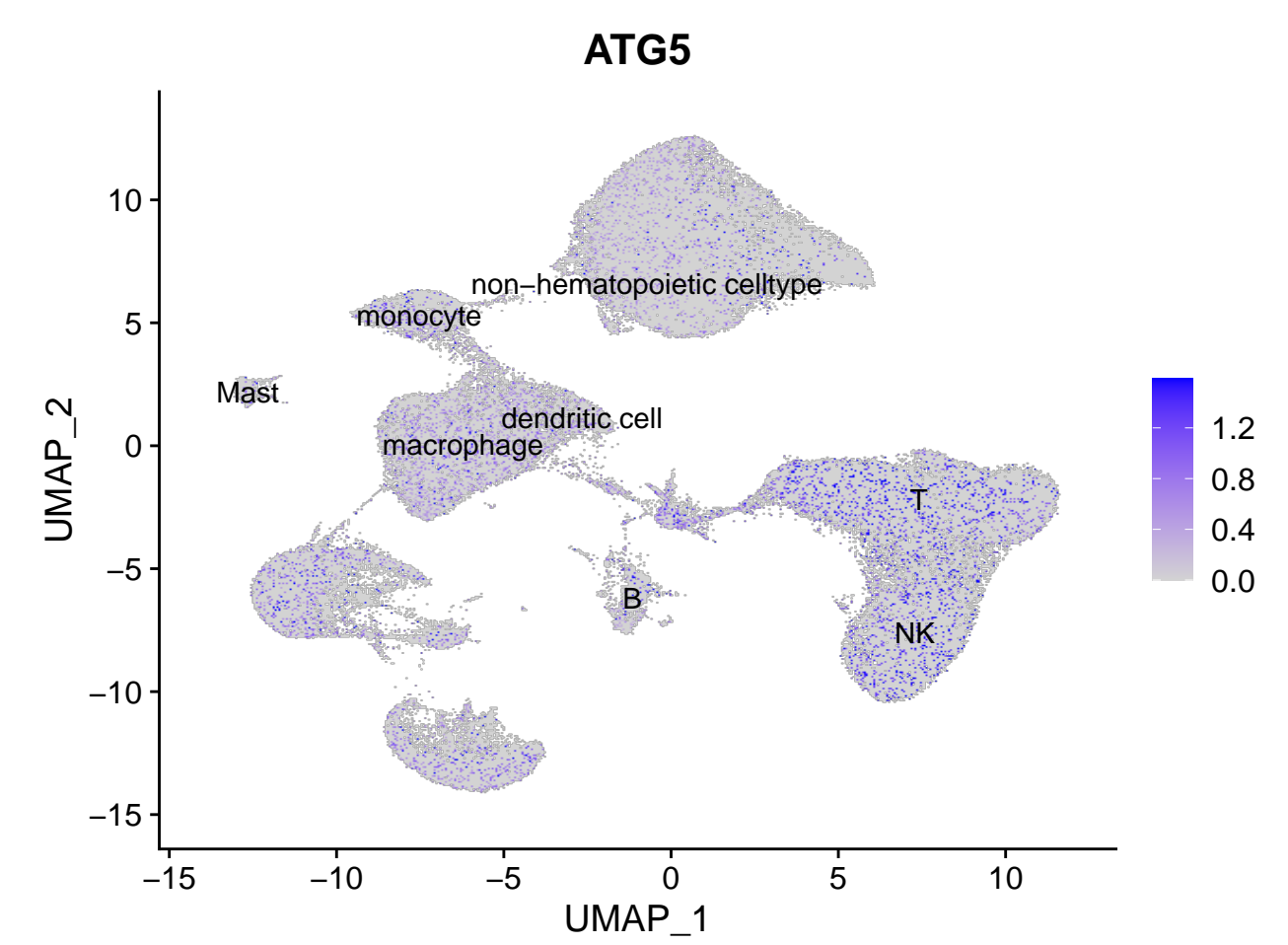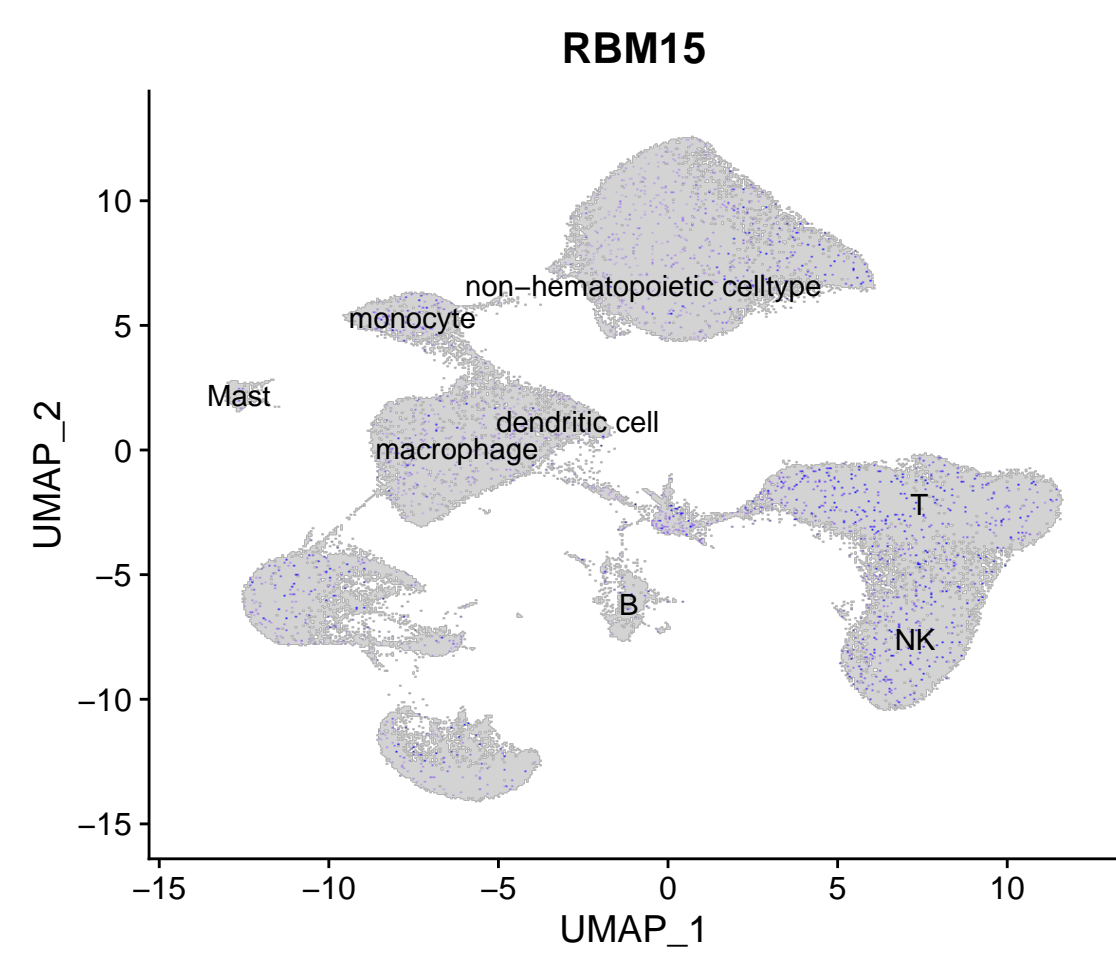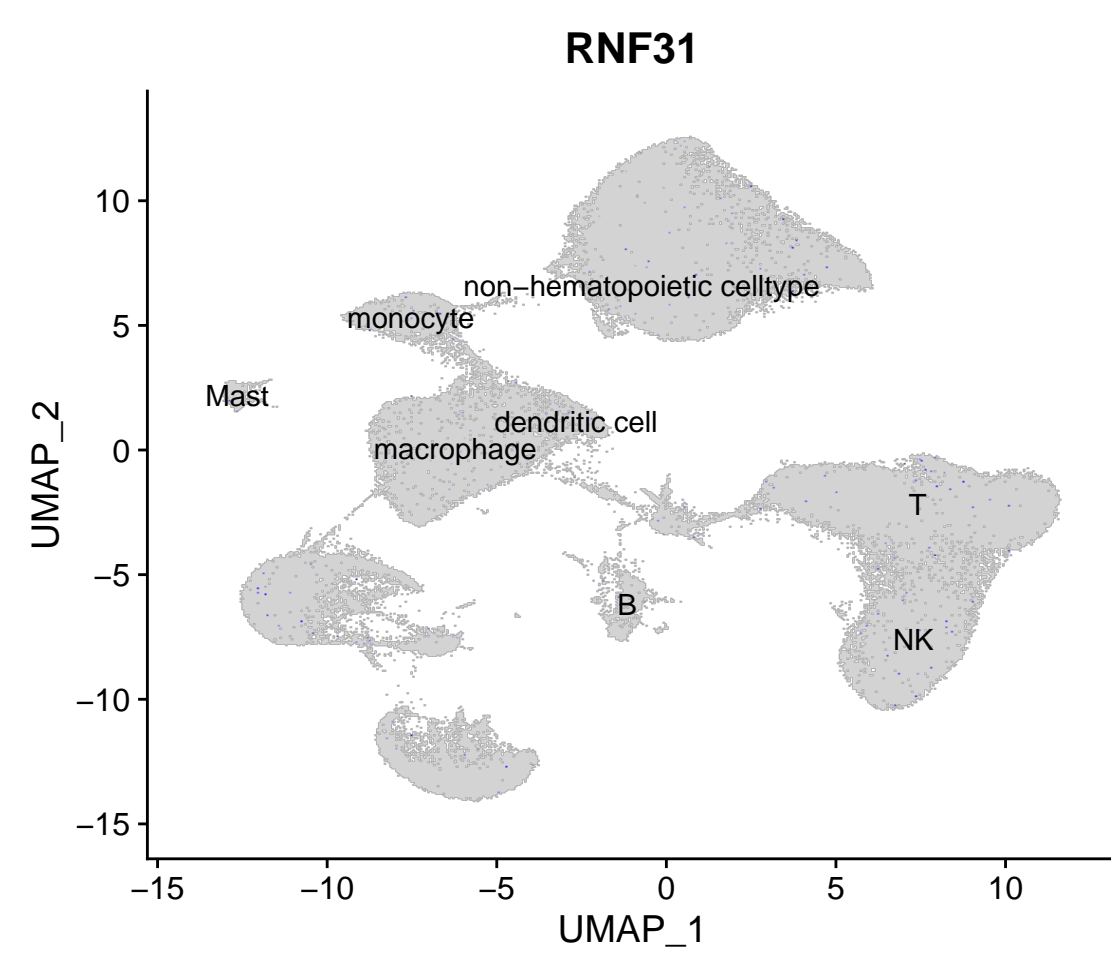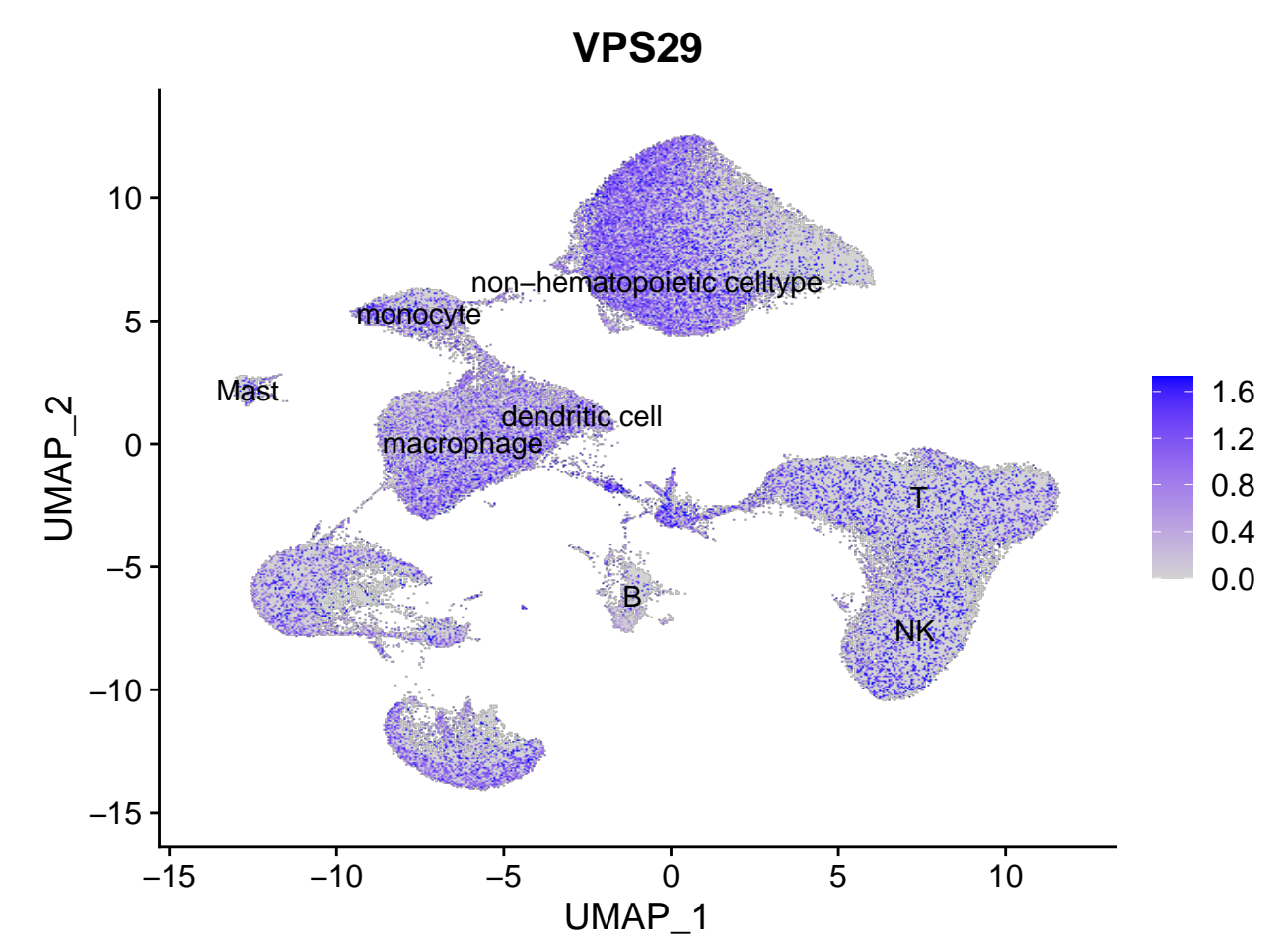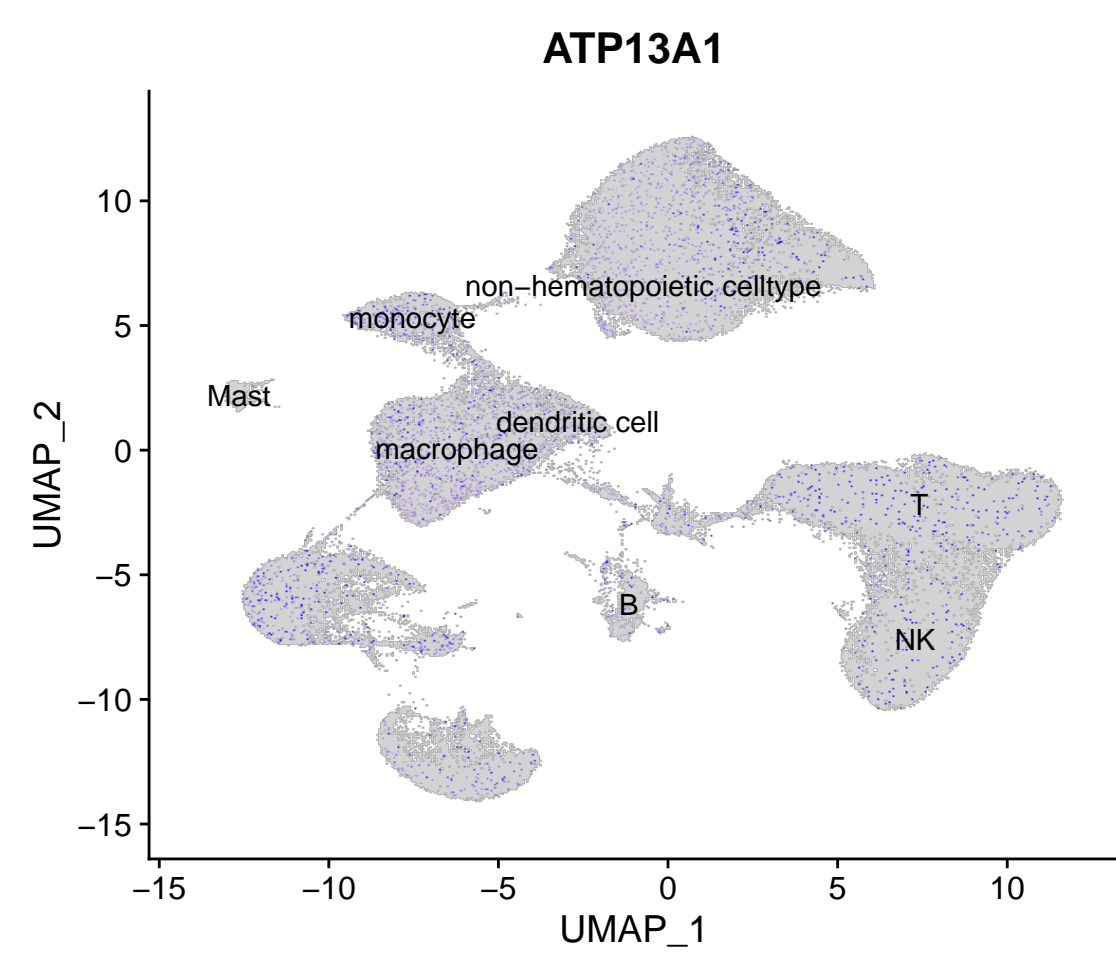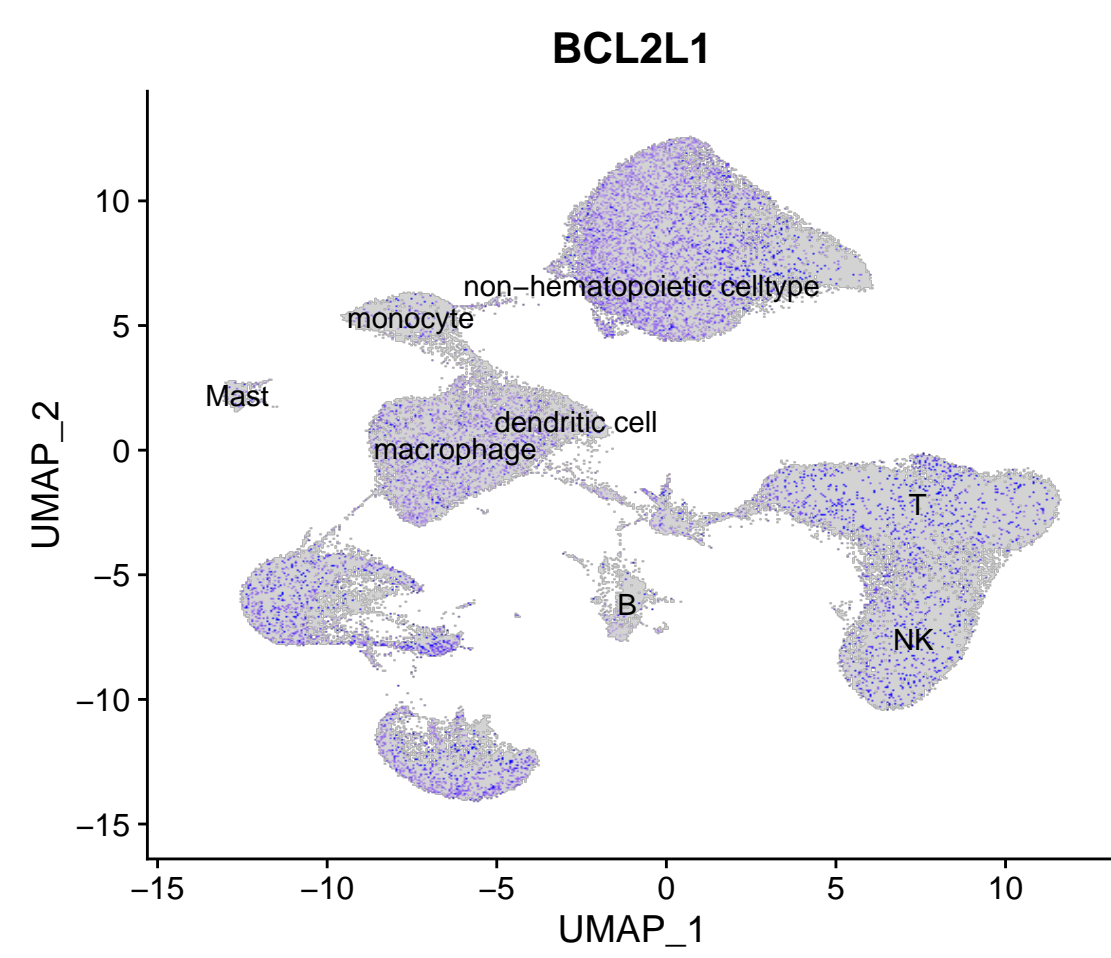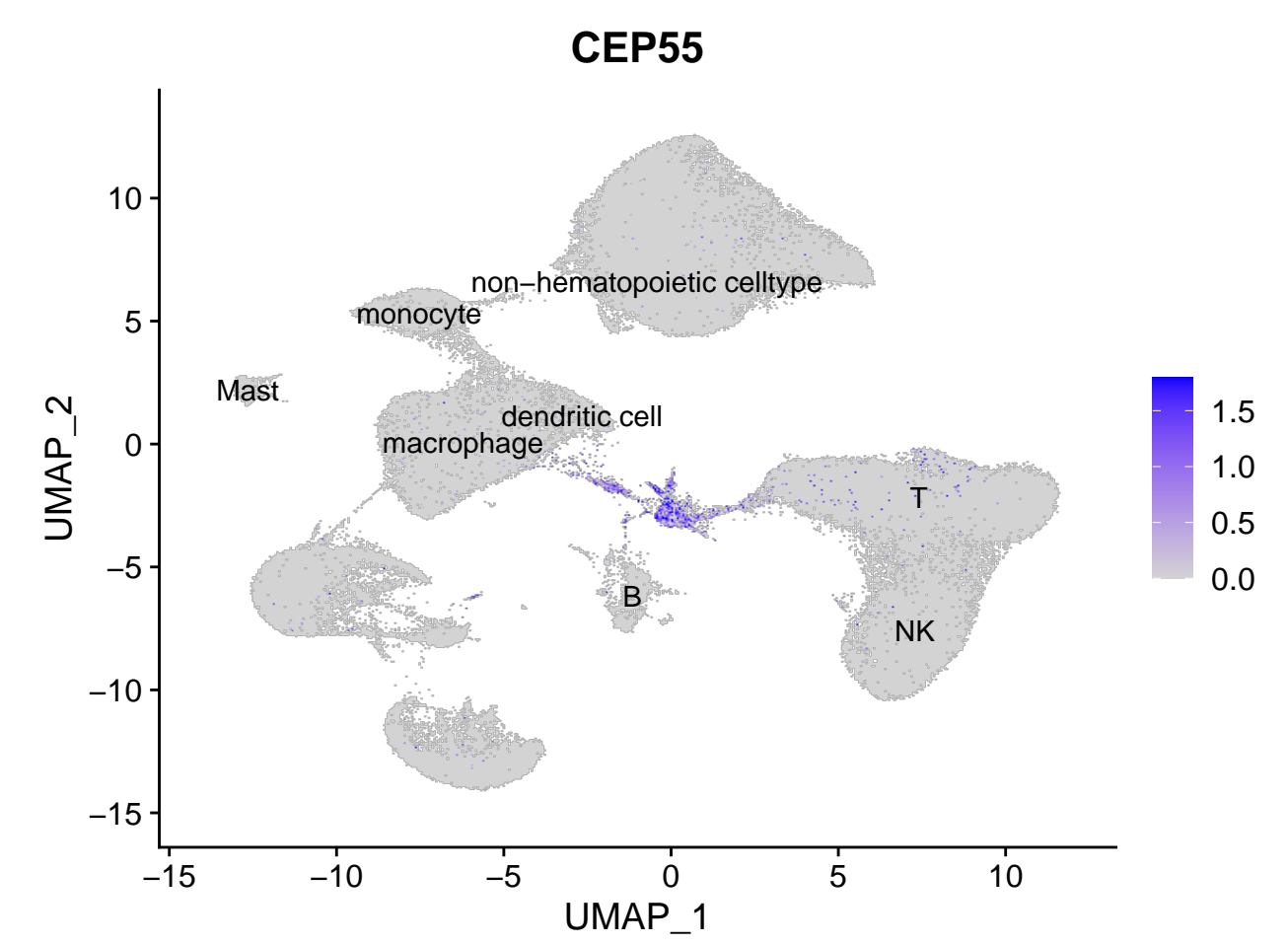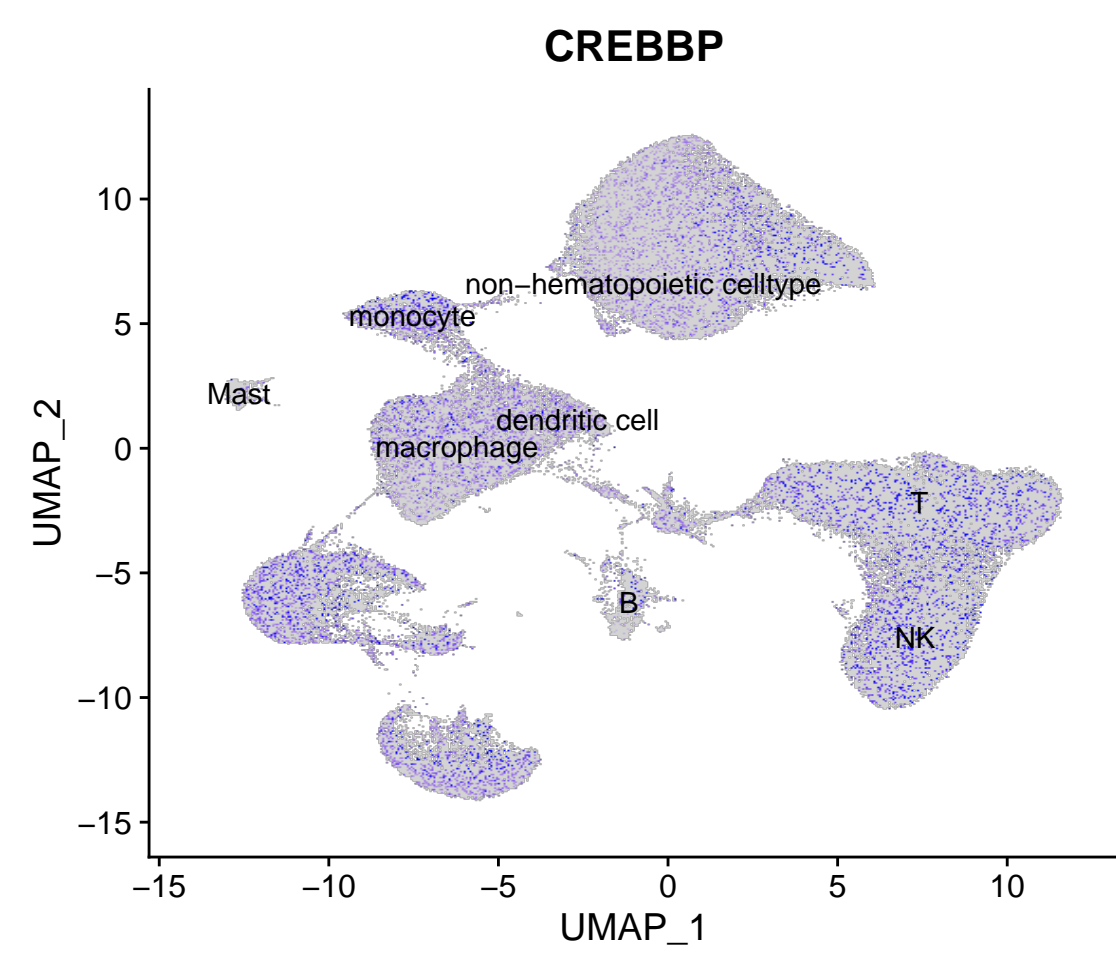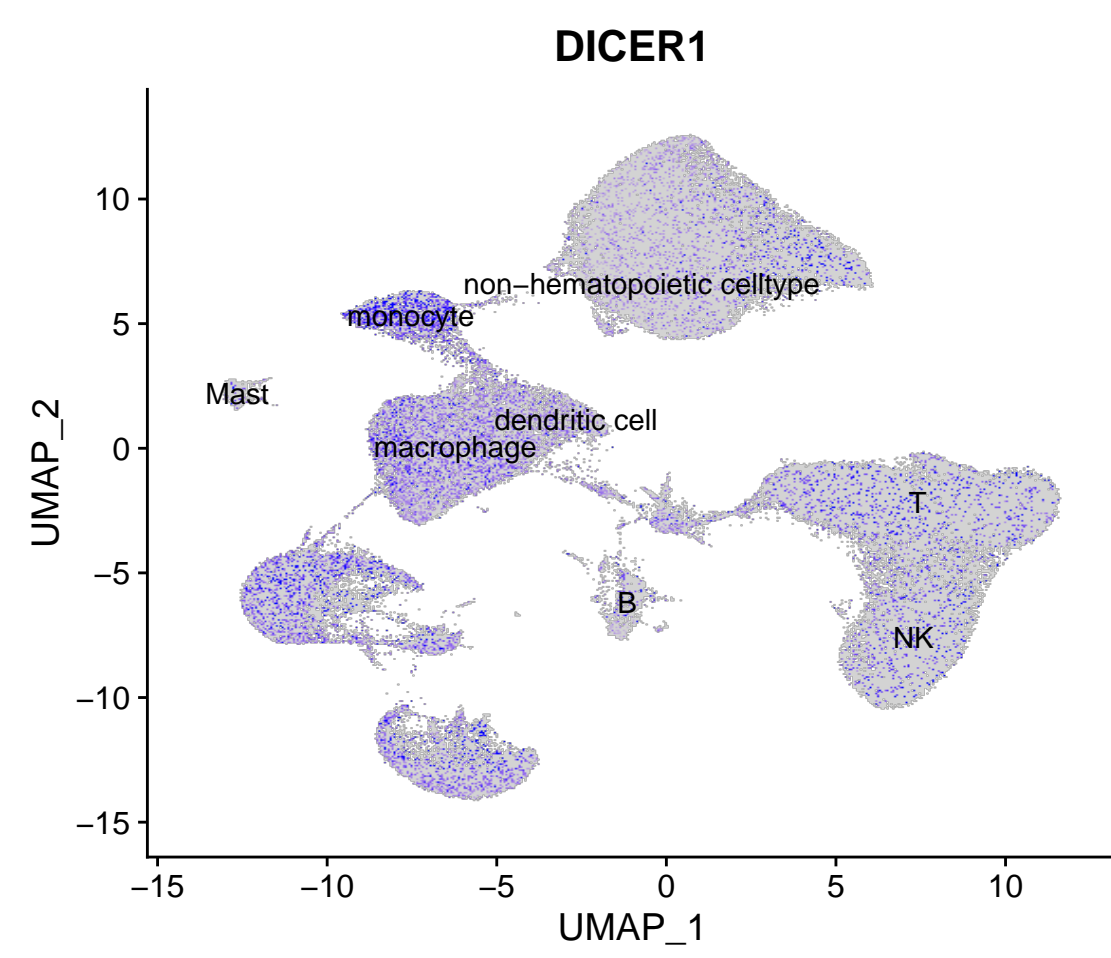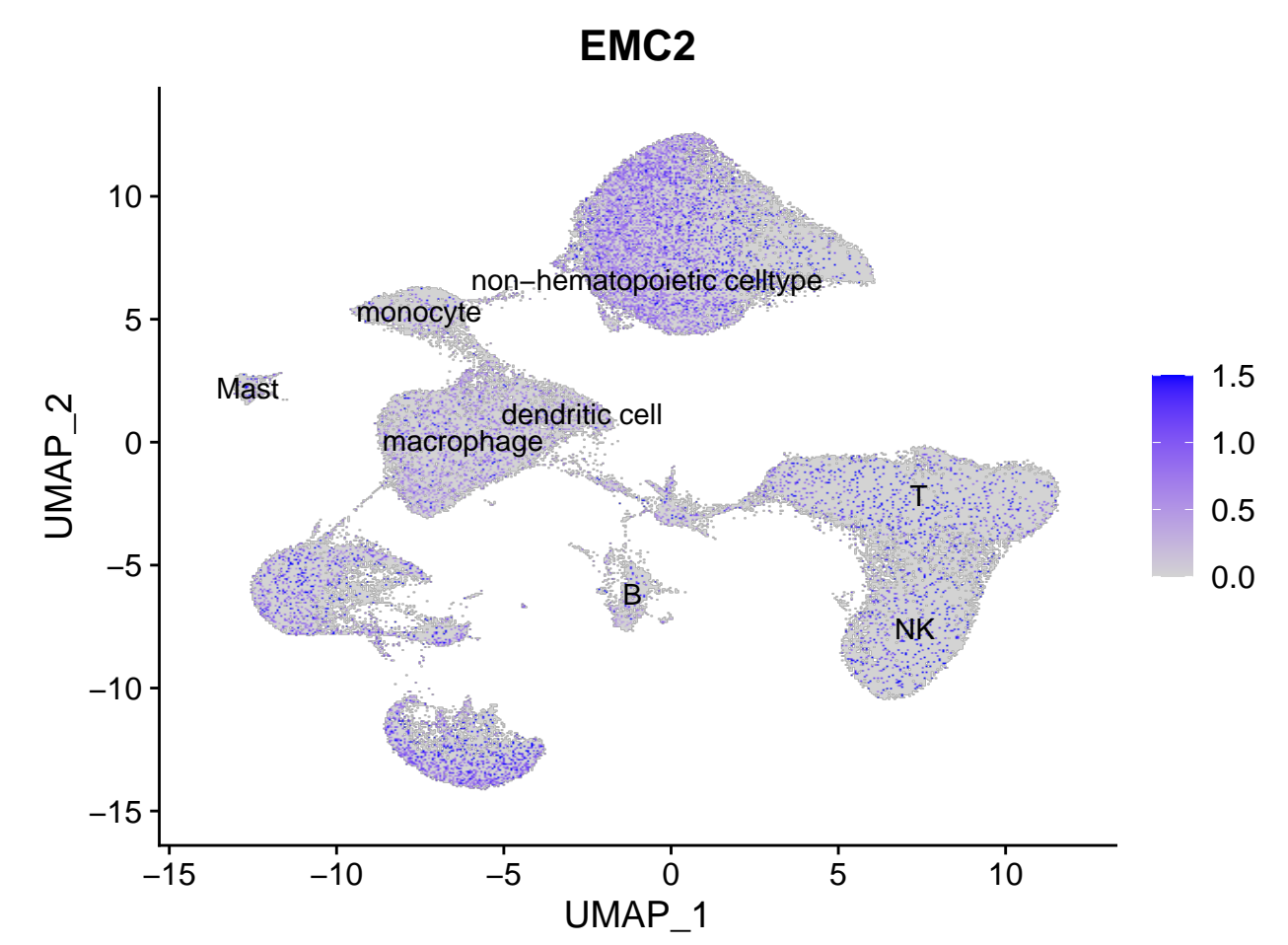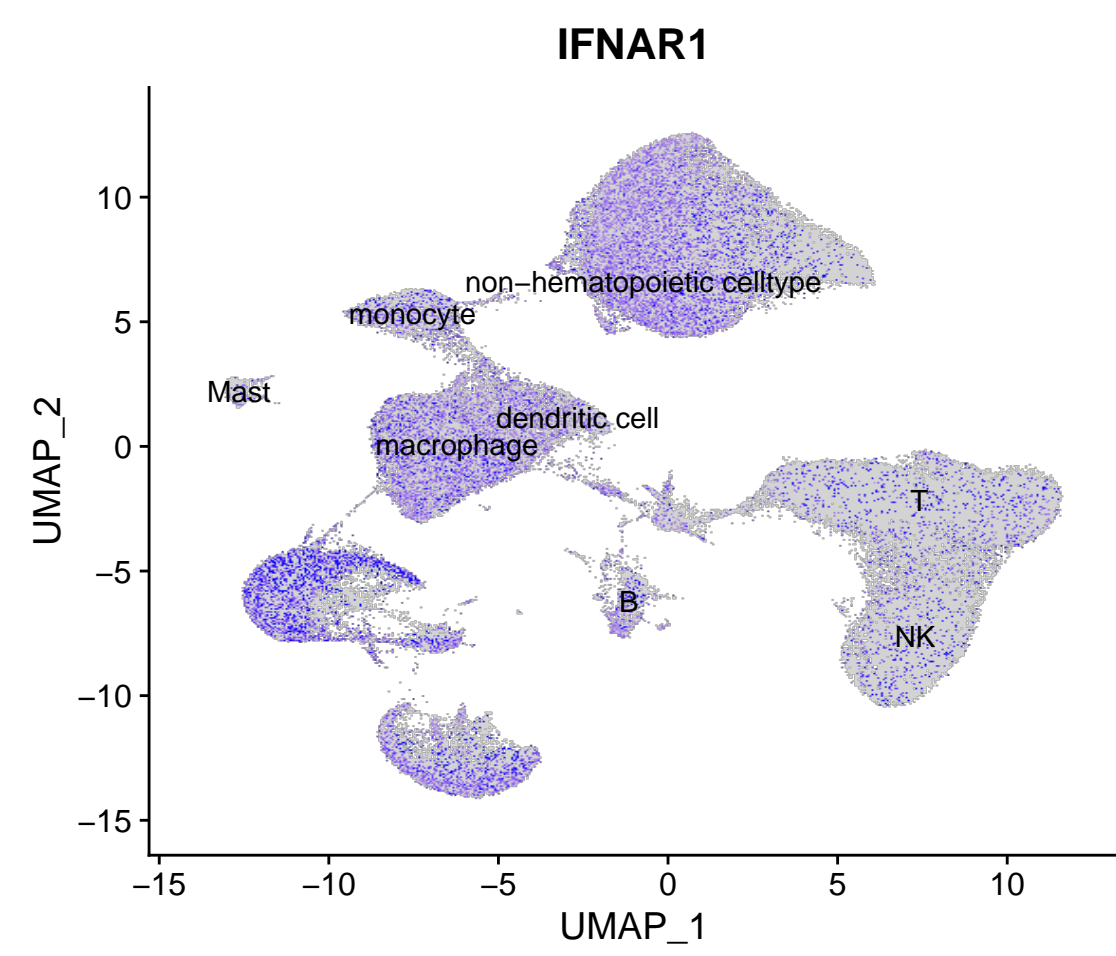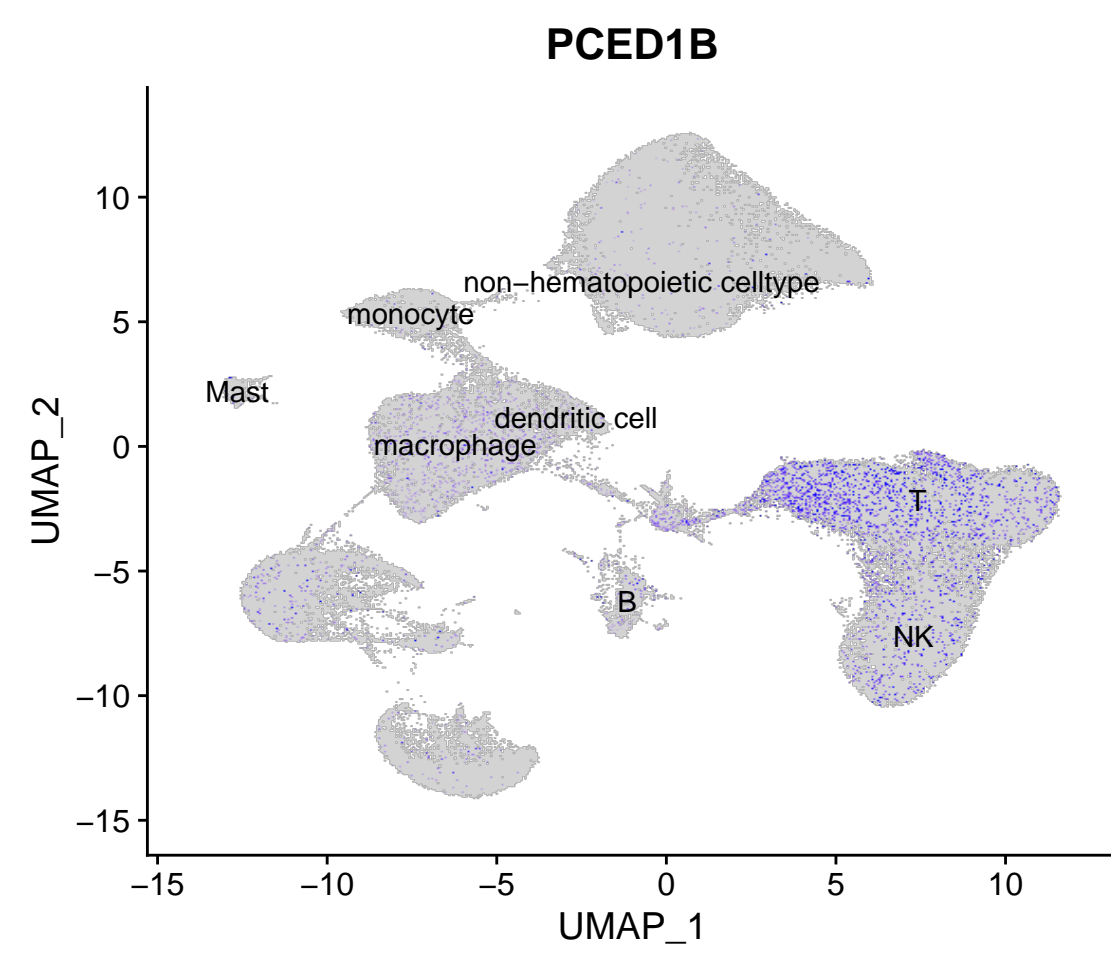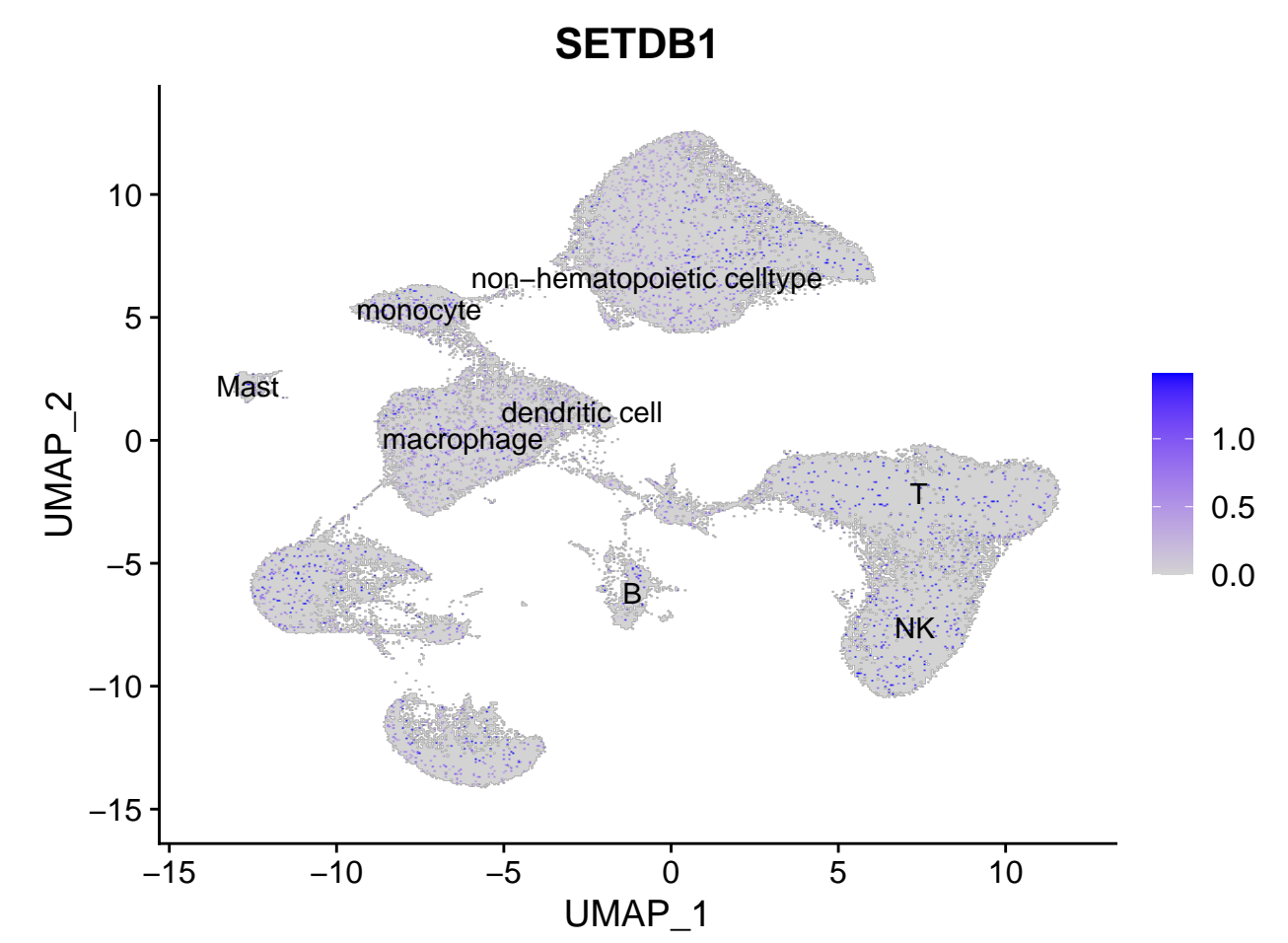

Supplement: SUPPLEMENTARY FIGURE 7 — Distribution and expression patterns of CTLEGs in single cells. [file Image_7.pdf]

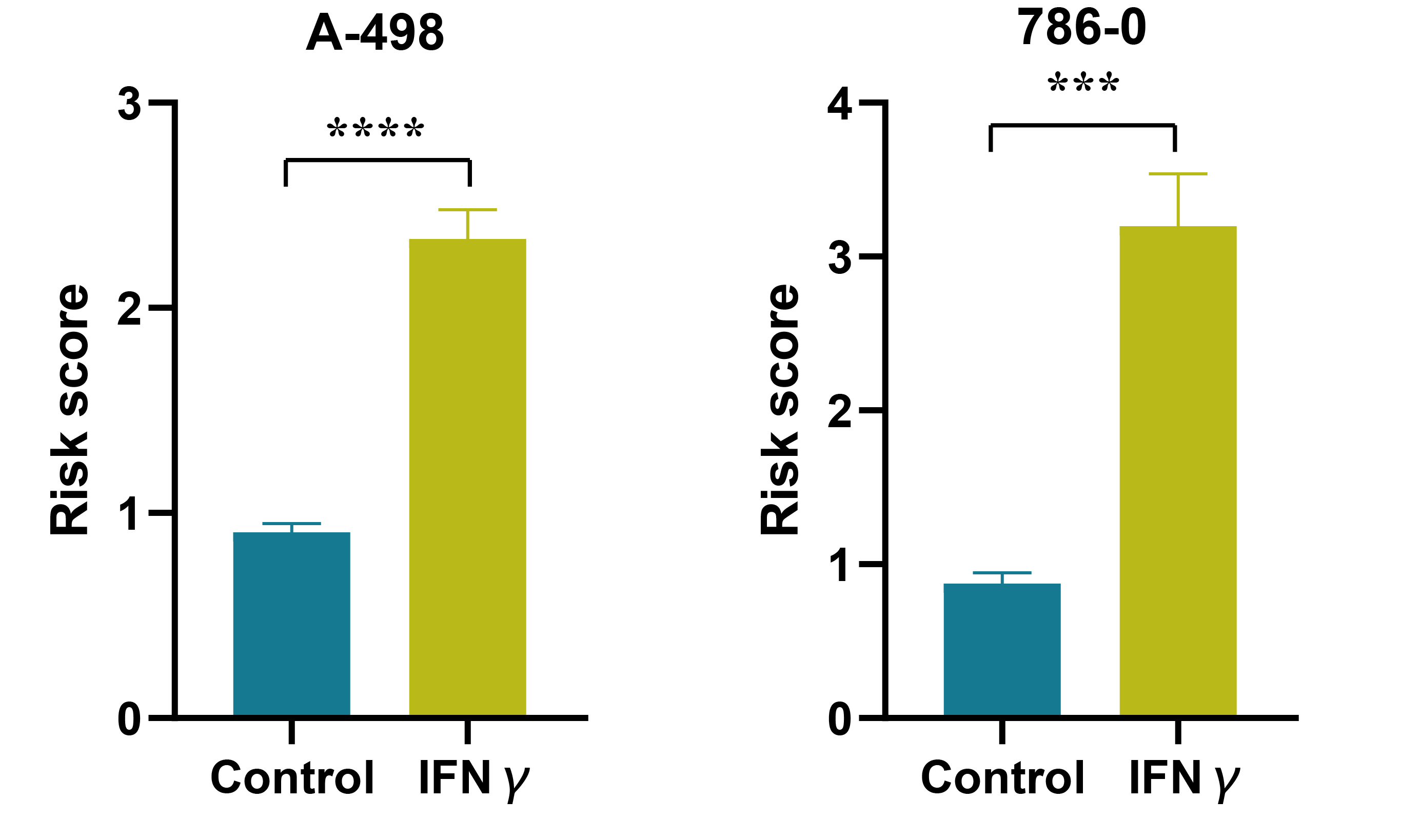

Supplement: SUPPLEMENTARY FIGURE 8 — Changes in risk score after interferon treatment. [file Image_8.jpeg]

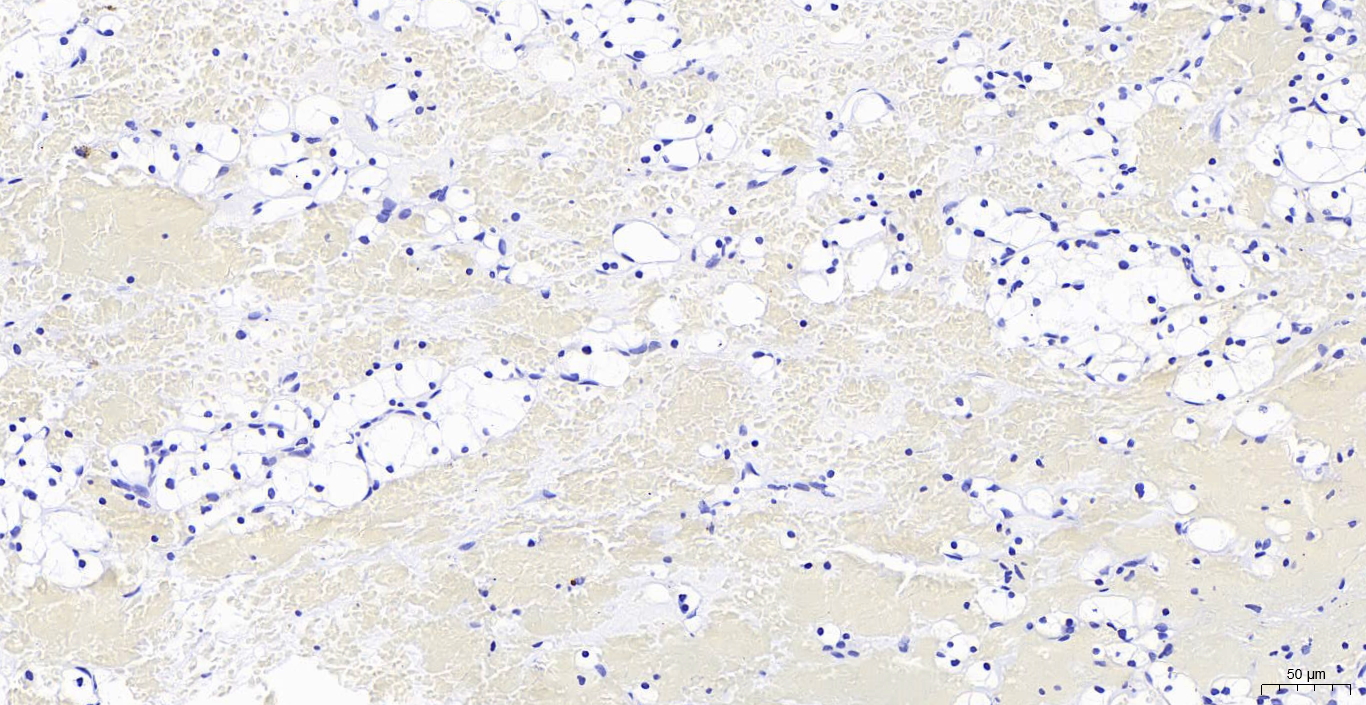

Supplement: Supplementary file 18 [file DataSheet_1.zip › Source data/IHC/High risk/D10-2.jpg]

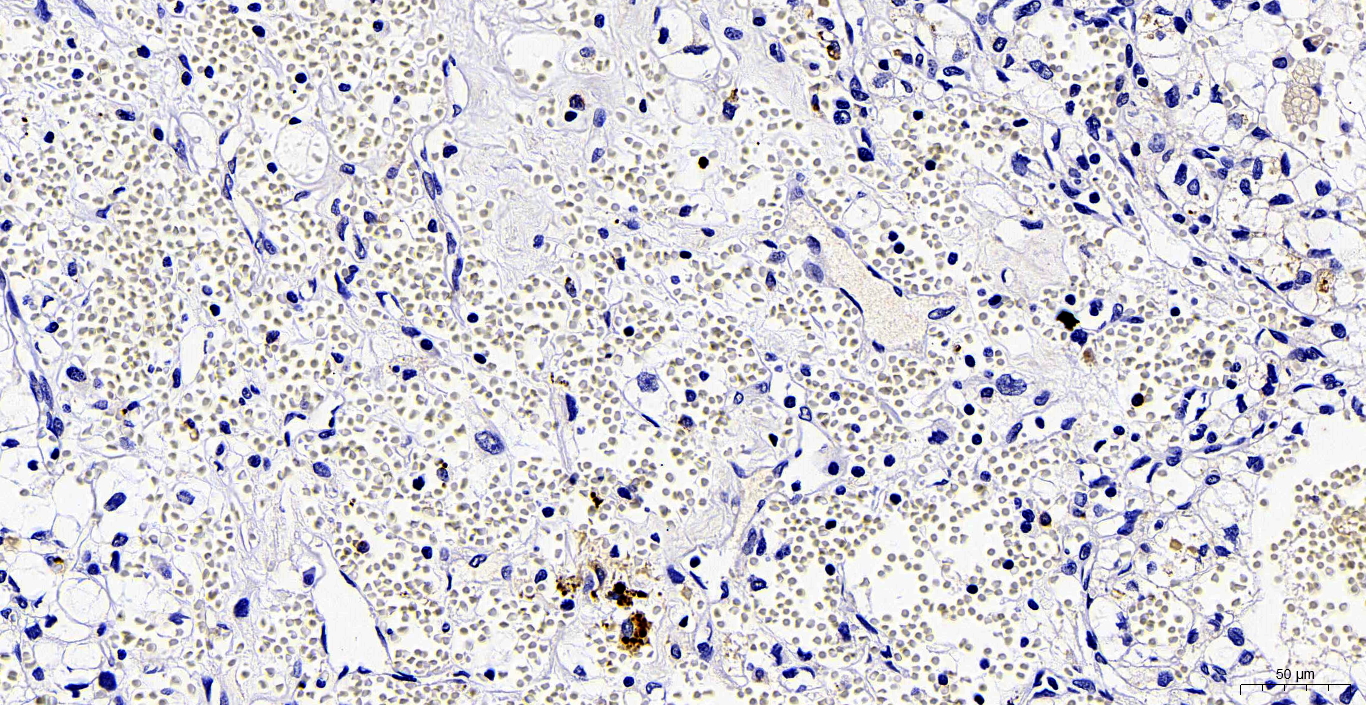

Supplement: Supplementary file 18 [file DataSheet_1.zip › Source data/IHC/High risk/E13.jpg]

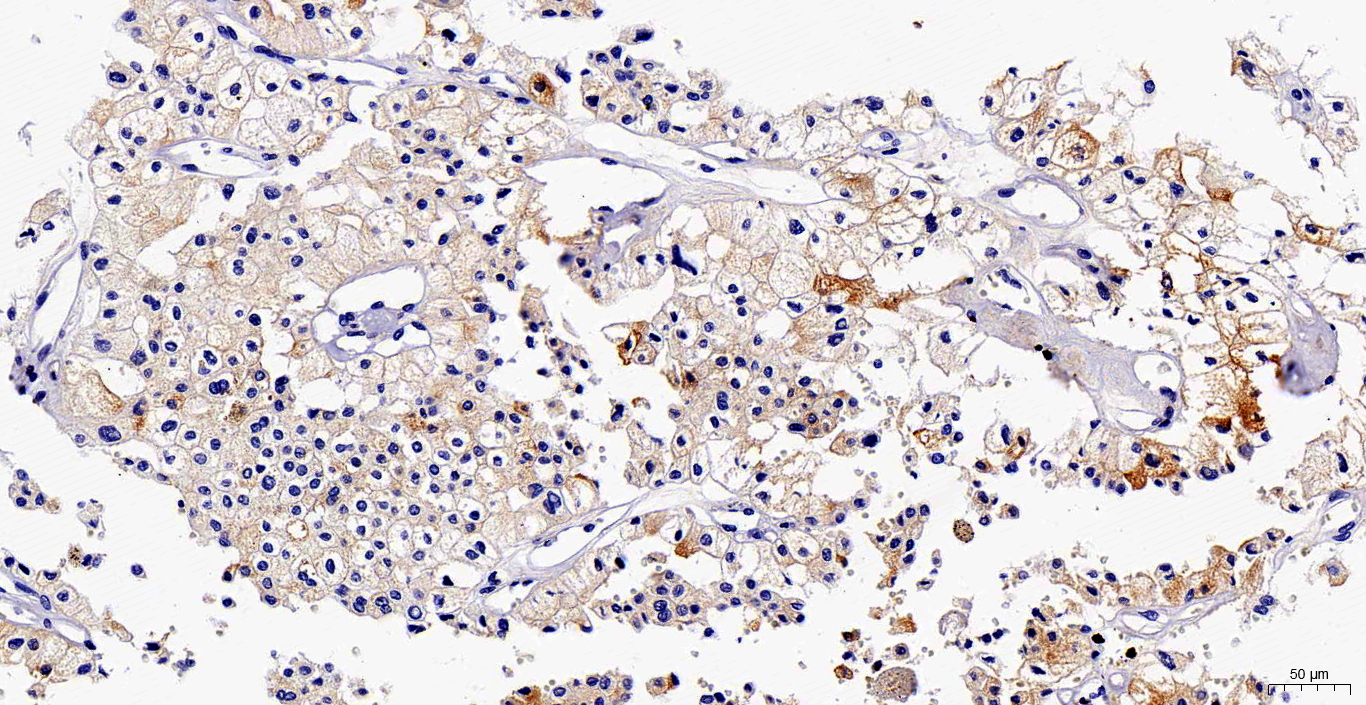

Supplement: Supplementary file 18 [file DataSheet_1.zip › Source data/IHC/High risk/F6.jpg]

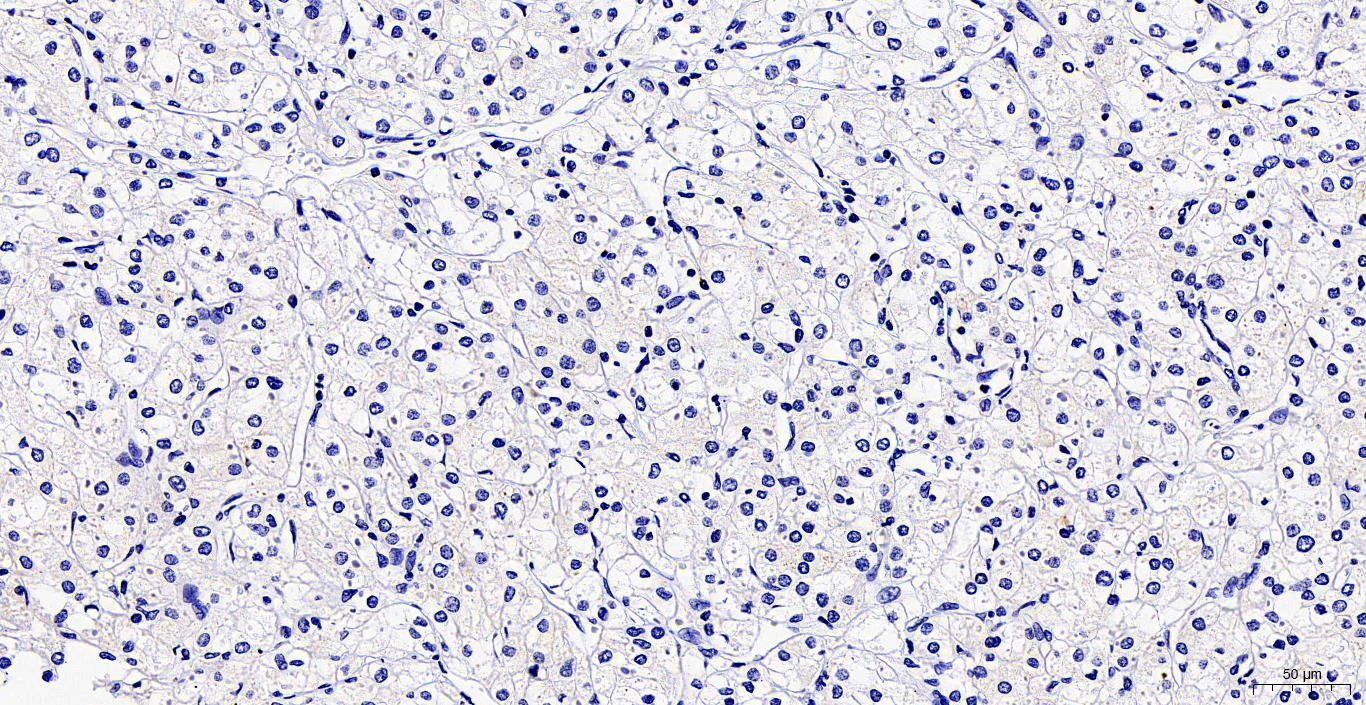

Supplement: Supplementary file 18 [file DataSheet_1.zip › Source data/IHC/low risk/B13.jpg]

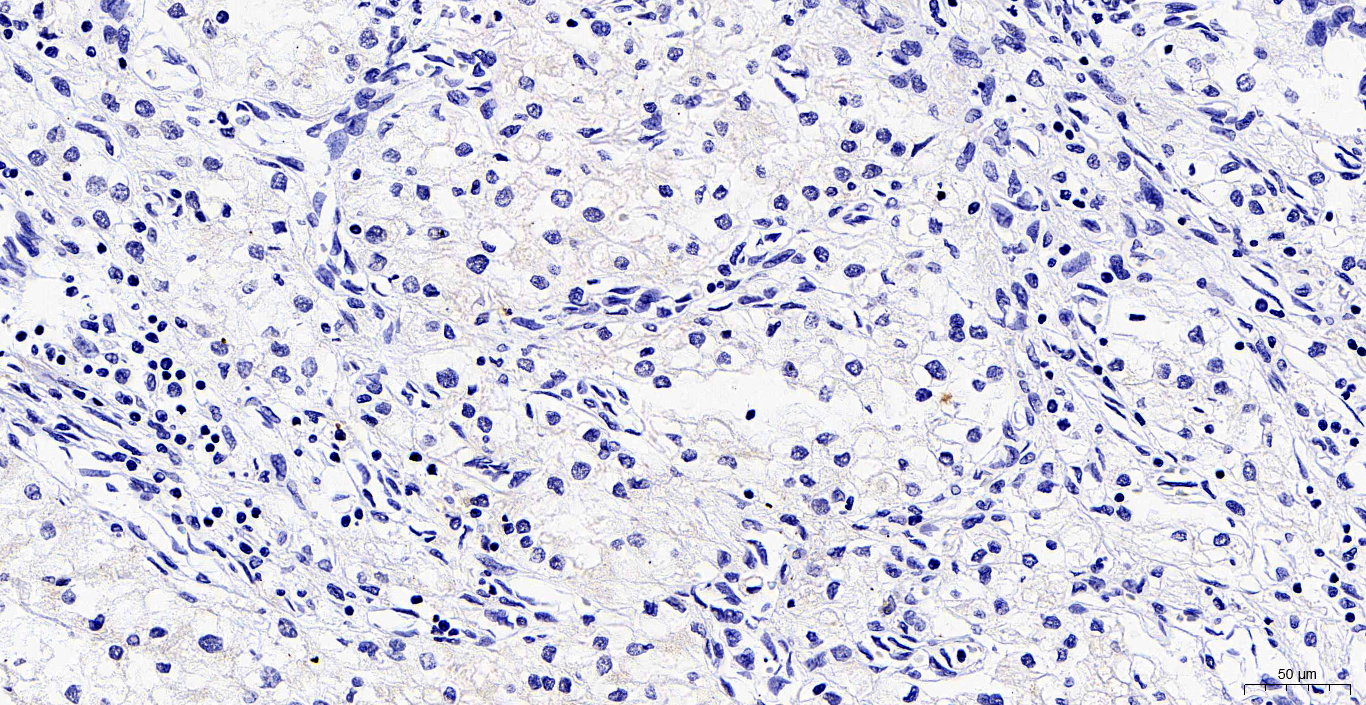

Supplement: Supplementary file 18 [file DataSheet_1.zip › Source data/IHC/low risk/B9.2x.jpg]

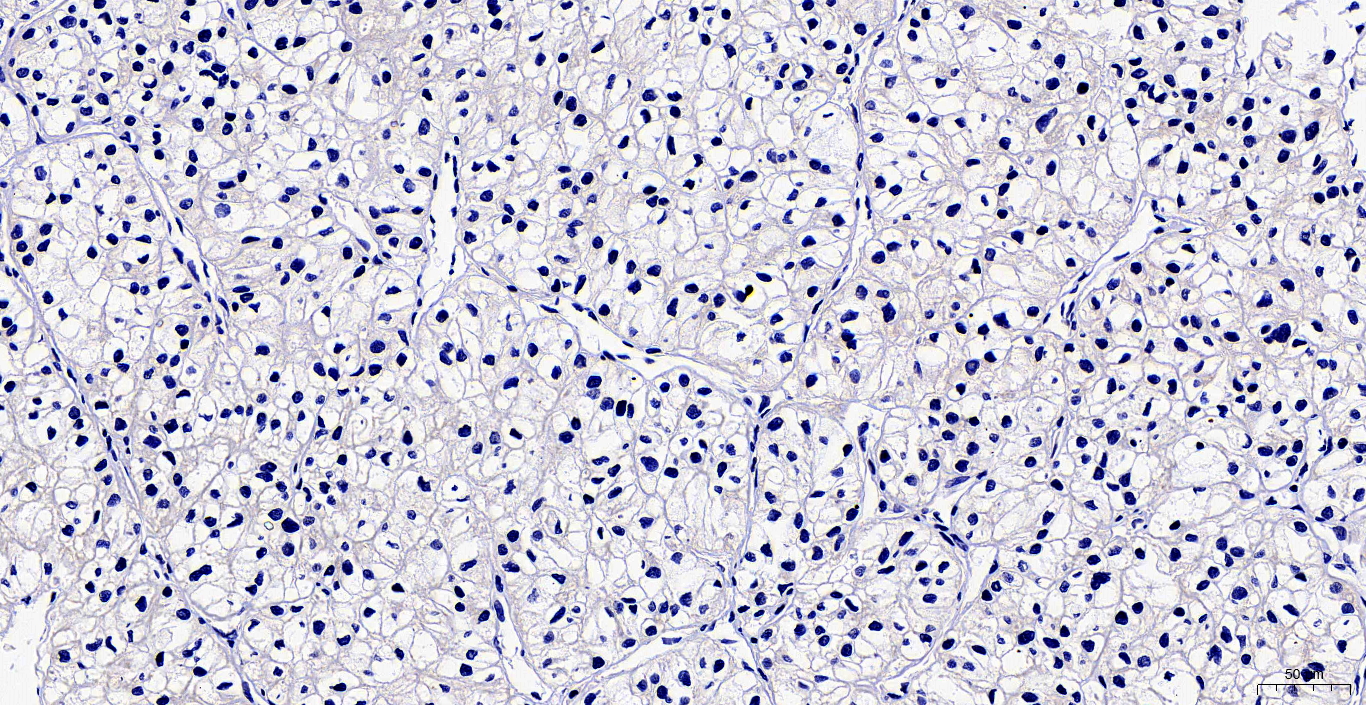

Supplement: Supplementary file 18 [file DataSheet_1.zip › Source data/IHC/low risk/E9.jpg]

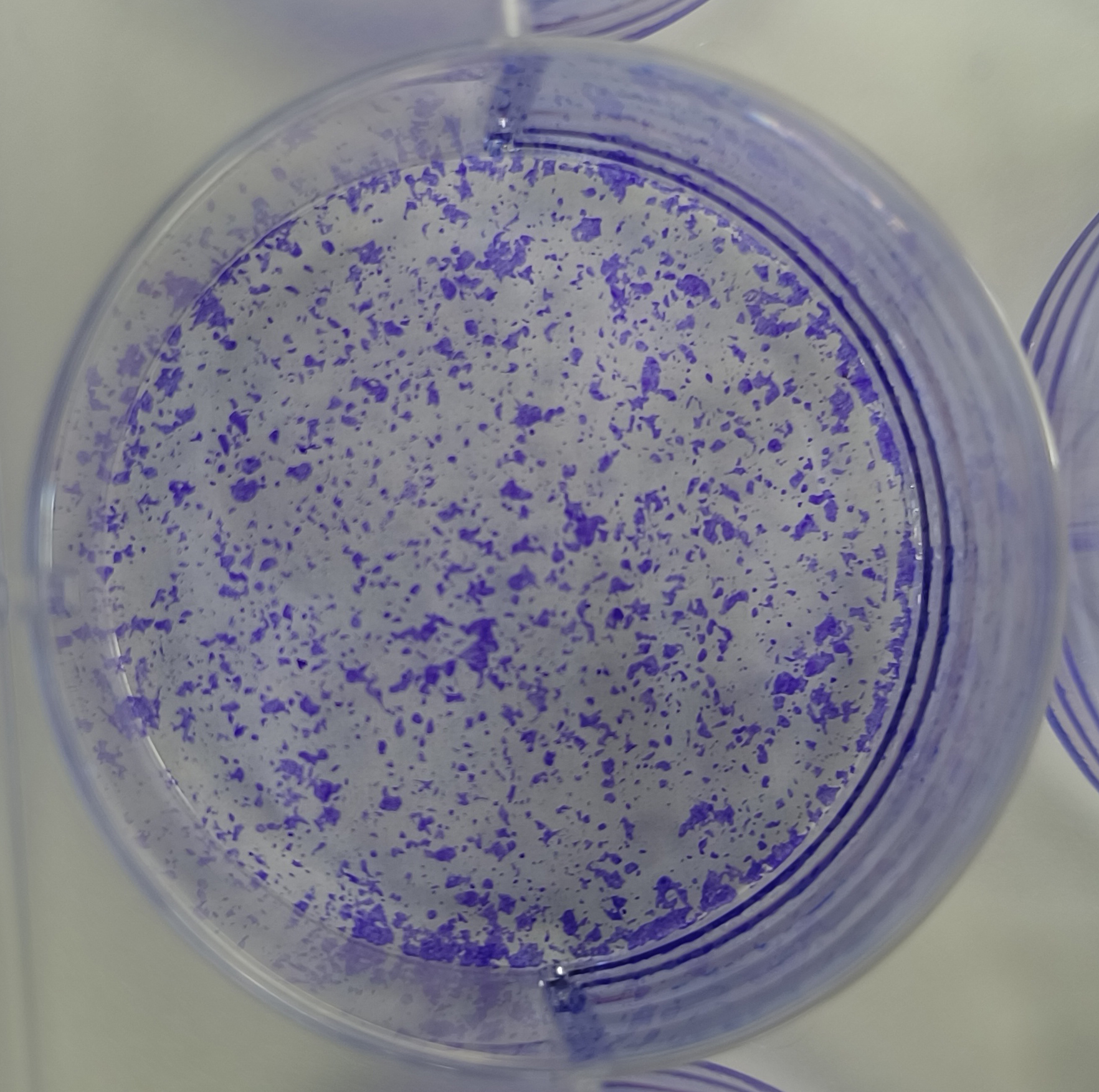

Supplement: Supplementary file 18 [file DataSheet_1.zip › Source data/colony/769-P/NC/1.jpg]

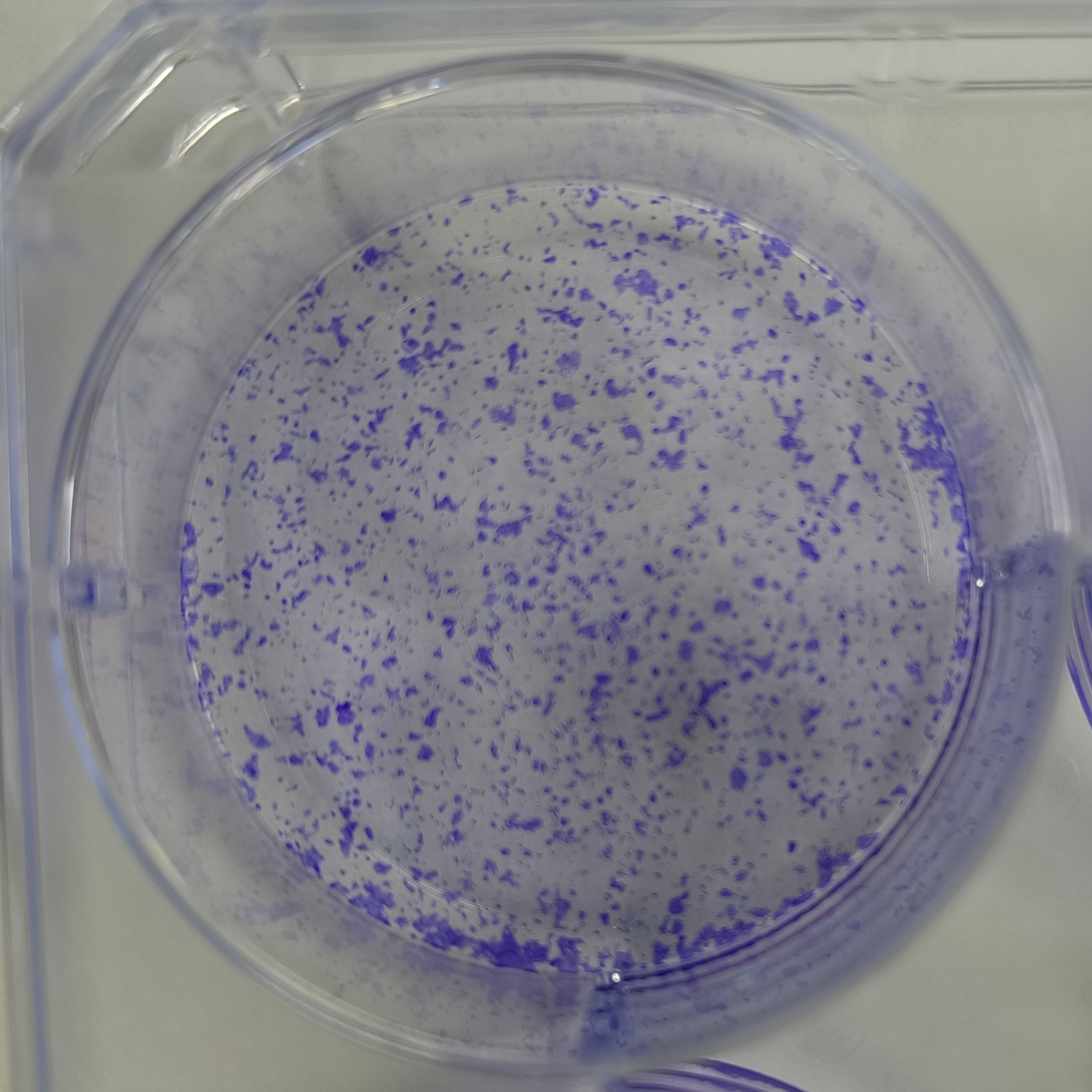

Supplement: Supplementary file 18 [file DataSheet_1.zip › Source data/colony/769-P/NC/2.jpg]

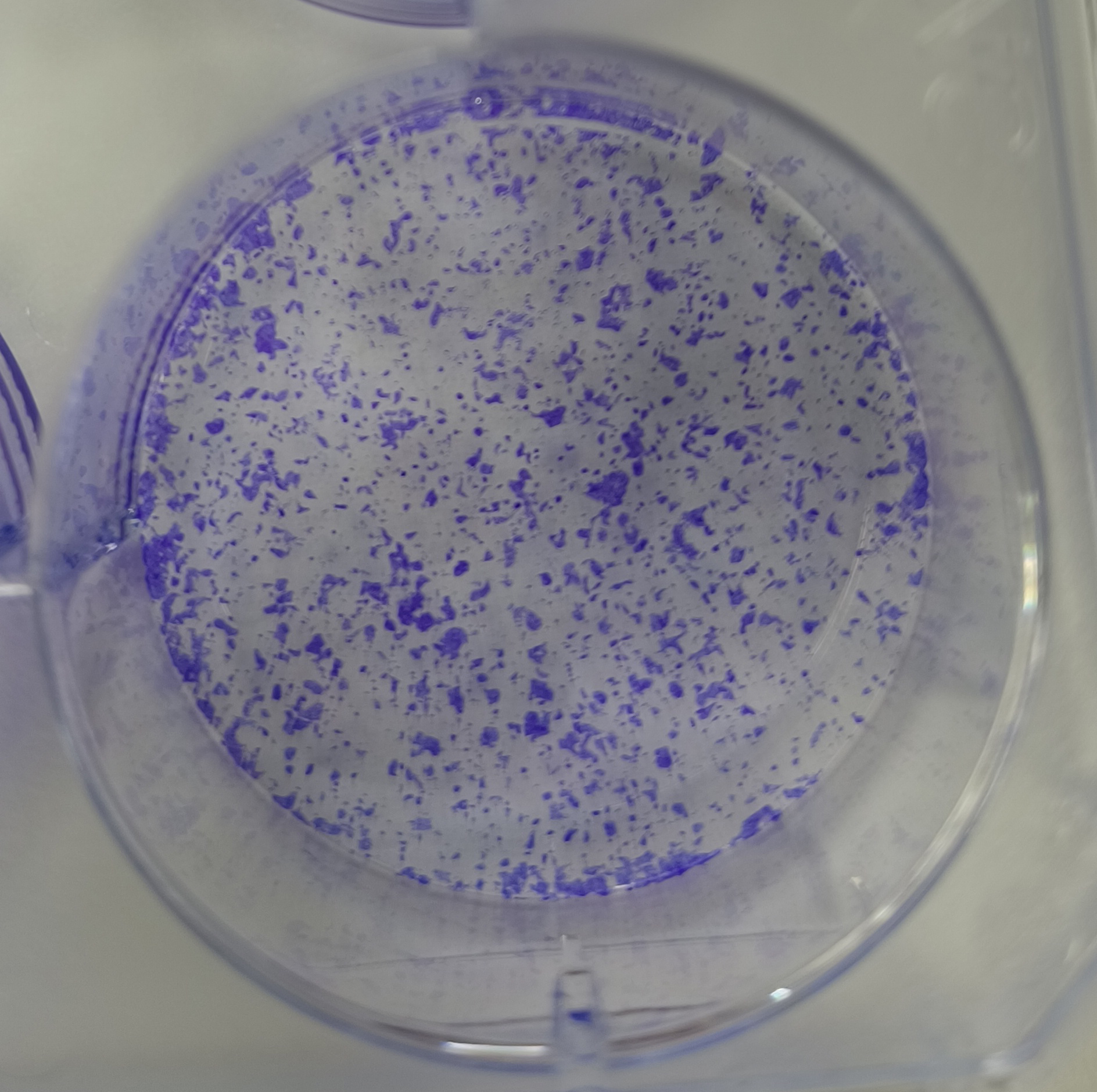

Supplement: Supplementary file 18 [file DataSheet_1.zip › Source data/colony/769-P/NC/3.jpg]

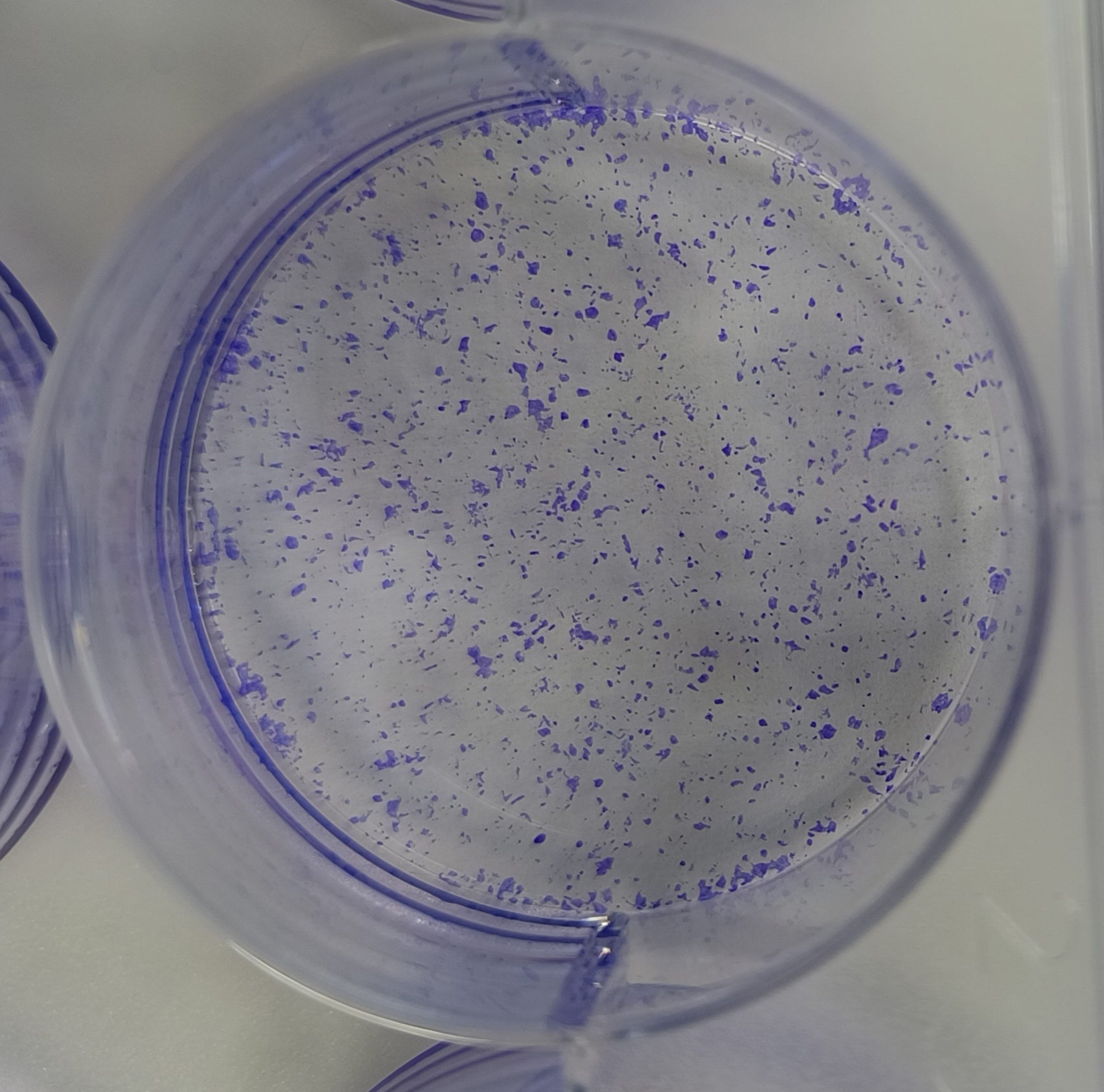

Supplement: Supplementary file 18 [file DataSheet_1.zip › Source data/colony/769-P/siSTAT2-1/1.jpg]

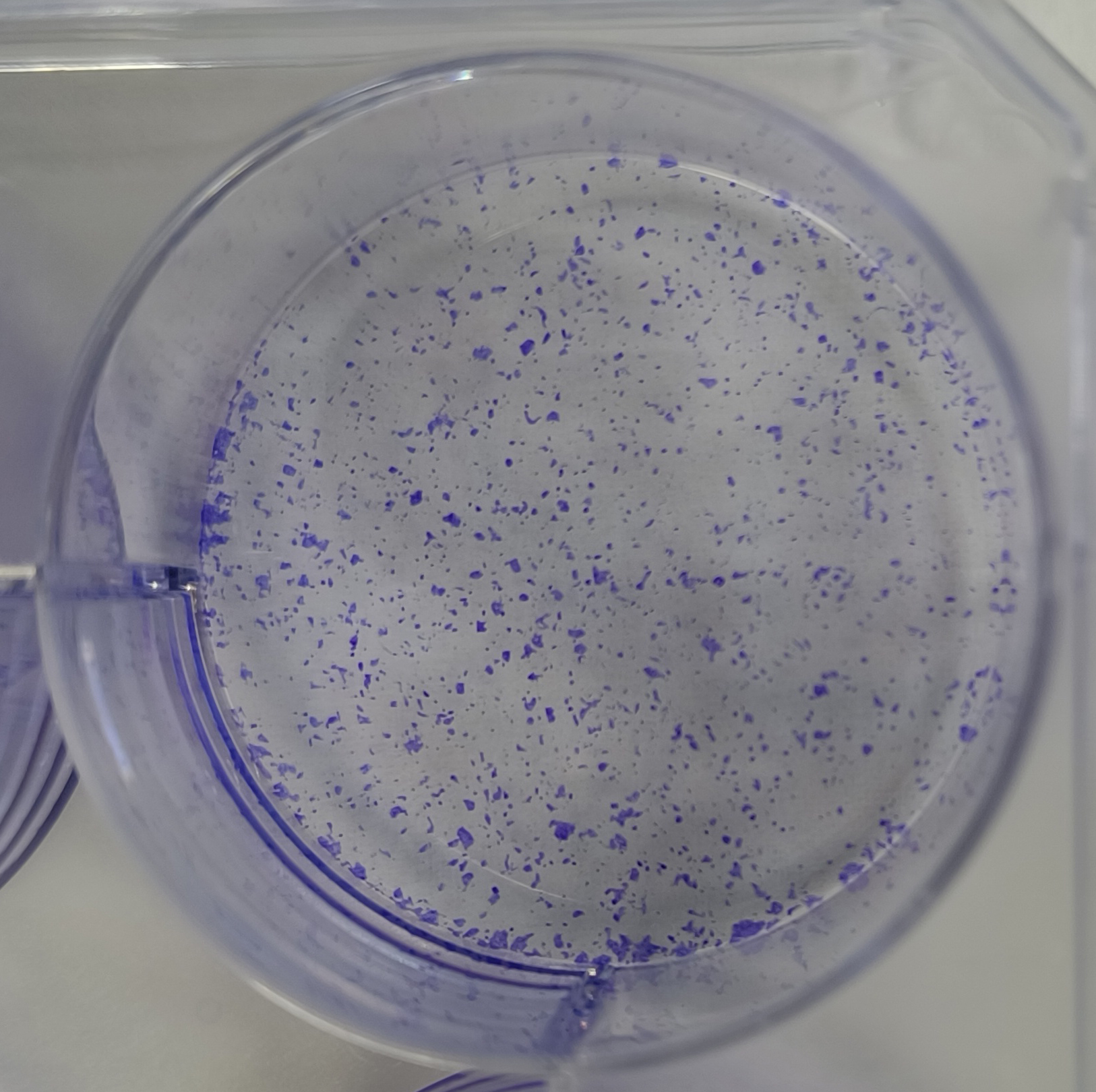

Supplement: Supplementary file 18 [file DataSheet_1.zip › Source data/colony/769-P/siSTAT2-1/2.jpg]

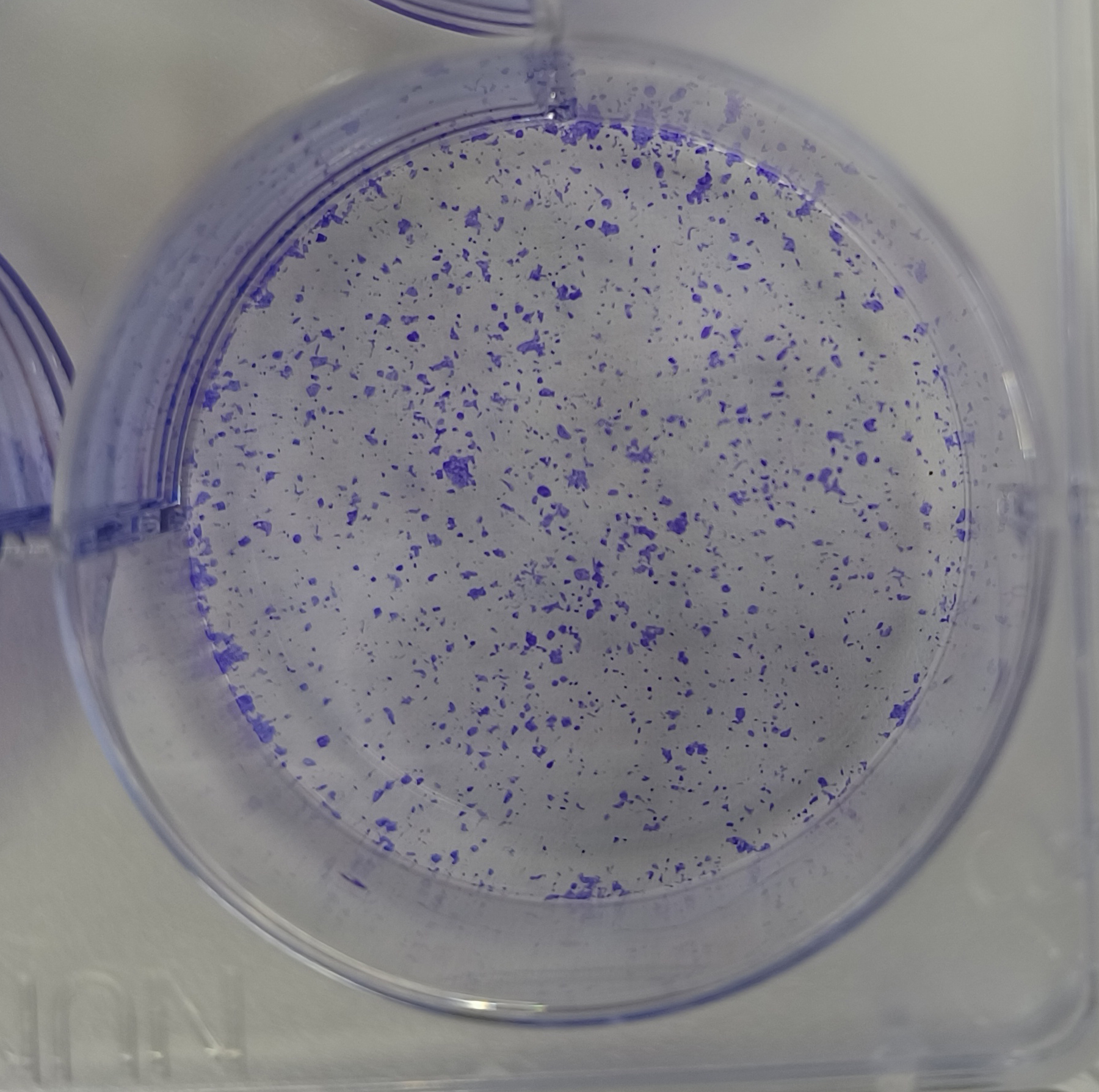

Supplement: Supplementary file 18 [file DataSheet_1.zip › Source data/colony/769-P/siSTAT2-1/3.jpg]

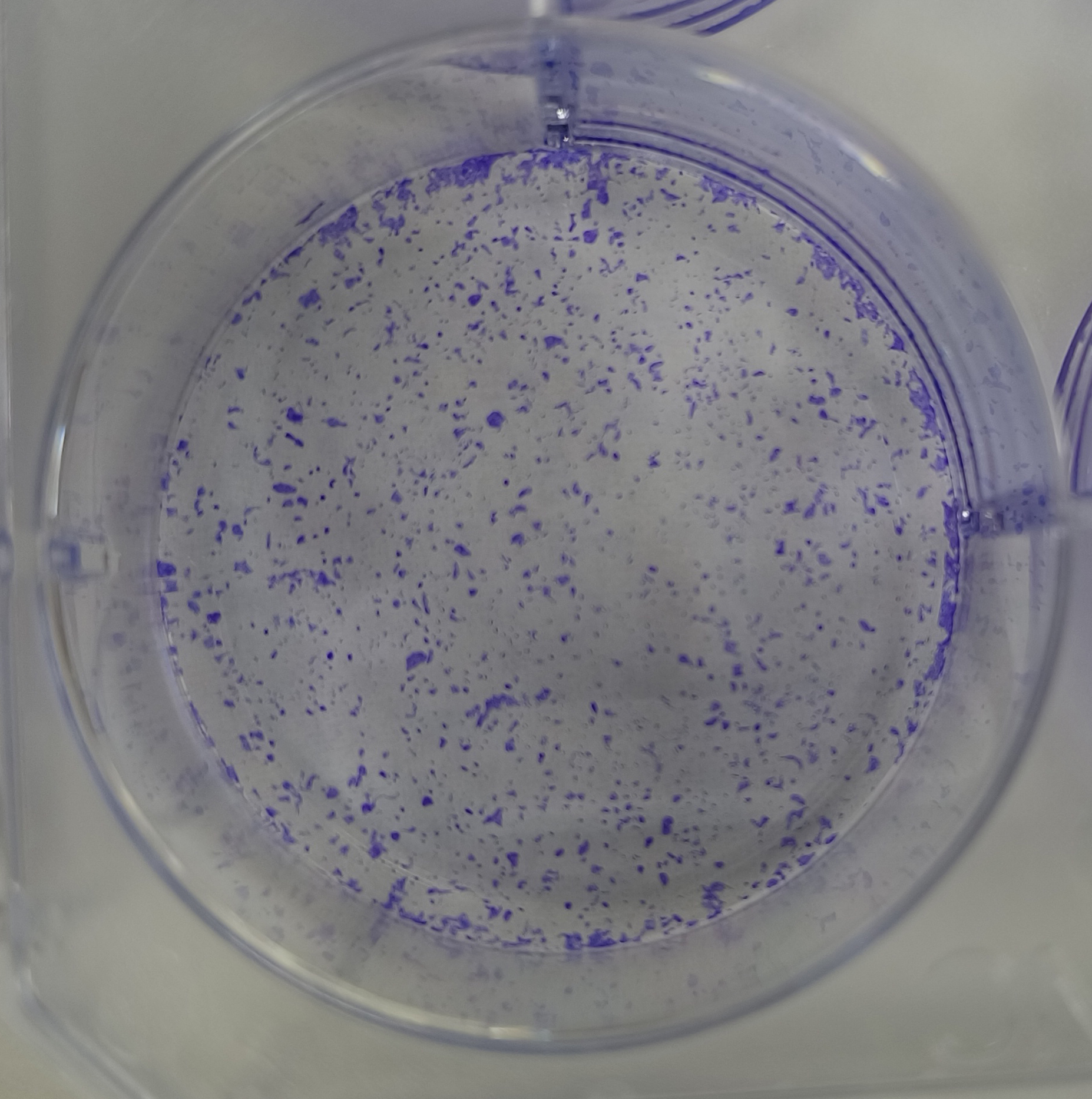

Supplement: Supplementary file 18 [file DataSheet_1.zip › Source data/colony/769-P/siSTAT2-2/1.jpg]

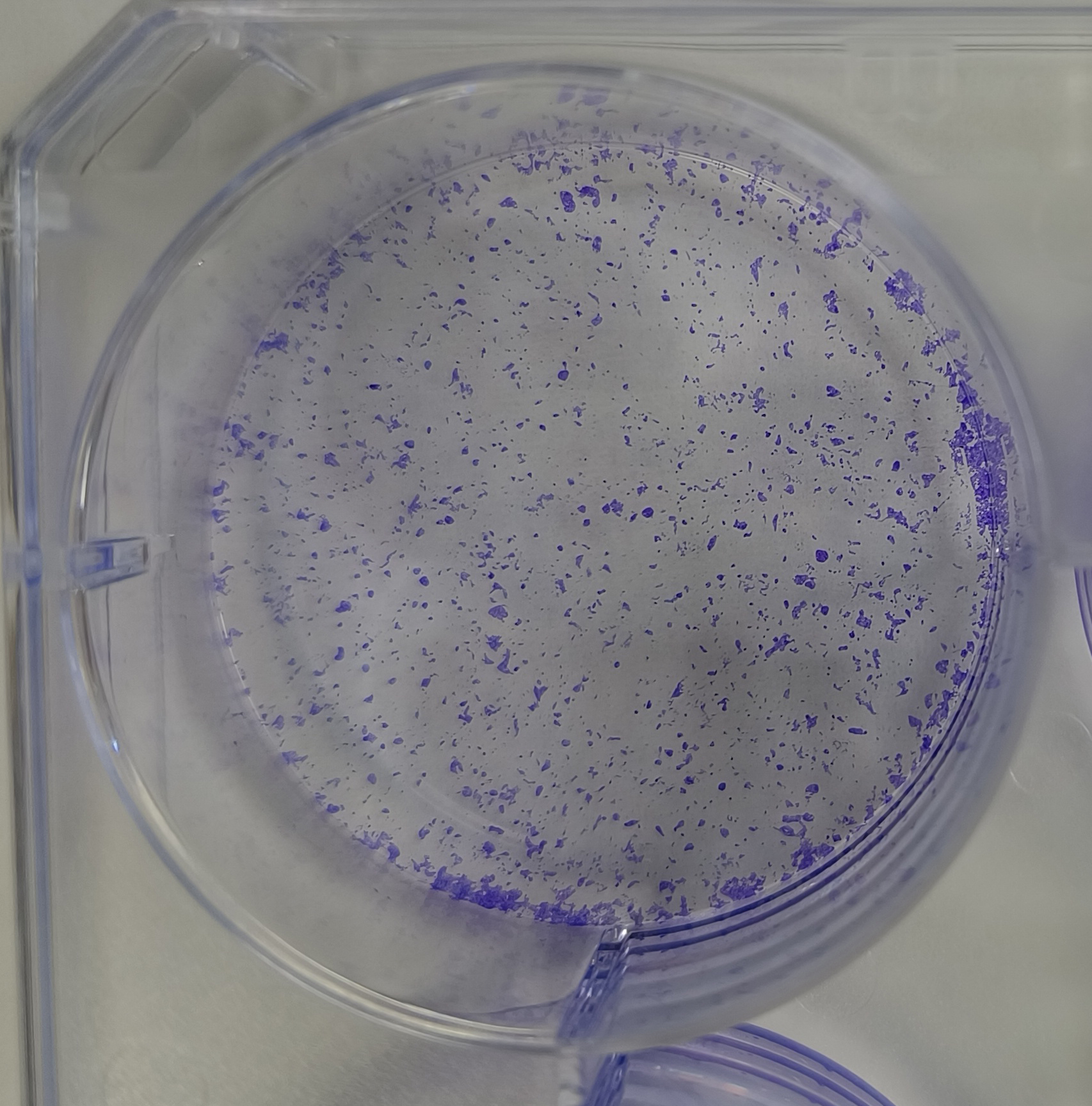

Supplement: Supplementary file 18 [file DataSheet_1.zip › Source data/colony/769-P/siSTAT2-2/2.jpg]

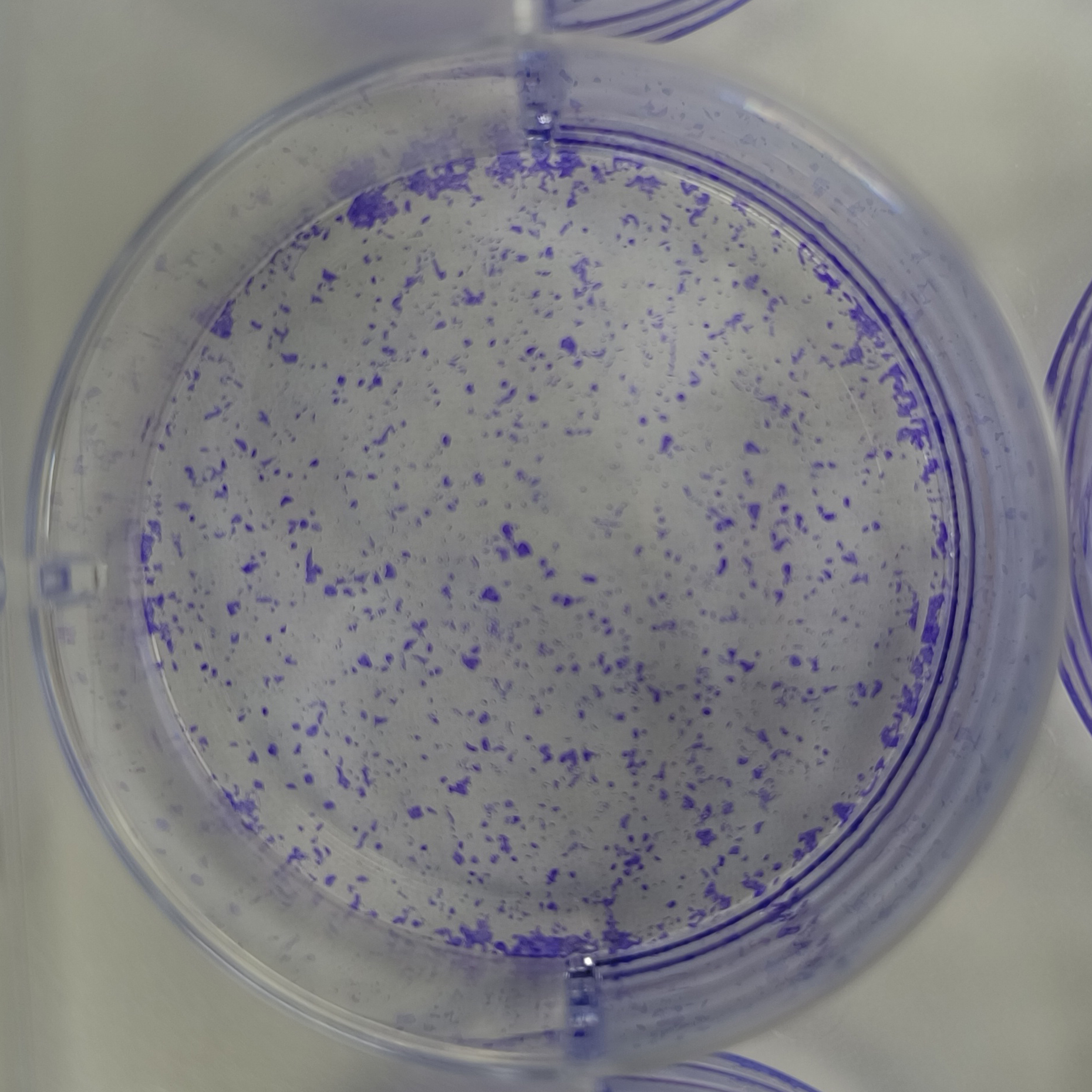

Supplement: Supplementary file 18 [file DataSheet_1.zip › Source data/colony/769-P/siSTAT2-2/3.jpg]

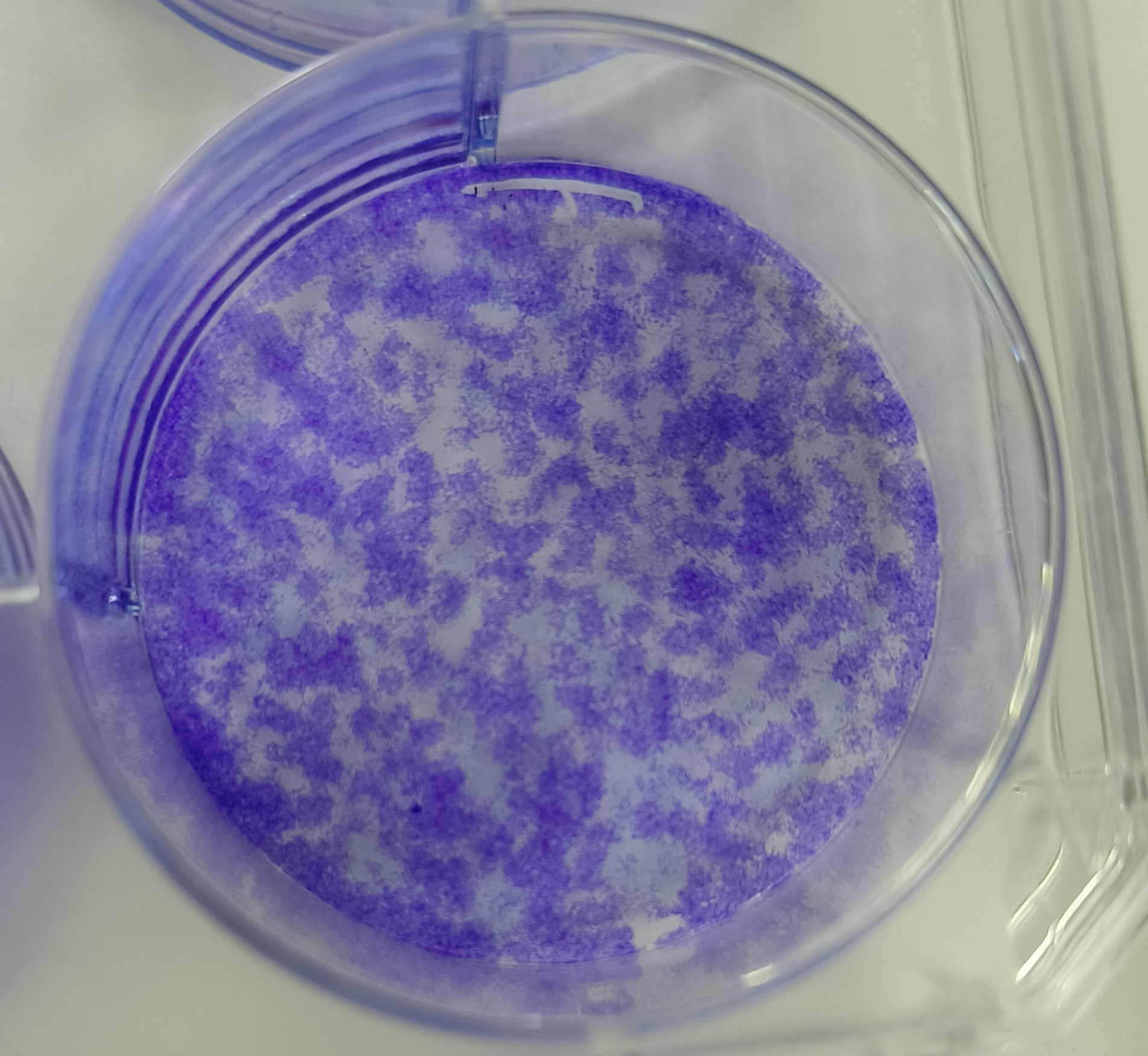

Supplement: Supplementary file 18 [file DataSheet_1.zip › Source data/colony/786-0/NC/1.jpg]

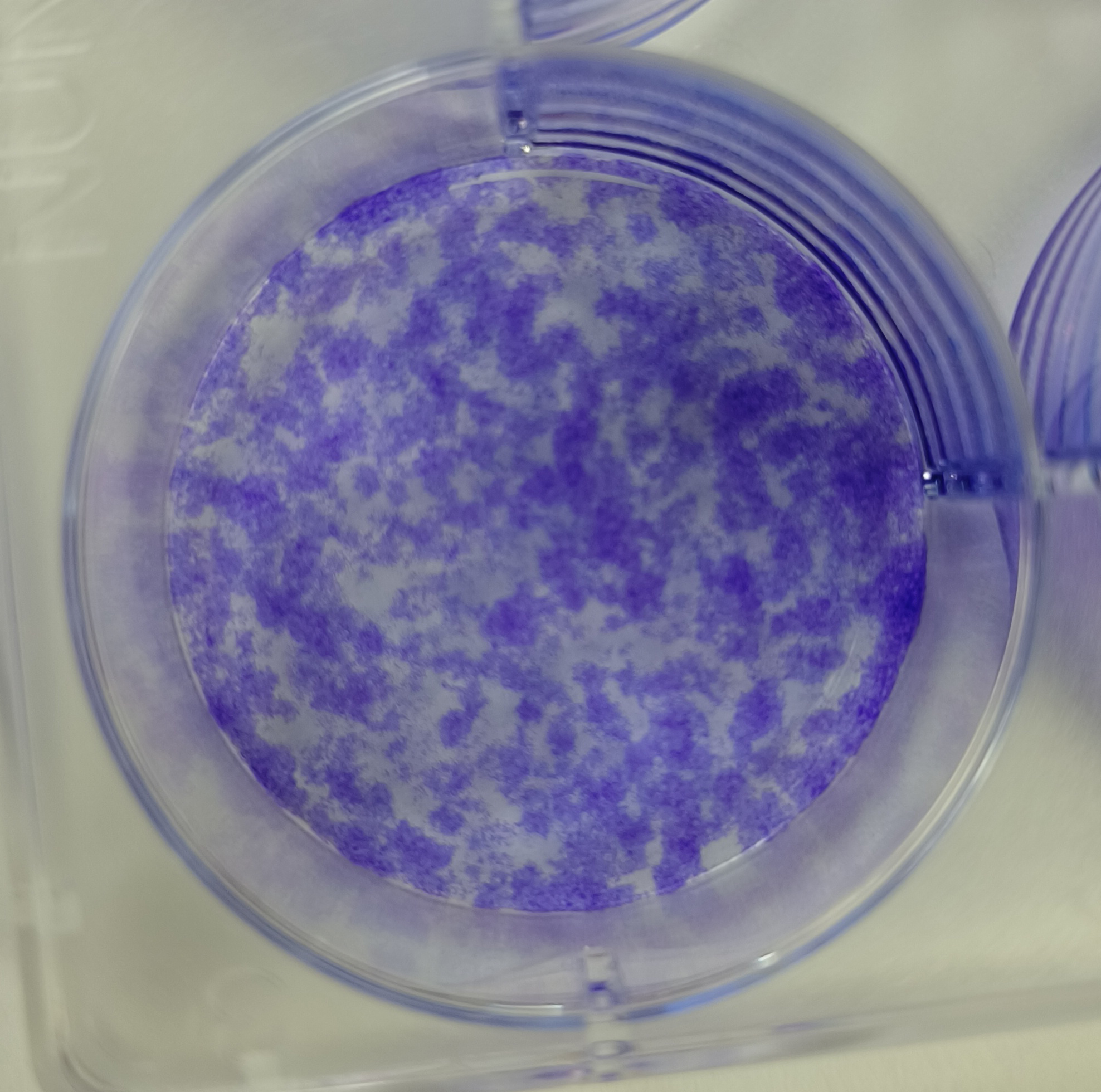

Supplement: Supplementary file 18 [file DataSheet_1.zip › Source data/colony/786-0/NC/2.jpg]

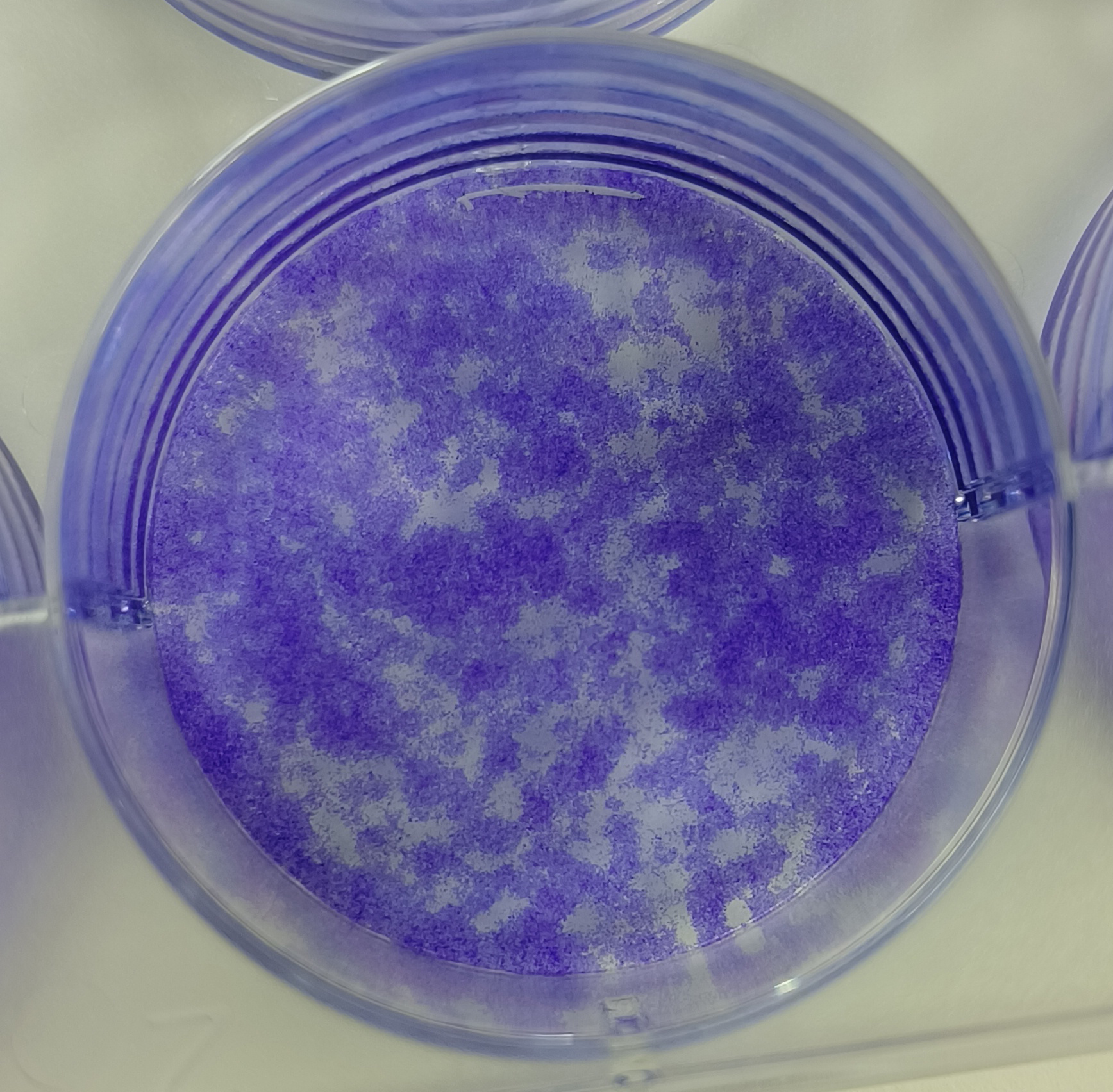

Supplement: Supplementary file 18 [file DataSheet_1.zip › Source data/colony/786-0/NC/3.jpg]

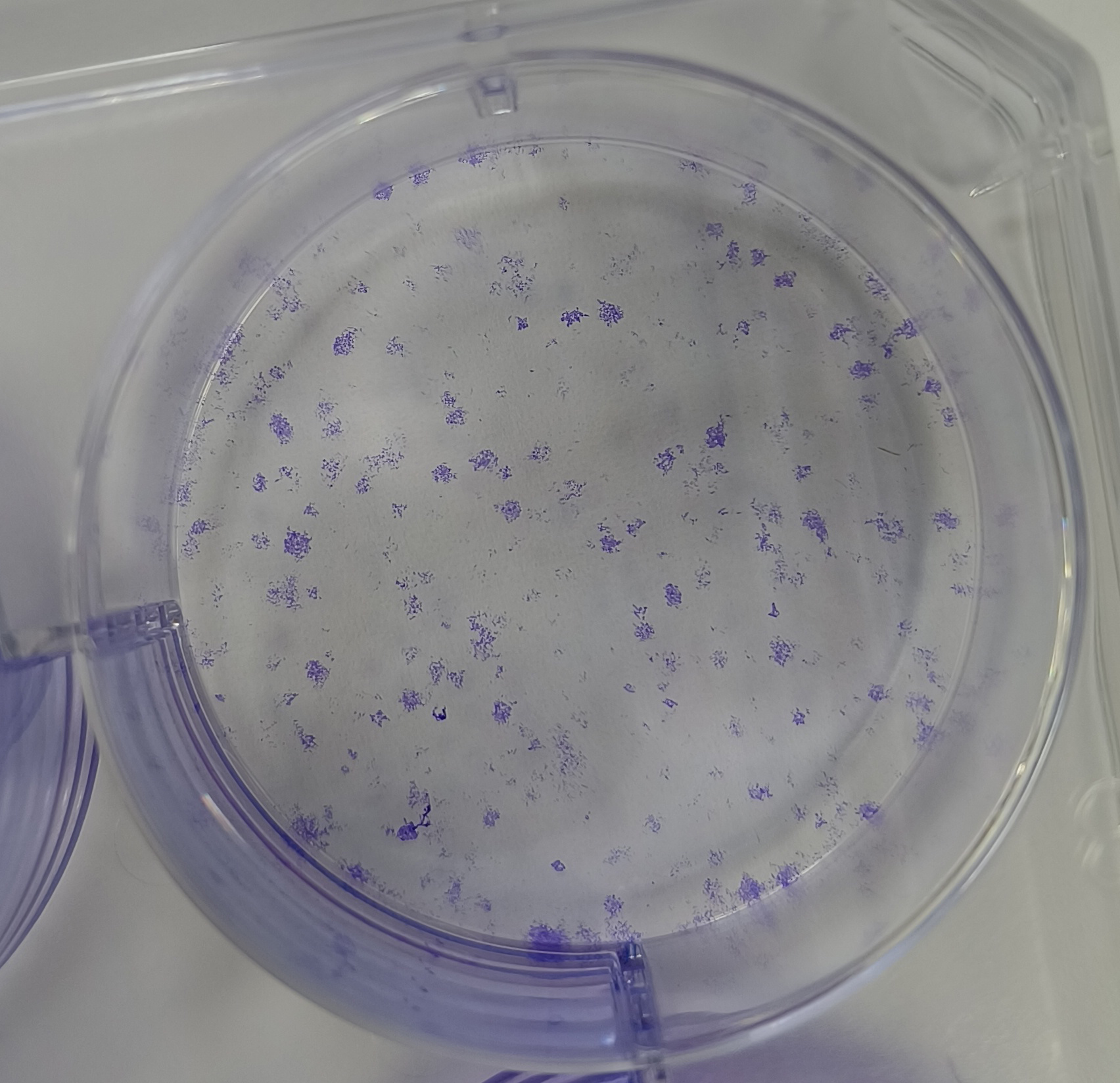

Supplement: Supplementary file 18 [file DataSheet_1.zip › Source data/colony/786-0/siSTAT2-1/1.jpg]

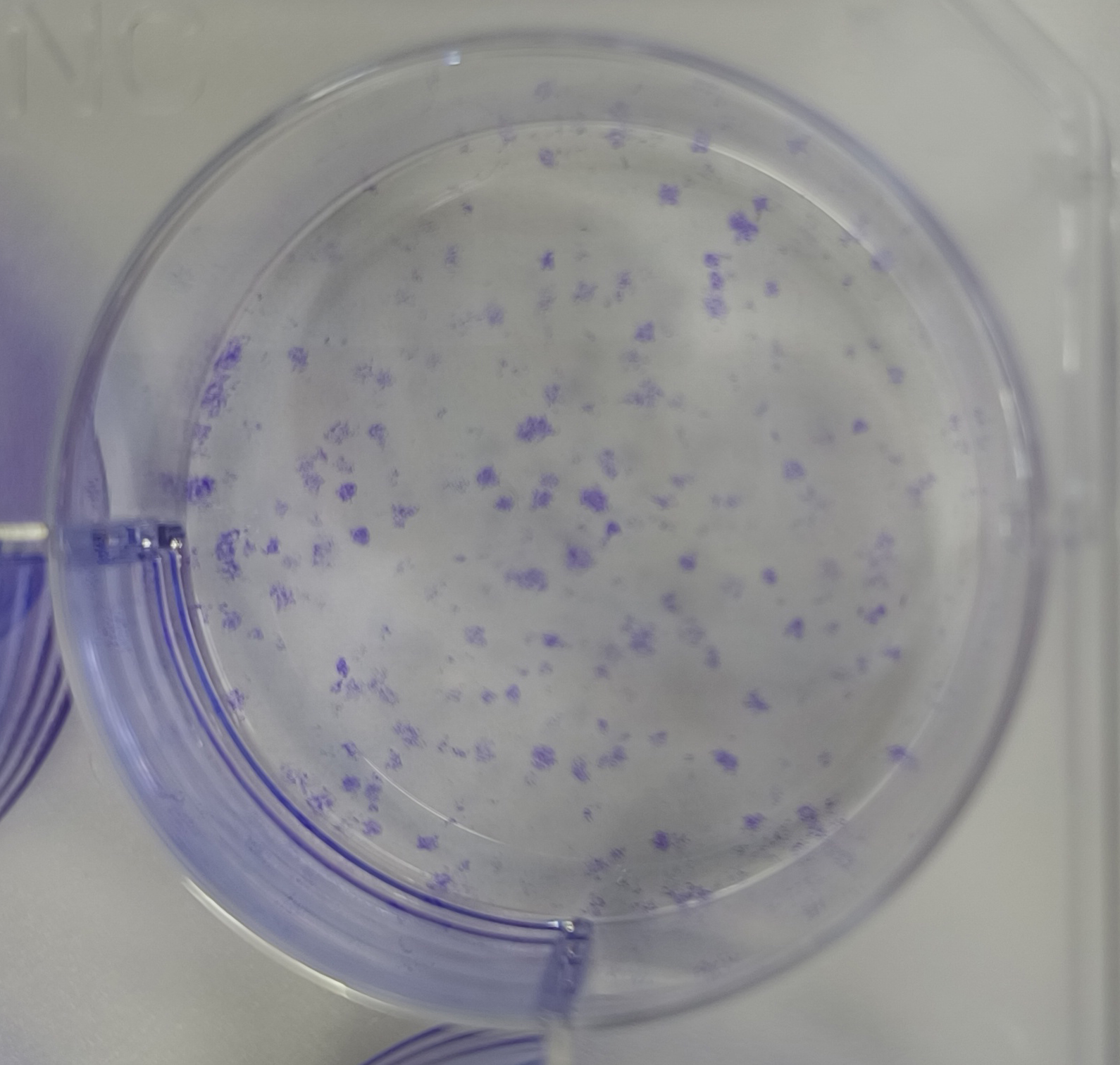

Supplement: Supplementary file 18 [file DataSheet_1.zip › Source data/colony/786-0/siSTAT2-1/2.jpg]

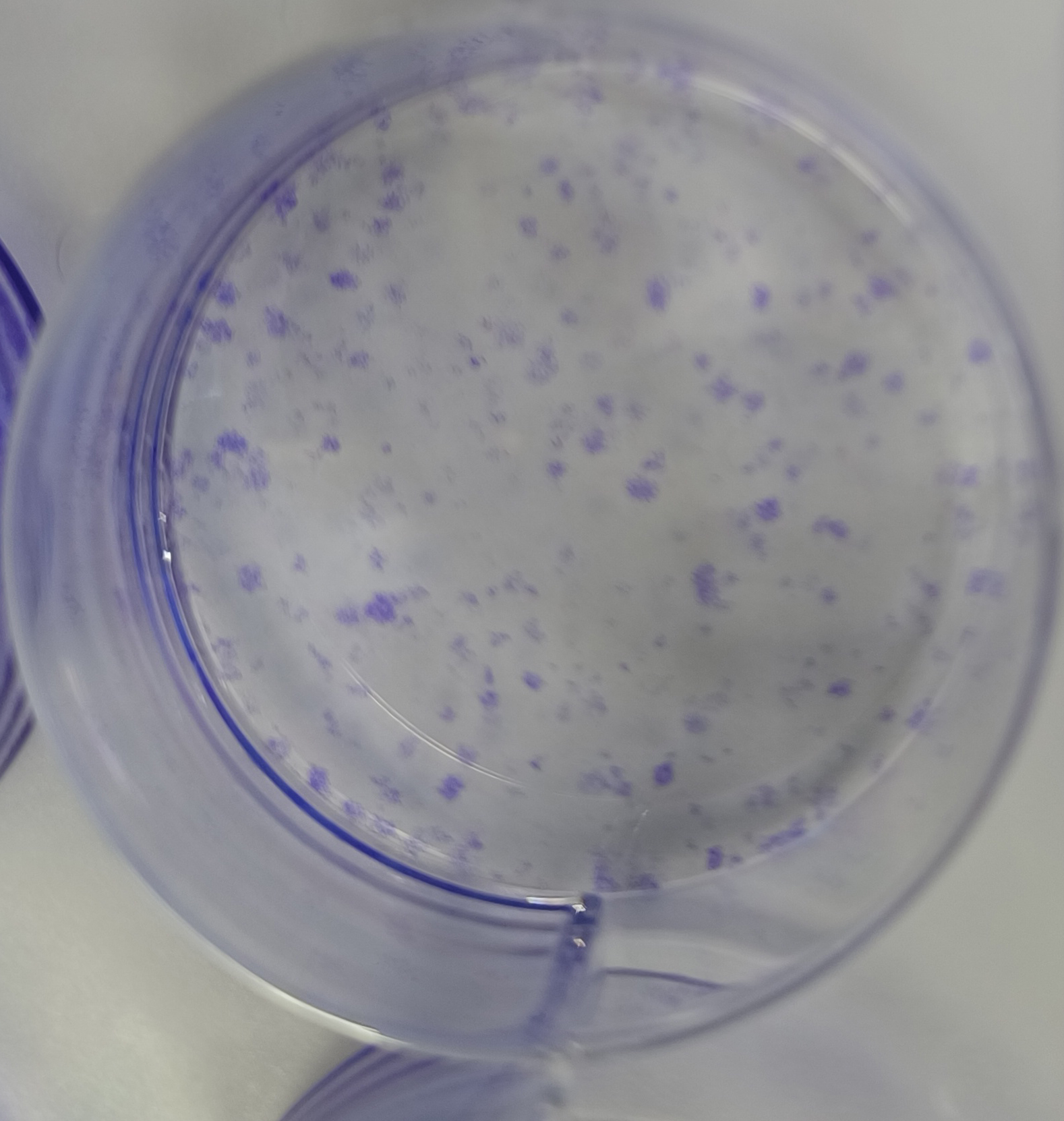

Supplement: Supplementary file 18 [file DataSheet_1.zip › Source data/colony/786-0/siSTAT2-1/3.jpg]

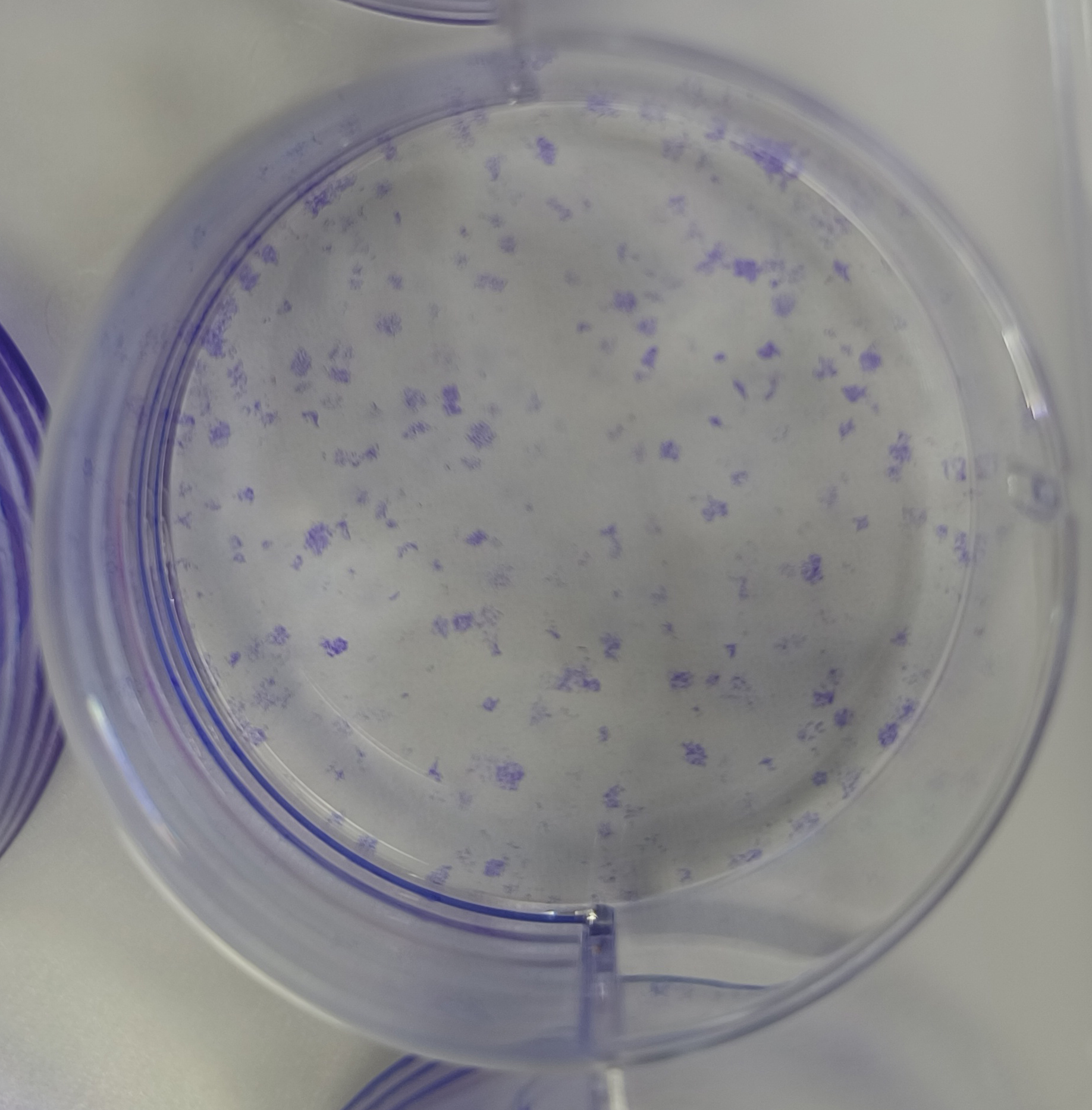

Supplement: Supplementary file 18 [file DataSheet_1.zip › Source data/colony/786-0/siSTAT2-2/1.jpg]

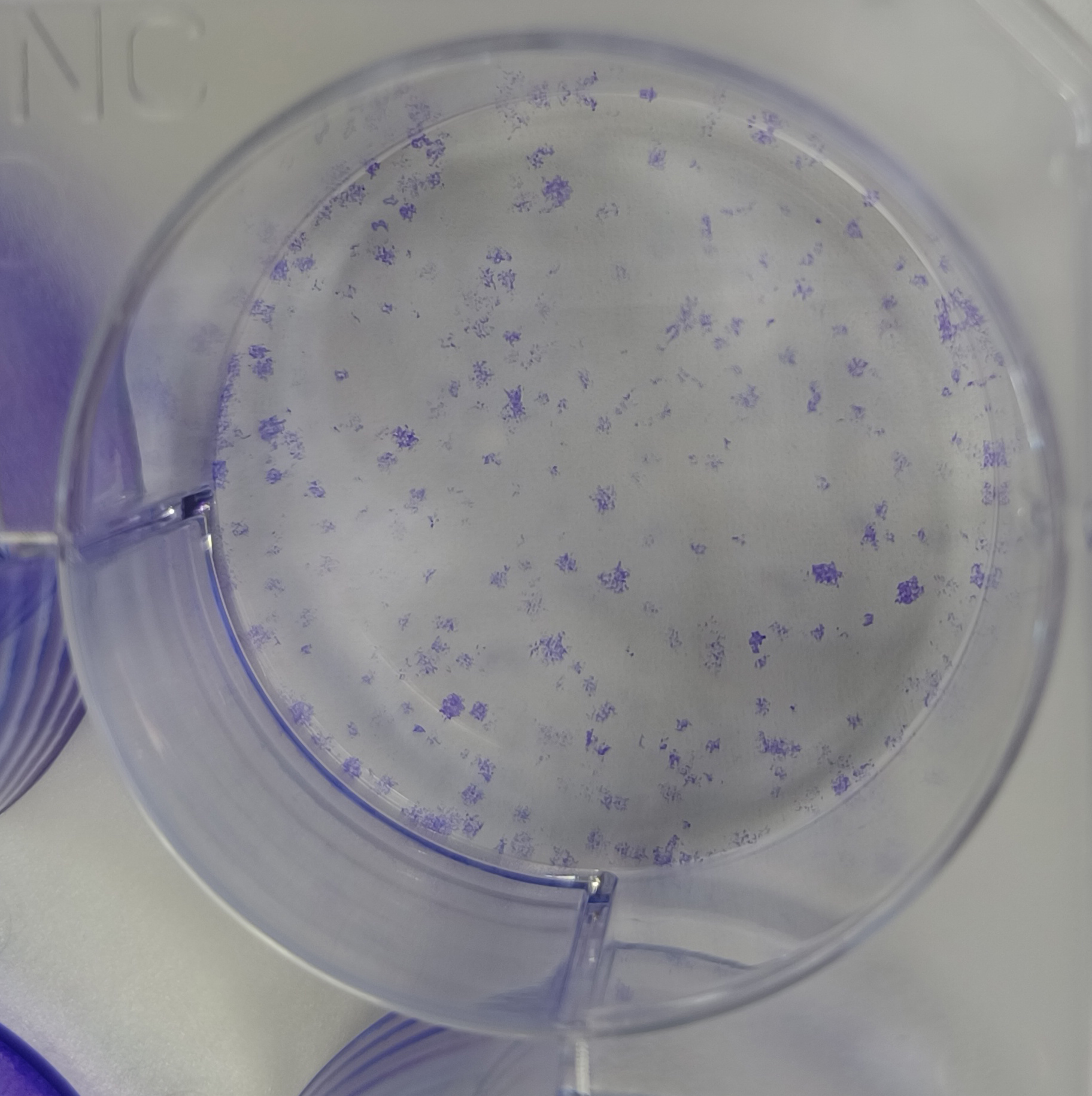

Supplement: Supplementary file 18 [file DataSheet_1.zip › Source data/colony/786-0/siSTAT2-2/2.jpg]

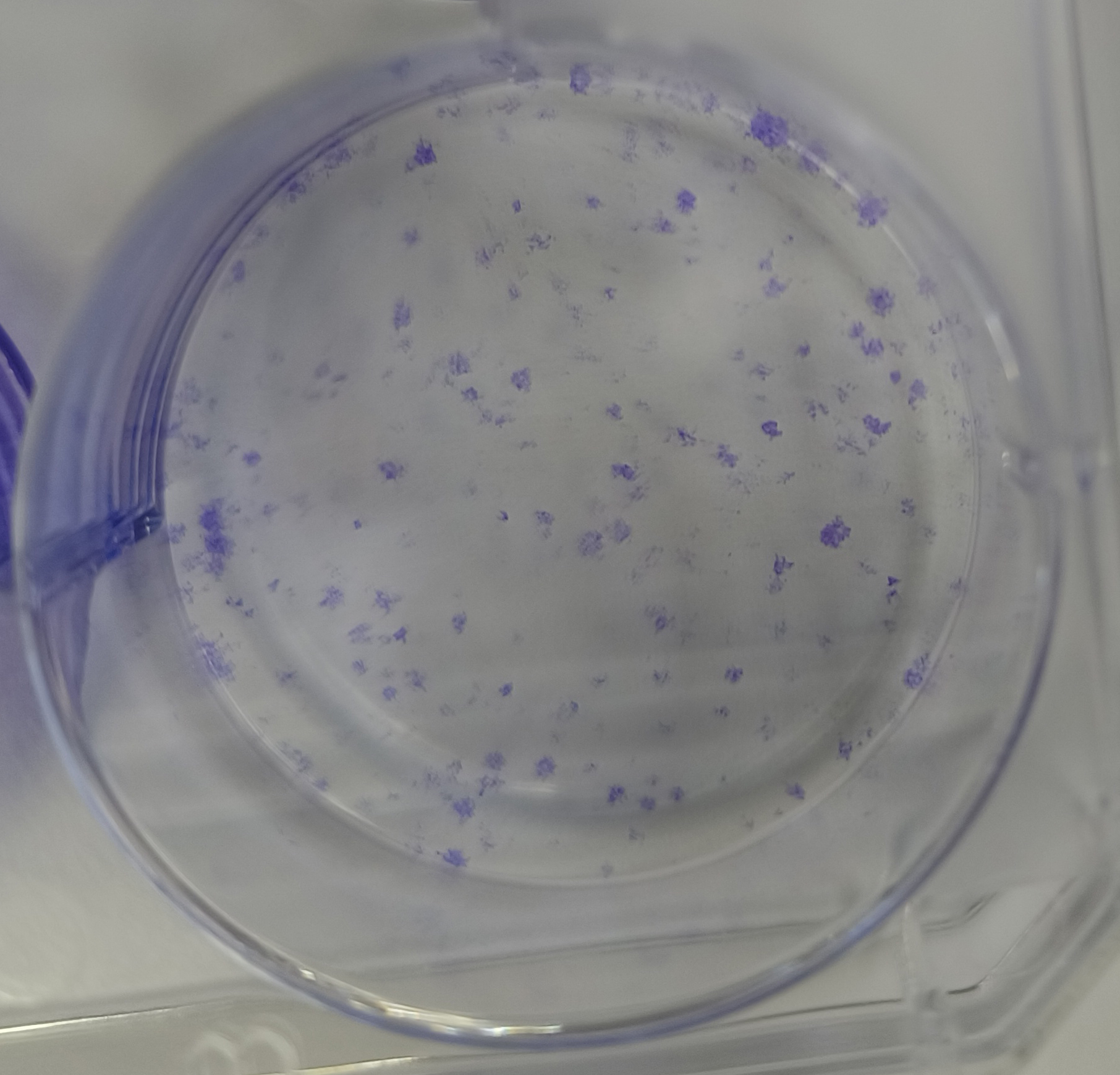

Supplement: Supplementary file 18 [file DataSheet_1.zip › Source data/colony/786-0/siSTAT2-2/3.jpg]

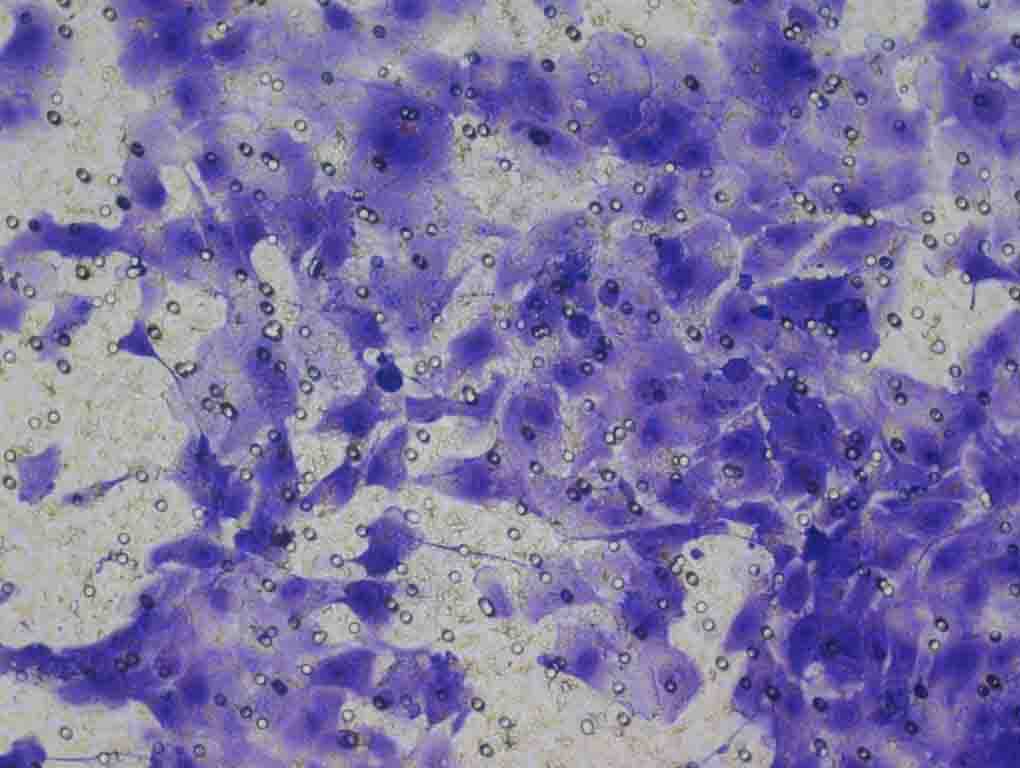

Supplement: Supplementary file 18 [file DataSheet_1.zip › Source data/transwell/769-P/invasion/NC.jpg]

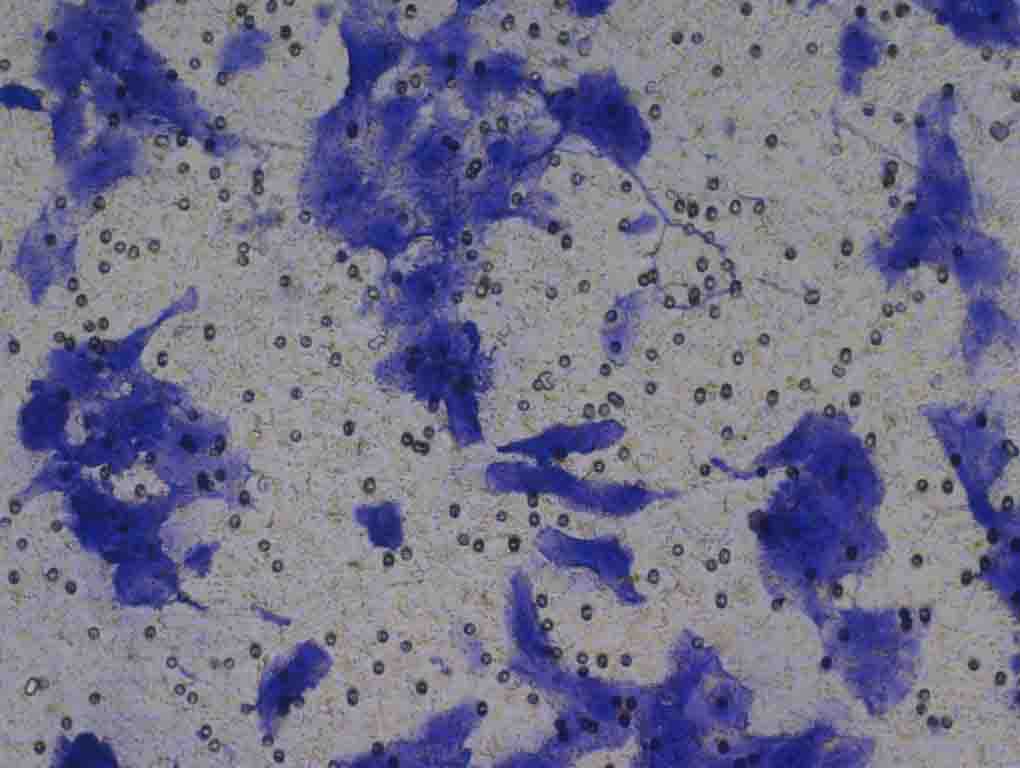

Supplement: Supplementary file 18 [file DataSheet_1.zip › Source data/transwell/769-P/invasion/siSTAT2-1.jpg]

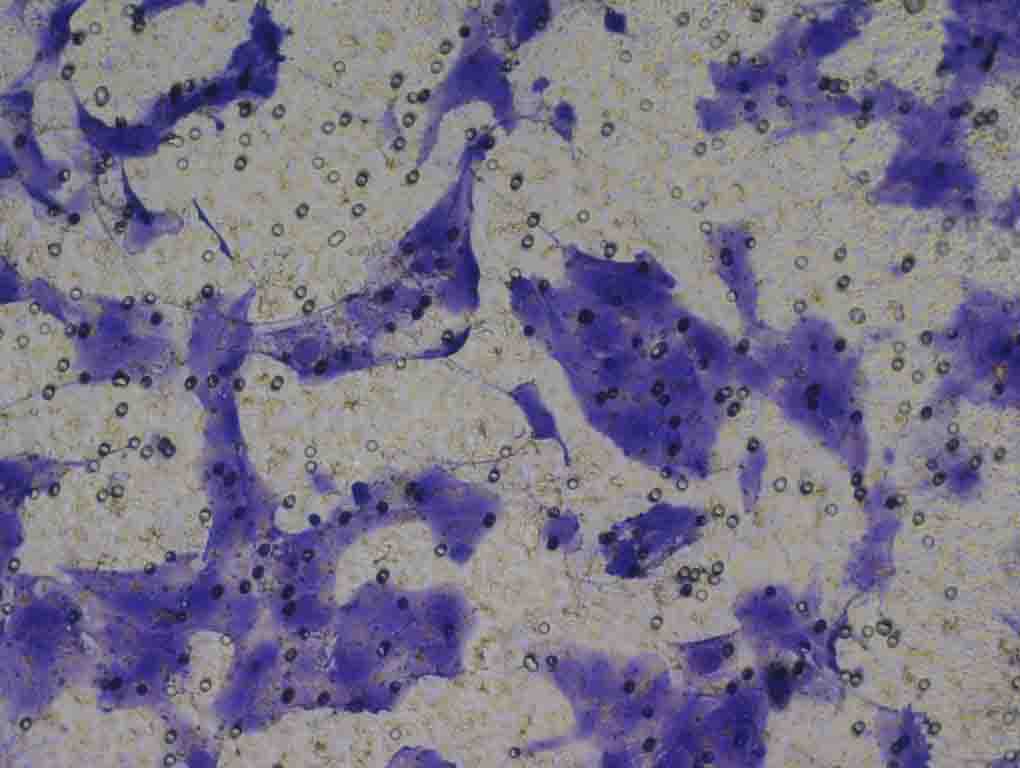

Supplement: Supplementary file 18 [file DataSheet_1.zip › Source data/transwell/769-P/invasion/siSTAT2-2.jpg]

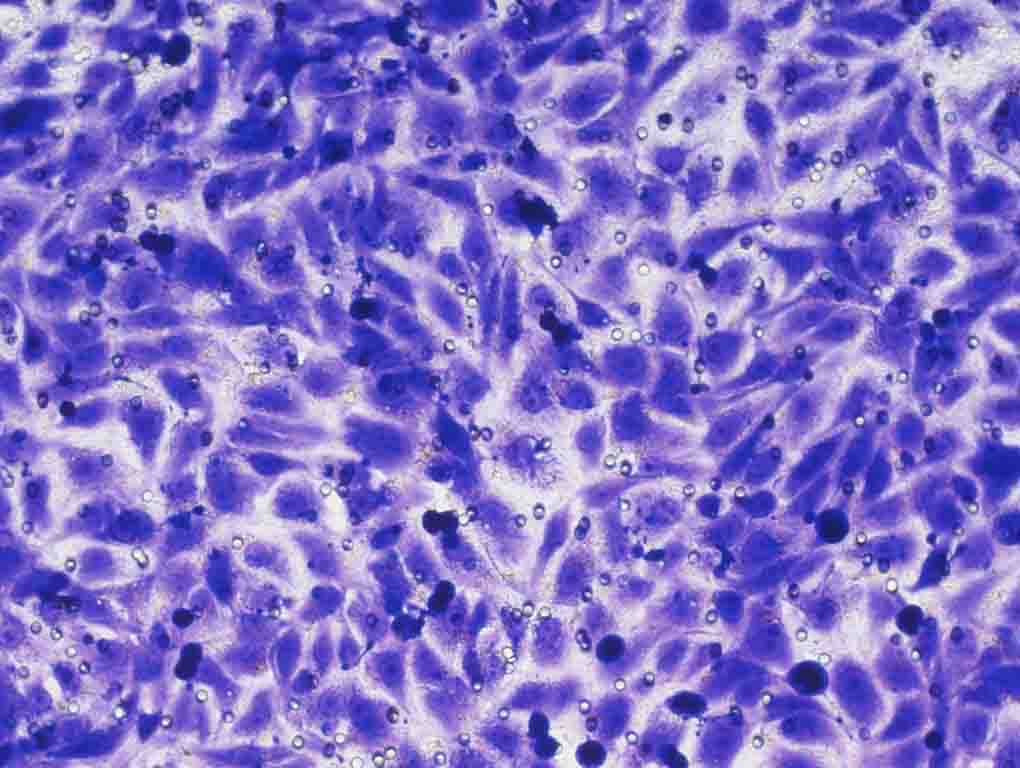

Supplement: Supplementary file 18 [file DataSheet_1.zip › Source data/transwell/769-P/migration/NC.jpg]

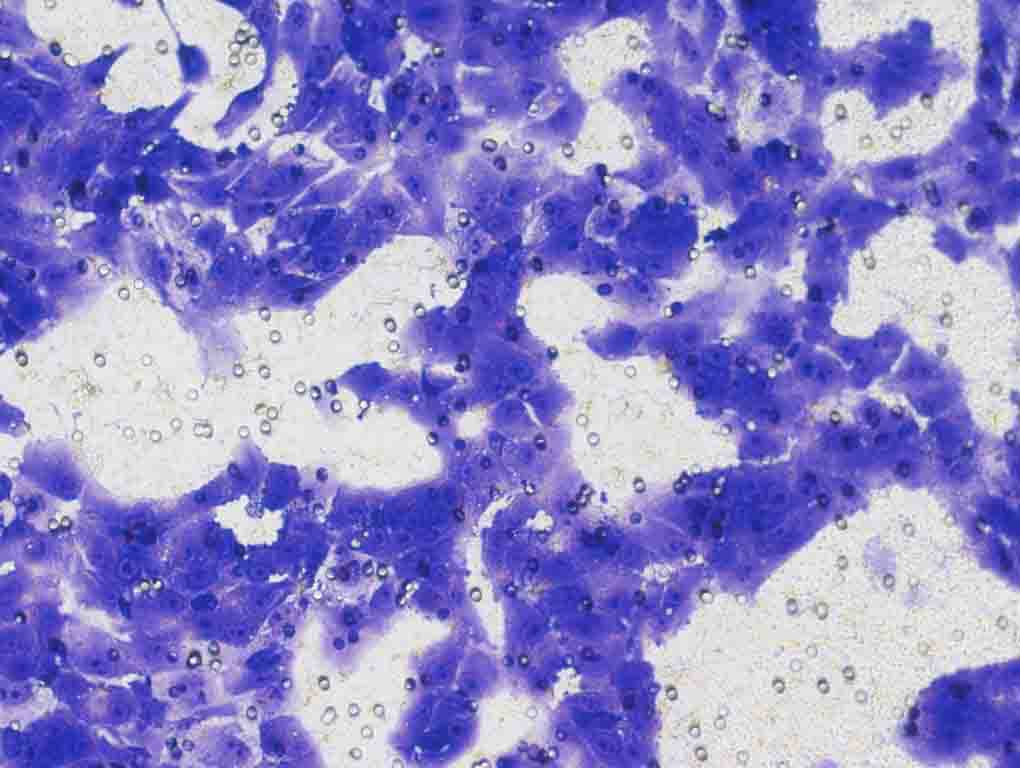

Supplement: Supplementary file 18 [file DataSheet_1.zip › Source data/transwell/769-P/migration/siSTAT2-1.jpg]

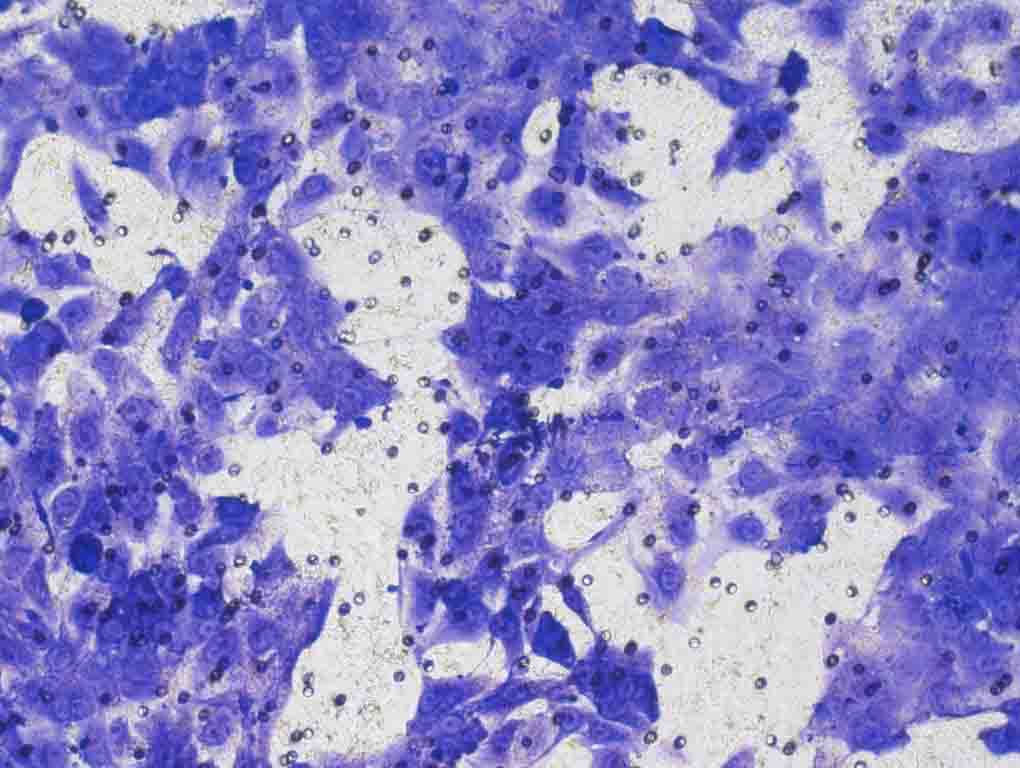

Supplement: Supplementary file 18 [file DataSheet_1.zip › Source data/transwell/769-P/migration/siSTAT2-2.jpg]

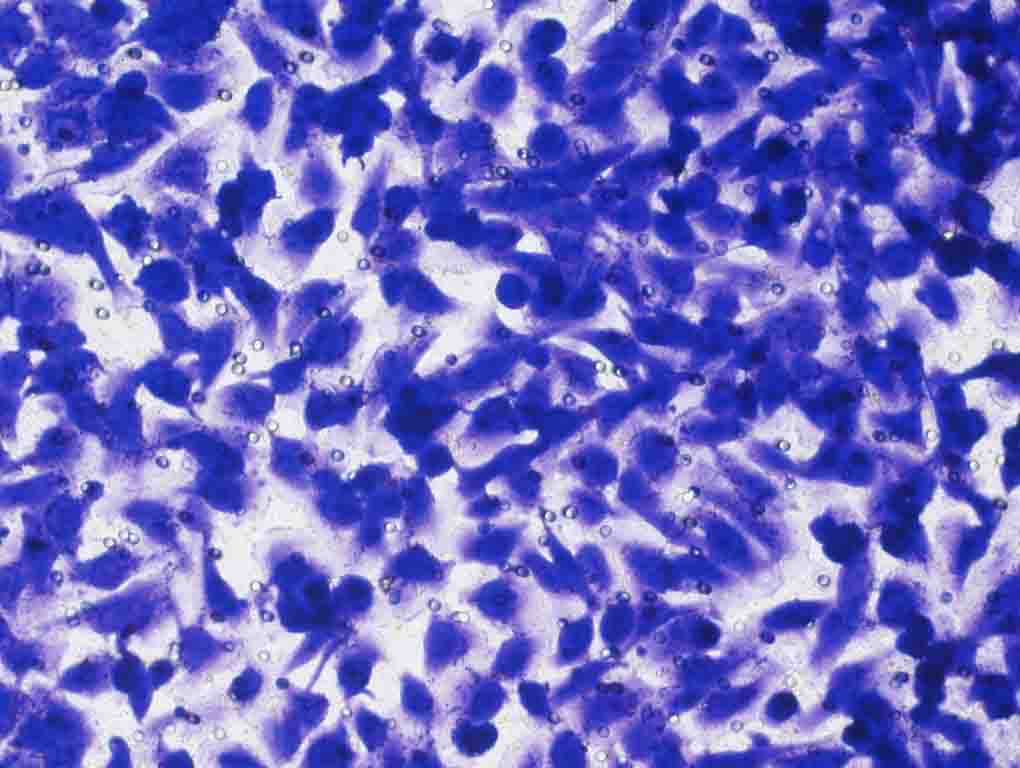

Supplement: Supplementary file 18 [file DataSheet_1.zip › Source data/transwell/786-0/invasion/NC.jpg]

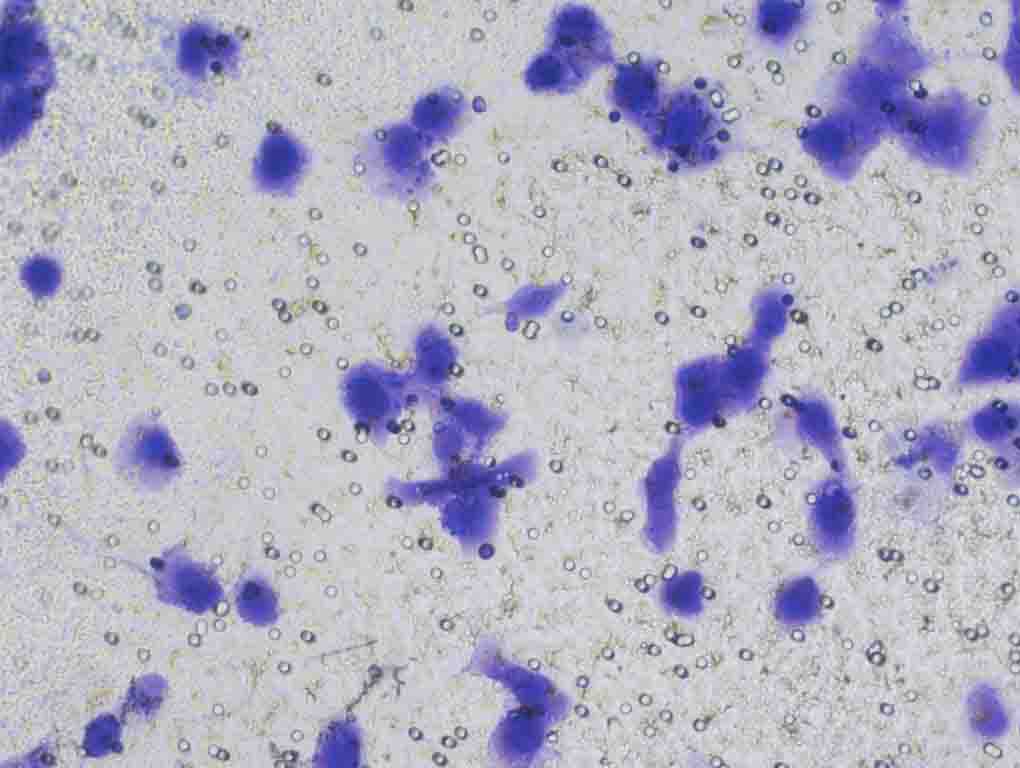

Supplement: Supplementary file 18 [file DataSheet_1.zip › Source data/transwell/786-0/invasion/siSTAT2-1.jpg]

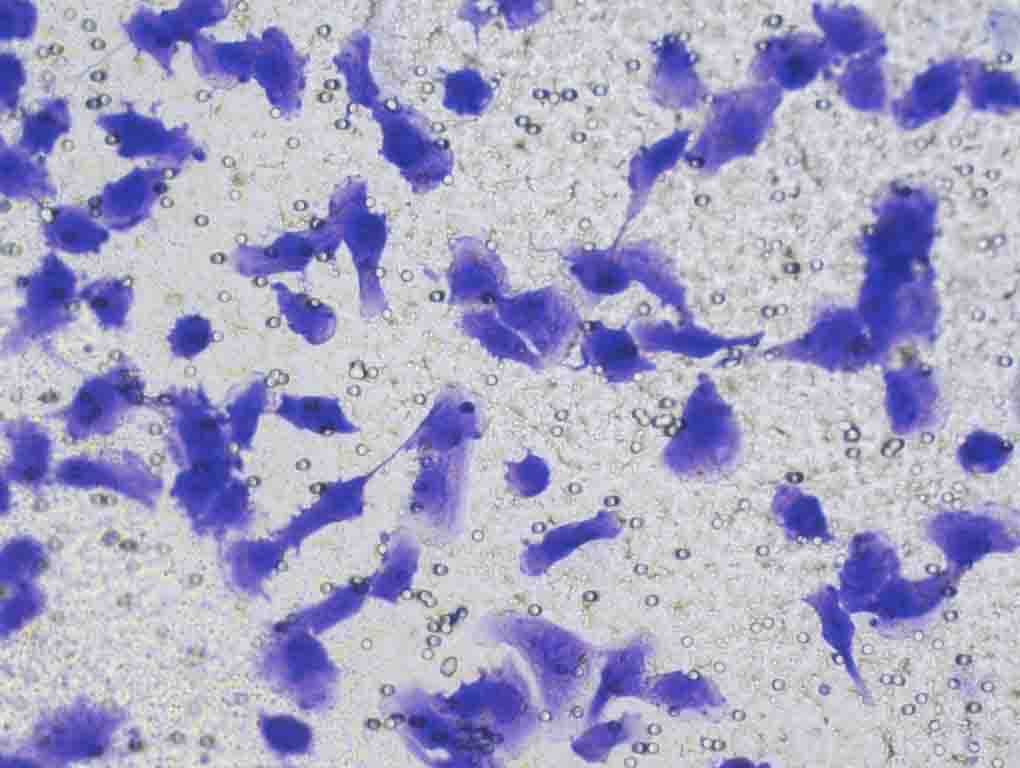

Supplement: Supplementary file 18 [file DataSheet_1.zip › Source data/transwell/786-0/invasion/siSTAT2-2.jpg]

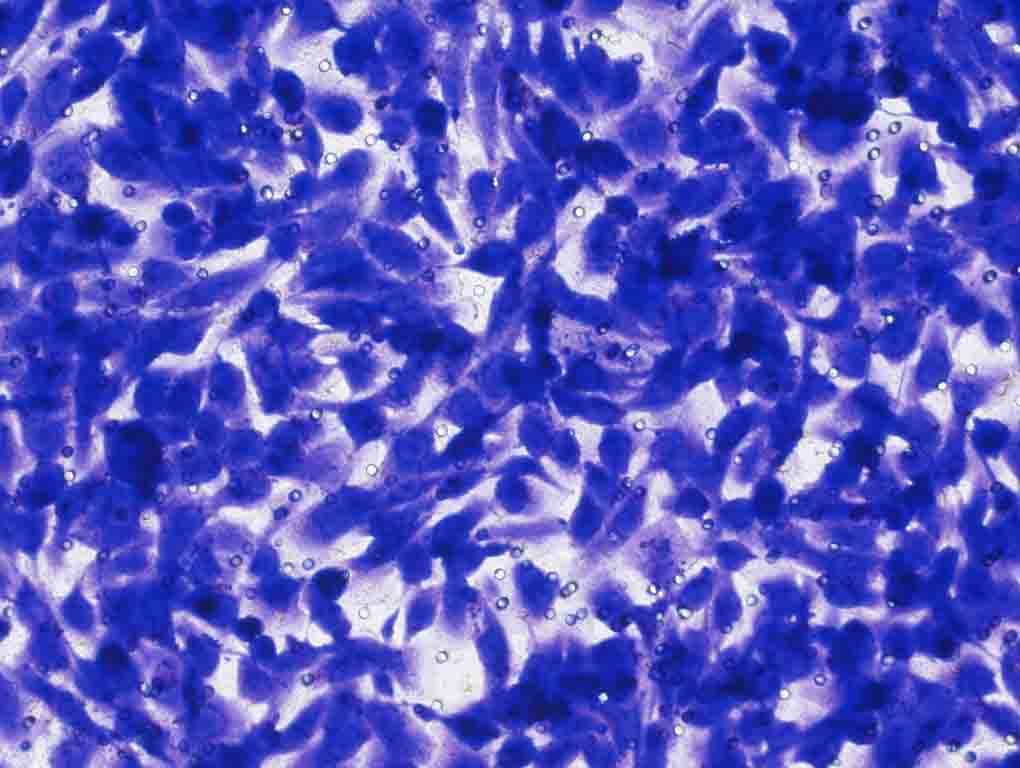

Supplement: Supplementary file 18 [file DataSheet_1.zip › Source data/transwell/786-0/migration/NC.jpg]

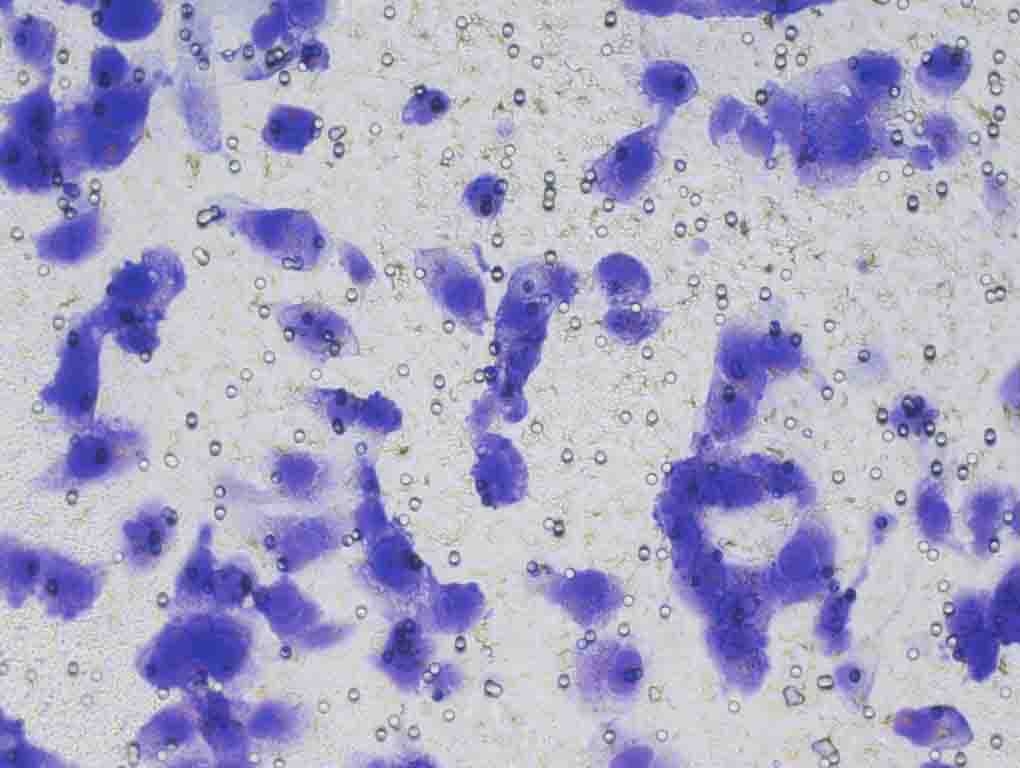

Supplement: Supplementary file 18 [file DataSheet_1.zip › Source data/transwell/786-0/migration/siSTAT2-1.jpg]

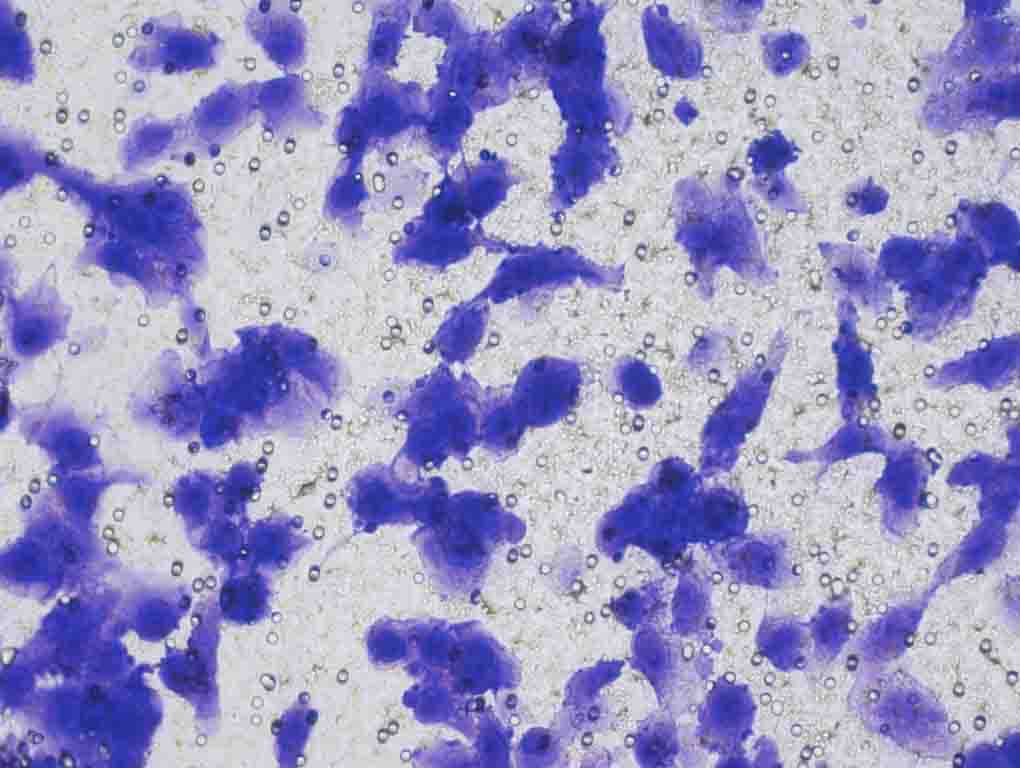

Supplement: Supplementary file 18 [file DataSheet_1.zip › Source data/transwell/786-0/migration/siSTAT2-2.jpg]

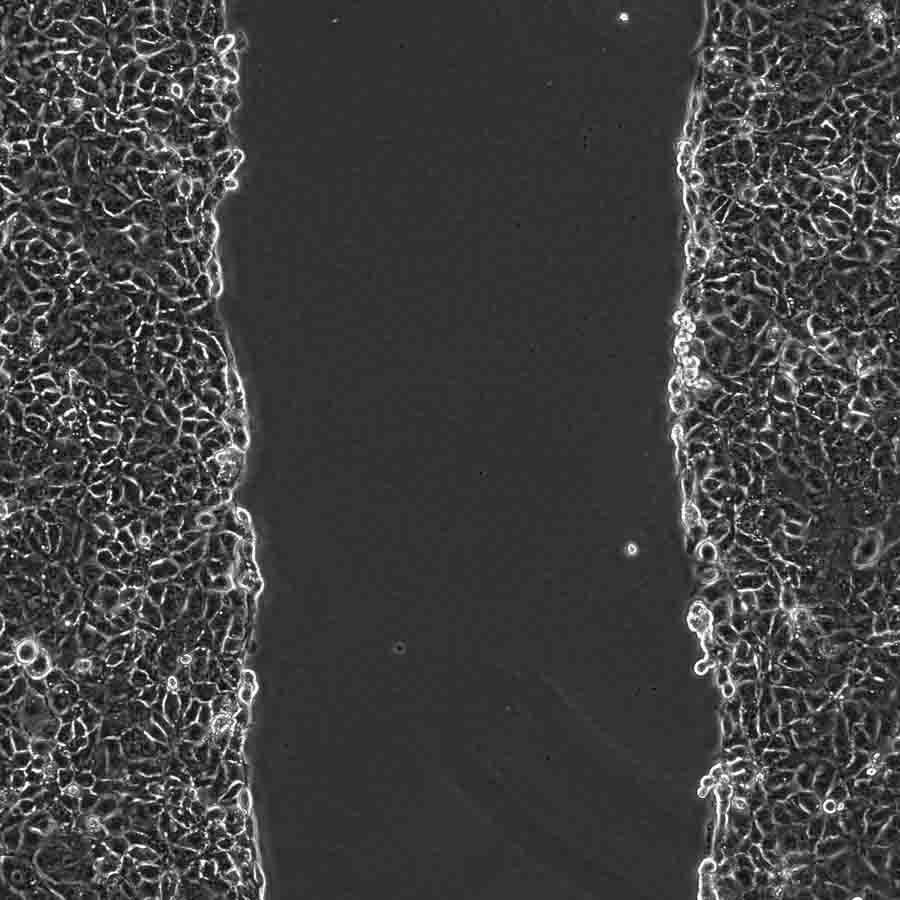

Supplement: Supplementary file 18 [file DataSheet_1.zip › Source data/wound-healing/769-P/0H/NC.jpg]

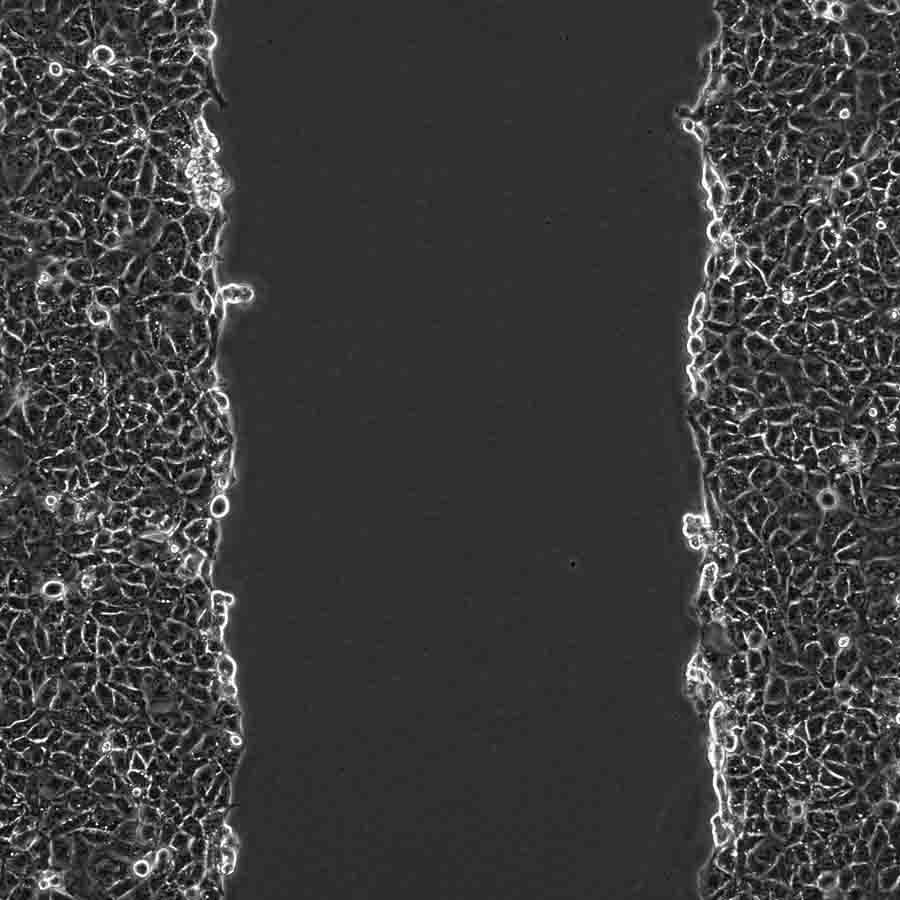

Supplement: Supplementary file 18 [file DataSheet_1.zip › Source data/wound-healing/769-P/0H/siSTAT2-1.jpg]

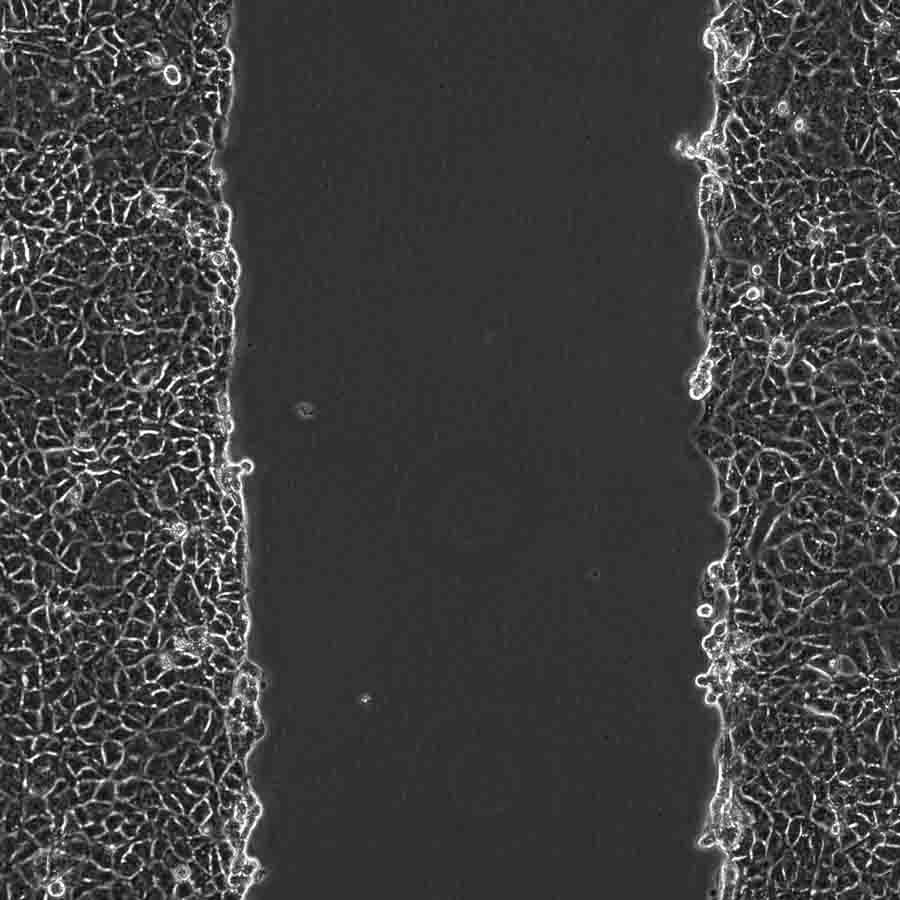

Supplement: Supplementary file 18 [file DataSheet_1.zip › Source data/wound-healing/769-P/0H/siSTAT2-2.jpg]

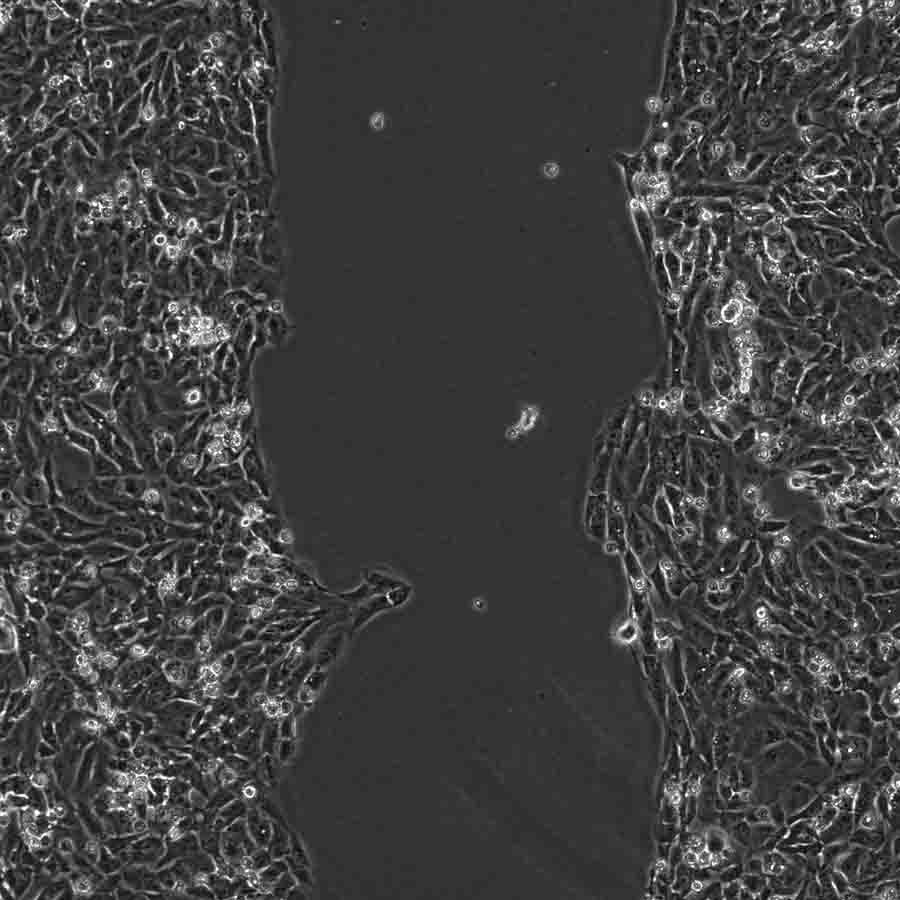

Supplement: Supplementary file 18 [file DataSheet_1.zip › Source data/wound-healing/769-P/24H/NC.jpg]

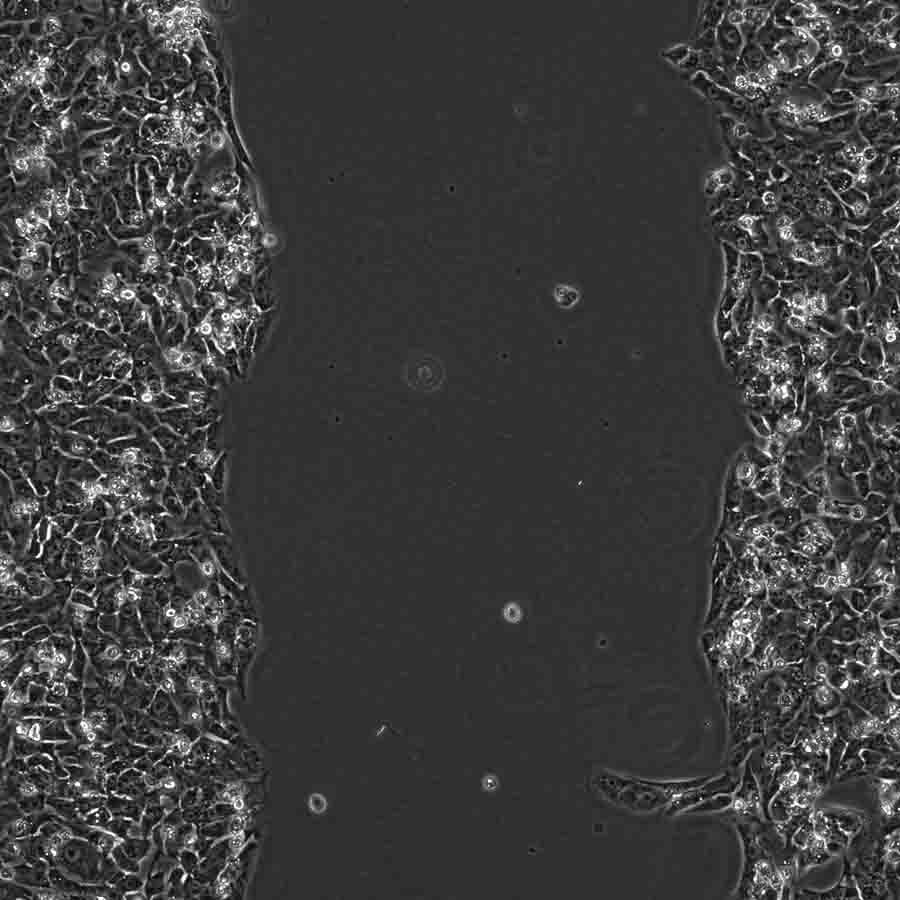

Supplement: Supplementary file 18 [file DataSheet_1.zip › Source data/wound-healing/769-P/24H/siSTAT2-1.jpg]

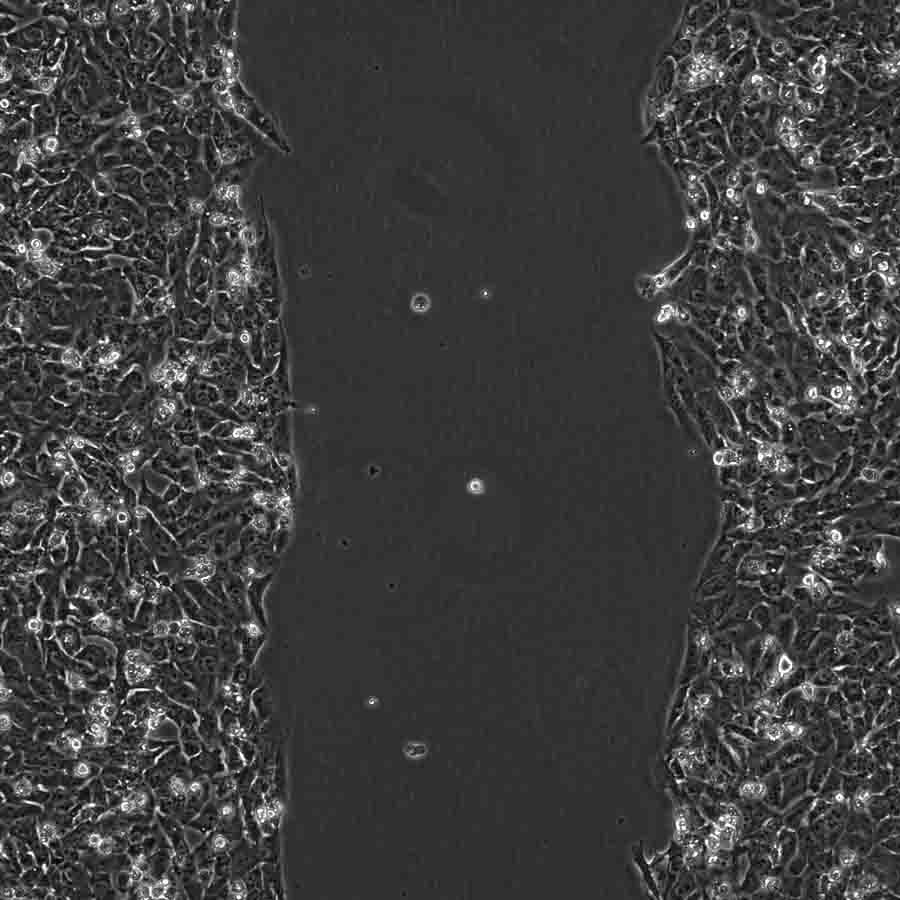

Supplement: Supplementary file 18 [file DataSheet_1.zip › Source data/wound-healing/769-P/24H/siSTAT2-2.jpg]

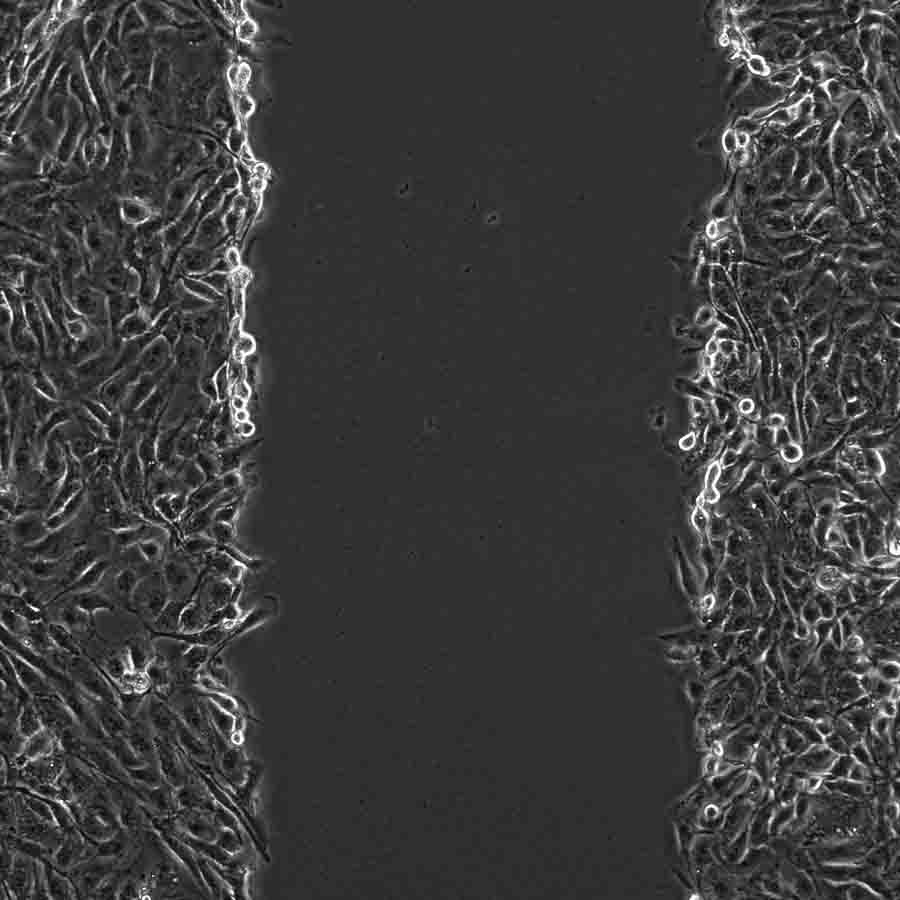

Supplement: Supplementary file 18 [file DataSheet_1.zip › Source data/wound-healing/786-0/0H/NC.jpg]

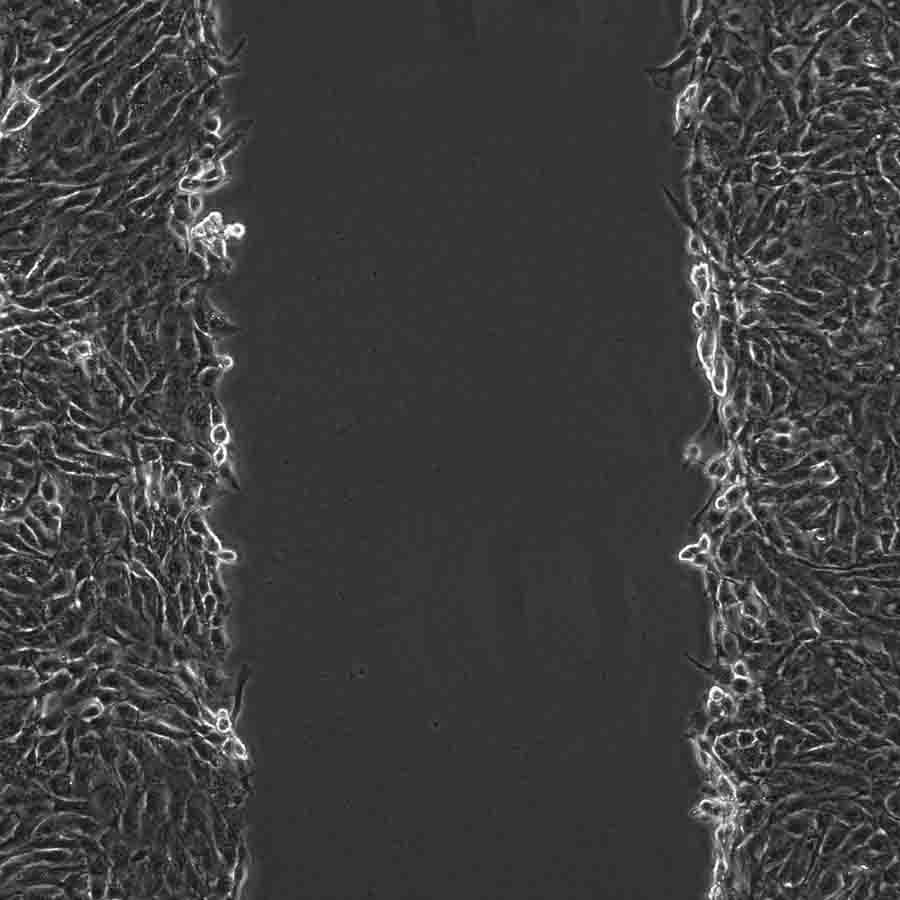

Supplement: Supplementary file 18 [file DataSheet_1.zip › Source data/wound-healing/786-0/0H/siSTAT2-1.jpg]

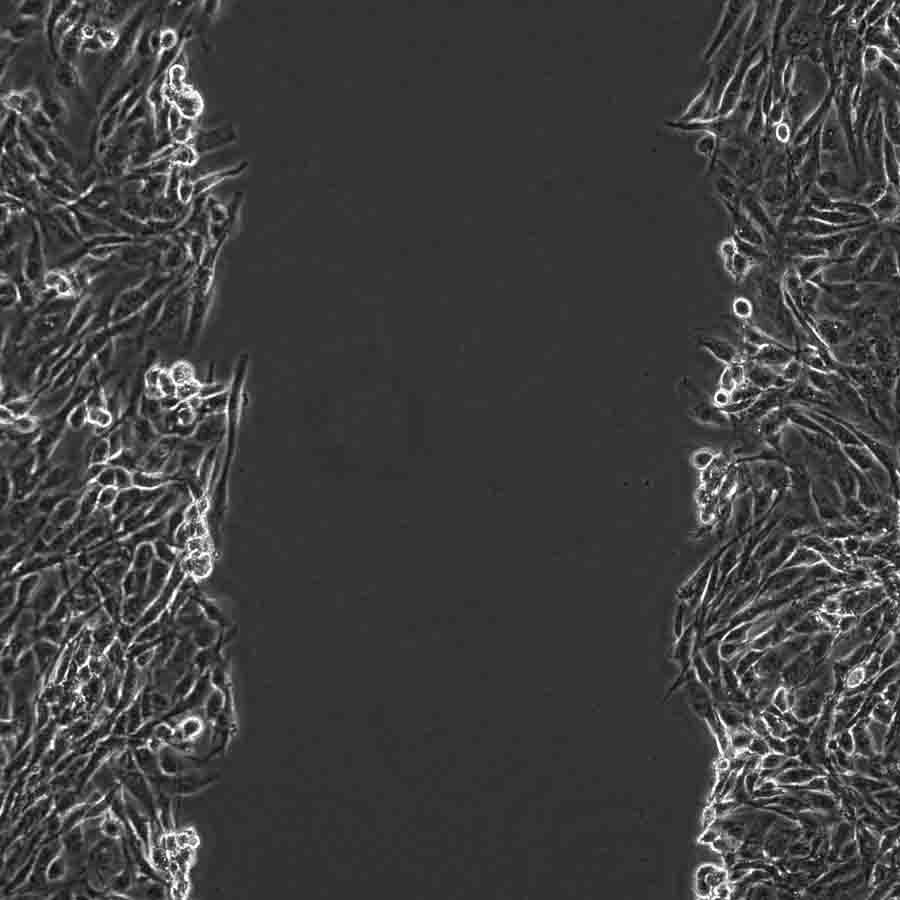

Supplement: Supplementary file 18 [file DataSheet_1.zip › Source data/wound-healing/786-0/0H/siSTAT2-2.jpg]

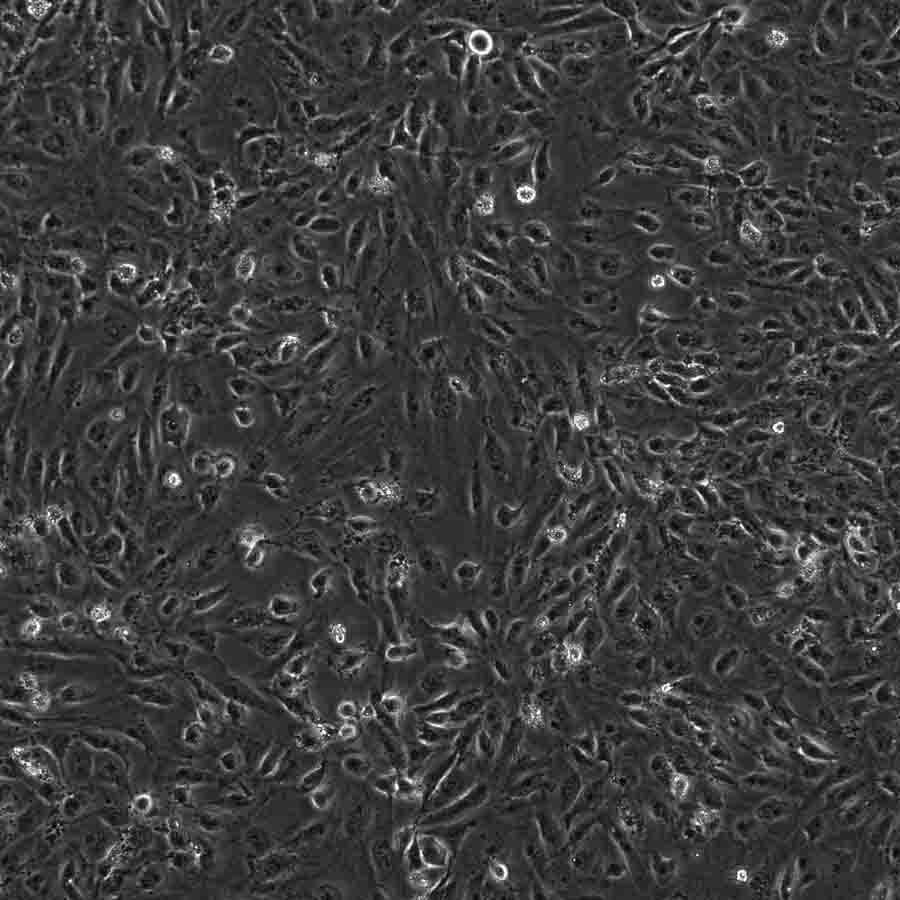

Supplement: Supplementary file 18 [file DataSheet_1.zip › Source data/wound-healing/786-0/24H/NC.jpg]

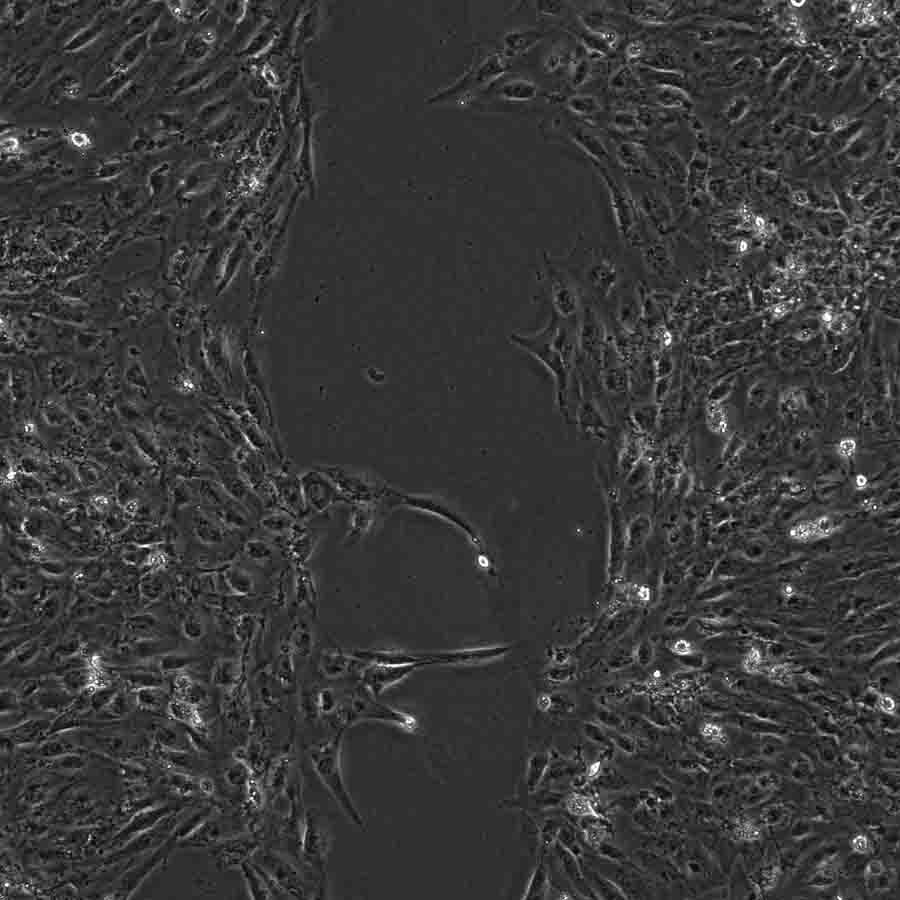

Supplement: Supplementary file 18 [file DataSheet_1.zip › Source data/wound-healing/786-0/24H/siSTAT2-1.jpg]

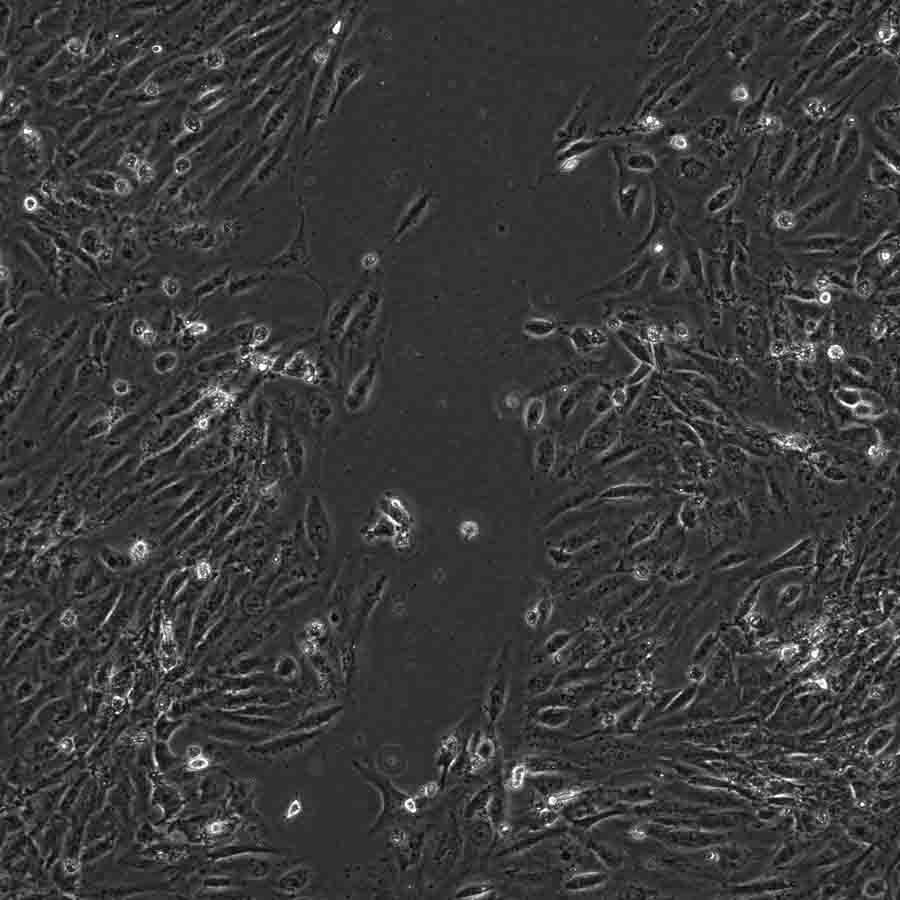

Supplement: Supplementary file 18 [file DataSheet_1.zip › Source data/wound-healing/786-0/24H/siSTAT2-2.jpg]
